# Chalcogen-Influenced Benzochalcogenazolo-Based *N*,*O*‑Coordinated Difluoroboron Complexes: From Lasing Dyes to Room-Temperature-Phosphorescence Emitters

**DOI:** 10.1021/acs.inorgchem.5c02557

**Published:** 2025-07-07

**Authors:** Stepan Kutsiy, Radosław Pytlarz, Andrii Hotynchan, Paulina H. Marek-Urban, Roman Luboradzki, Enzo Jean-Woldemar, Dmytro Volyniuk, Sébastien Chénais, Sébastien Forget, Krzysztof Durka, Juozas V. Grazulevicius, Mykhaylo A. Potopnyk

**Affiliations:** † Institute of Organic Chemistry, 49559 Polish Academy of Sciences, Kasprzaka 44/52, 01-224 Warsaw, Poland; ‡ Department of Electronic Devices, 226328 Lviv Polytechnic National University, Sviatoho Yura sq. 1, Lviv 79013, Ukraine; § Department of Organic Chemistry, Faculty of Chemistry, 112865 Ivan Franko National University of Lviv, Kyryla and Mefodia 6, Lviv 79005, Ukraine; ∥ Faculty of Chemistry, 49566 Warsaw University of Technology, Noakowskiego 3, 00-664 Warsaw, Poland; ⊥ Institute of Physical Chemistry, Polish Academy of Sciences, Kasprzaka 44/52, 01-224 Warsaw, Poland; # Laboratoire de Physique des Lasers, 27097 Université Sorbonne Paris Nord, CNRS, UMR 7538, F-93430 Villetaneuse, France; ∇ Department of Polymer Chemistry and Technology, Kaunas University of Technology, Barsausko 59, LT-51423 Kaunas, Lithuania

## Abstract

Four *N*,*O*-coordinated benzochalcogenazolo-based boron difluoride complexes were designed, synthesized, and spectroscopically investigated in solutions, crystalline state, and dye-doped poly­(methyl methacrylate) films. The single crystal analysis revealed that oxadiazaborinine dyes adopt planar geometry with multiple intermolecular hydrogen-bonding interactions. The benzoxazole- and benzothiazole-containing dyes exhibit highly intensive photoluminescence in the crystalline state and when dispersed in the inert polymer, accompanied by high-rate constants of radiative deactivation (2.2 × 10^8^ s^–1^ and 2.7 × 10^8^ s^–1^, respectively). This results in their amplified spontaneous emission ability with very low thresholds of 11 and 4.7 μJ/cm^2^, respectively, and very narrow bandwidths with the values of full widths at half maxima of 10 and 6.3 nm. In stark contrast, the benzotellurium-containing analogue shows room temperature phosphorescence with a short phosphorescence lifetime of 9.4 μs, caused by the strong chalcogen heavy-atom effect.

## Graphical abstract

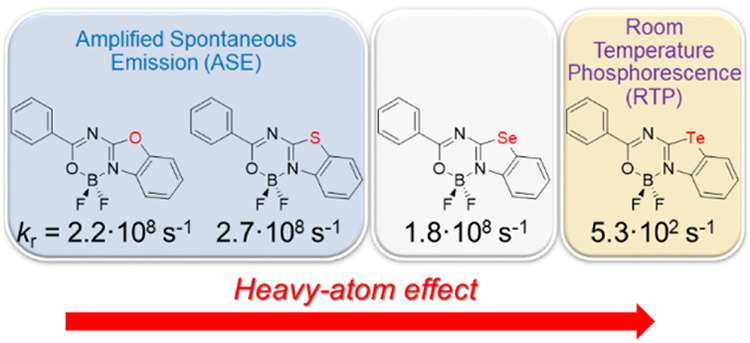

## Introduction

Luminescent dyes that contain chalcogen atoms represent a broad and versatile class of organic compounds with diverse photophysical behaviors.^1^ Among them, oxygen- and sulfur-containing luminescent dyes have been extensively investigated.^2^ In contrast, dyes with selenium and tellurium remain underdeveloped because of their lower stability and highly challenging synthesis.^3^ Nevertheless, some information on selenium and tellurium-containing luminescent dyes was provided by several research groups.^4–10^ The main advantage of incorporating heavy chalcogen atoms such as selenium or tellurium is an increase of their spin–orbit coupling (SOC) between singlet and triplet excited states.^11–17^ This is especially beneficial for applications of such materials as photosensitizers in photodynamic therapy.^18,19^ Another interesting feature of dyes containing heavy chalcogens is room temperature phosphorescence (RTP),^20^ making them suitable for the application as emitters in organic light-emitting diodes (OLEDs).^21–25^

On the other hand, nowadays, one of the most intensively investigated class of organic chromophores are boron difluoride complexes.^26,27^ They found different applications in materials science, including OLEDs,^28,29^ organic lasers,^30–32^ organic photodetectors,^33^ stimuli-responsive materials,^34,35^ sensors,^36^ etc. Incorporation of heavy chalcogen atom into the boron difluoride dye structure strongly enhances SOC, enabling potential applications in optoelectronics and photodynamic therapy (PDT). For instance, derivatives of 4,4-difluoro-4-bora-3a,4a-diaza-*s*-indacene (commonly known as boron dipyrromethene, or BODIPY) with selenium-containing substituents at positions 2 and 6 were synthesized, and their photophysical properties were investigated, revealing a moderate involvement of triplet states in the photophysical processes.^37^ Some tellurium-containing boron dyes were investigated for their application in medicine, in particular in bioimaging, detection of reactive oxygen species, and PDT. Thus, tellurium-containing BODIPY^38^ and aza-BODIPY^39^ show a great potential in reactive oxygen species detection in the experiments with oxidizers such as hypochlorous acid or hydrogen peroxide. In contrast, the phosphorescence ability of nonpyrrole-based boron difluoride complexes is less investigated.^40–42^

Our ongoing interest in photoactive materials has led us to the development of the *N*,*O*-chelated boron difluoride complexes based on (benzo)­thiazole-fused [1,3,5,2]­oxadiazaborinine core.^43–45^ In general, such chromophores possess diverse properties, including solid-state emission (SSE),^46^ aggregation-induced emission (AIE),^47–49^ and thermally activated delayed fluorescence,^29^ paving the way for their application in bioimaging and optoelectronics.

## Results and Discussion

### Molecular Design and Synthesis

In this work, we present a series of boron difluoride complexes based on benzochalcogenazoles incorporating oxygen, sulfur, selenium, and tellurium atoms (Figure 1). The obtained compounds were thoroughly characterized, including single crystal analysis, electrochemical measurements, and evaluation of optical properties supported by theoretical calculations. According to our results, oxygen- and sulfur-containing derivatives display amplified spontaneous emission (ASE)^50^ with very low thresholds, while tellurium-based analogues exhibit RTP with short-microsecond-scale lifetimes.

**Figure 1:**
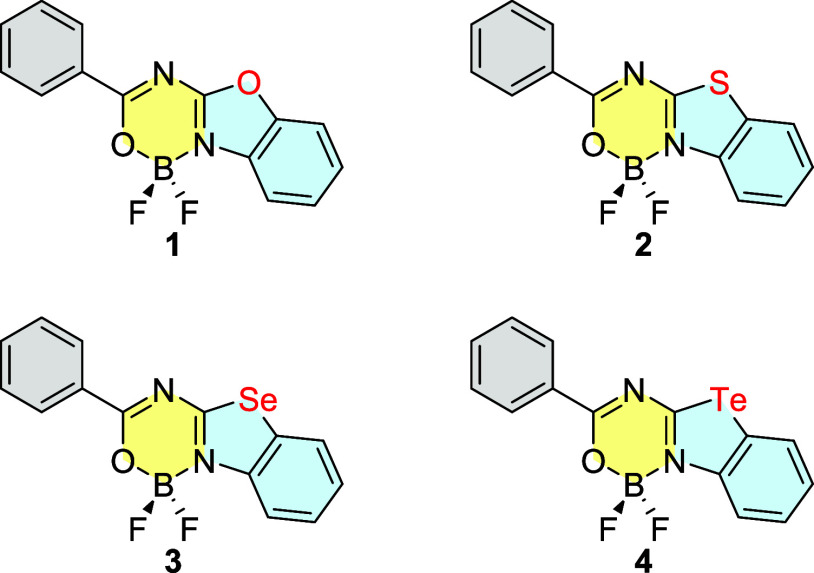
Benzochalcogenazolo-based boron difluoride complexes **1–4**.

The synthesis of compounds **1–4** (Scheme 1) started from benzoyl chloride (**5**). It was converted to benzoyl isothiocyanate (**6**), which in turn was reacted *in situ* with 2-aminophenol (**7**), resulting in *N*-((2-hydroxyphenyl)­carbamothioyl)­benzamide (**8**) in 77% total yield. Analogous reactions of benzoyl isothiocyanate with 2-aminobenzenethiol (**11**) or 2,2′-diselanediyldianiline (**14**)^51^ gave intermediates **12** and **15**, which were simultaneously converted into amides **13** and **16**, respectively, with good yields (69 and 78%). In contrast, the reaction between isothiocyanate **6** and 2-aminobenzenetellurol (**17**) results in the formation of *N*-((2-hydrotellurophenyl)­carbamothioyl)­benzamide (**18**), which was isolated in a yield of 63%. To obtain the benzoxazole- and benzotellurazole-containing ligands, thioureas **8** and **18** were treated with Burgess reagent [methyl *N*-(triethylammoniumsulfonyl)­carbamate, **9**] to give the products **10** and **19** in 89 and 44% yields, respectively. Finally, amides **10**, **13**, **16**, and **19** were treated with boron trifluoride etherate (BF_3_·OEt_2_) in the presence of *N*,*N*-diisopropylethylamine (DIPEA) providing oxadiazaborinines **1**, **2**, **3**, and **4** in yields of 70, 72, 72, and 45%, respectively. The synthesized boron dyes and their precursors were characterized by ^1^H, ^13^C, ^19^F, ^77^Se, and ^125^Te NMR spectroscopy and high-resolution mass spectrometry (HRMS).

**Scheme 1:**
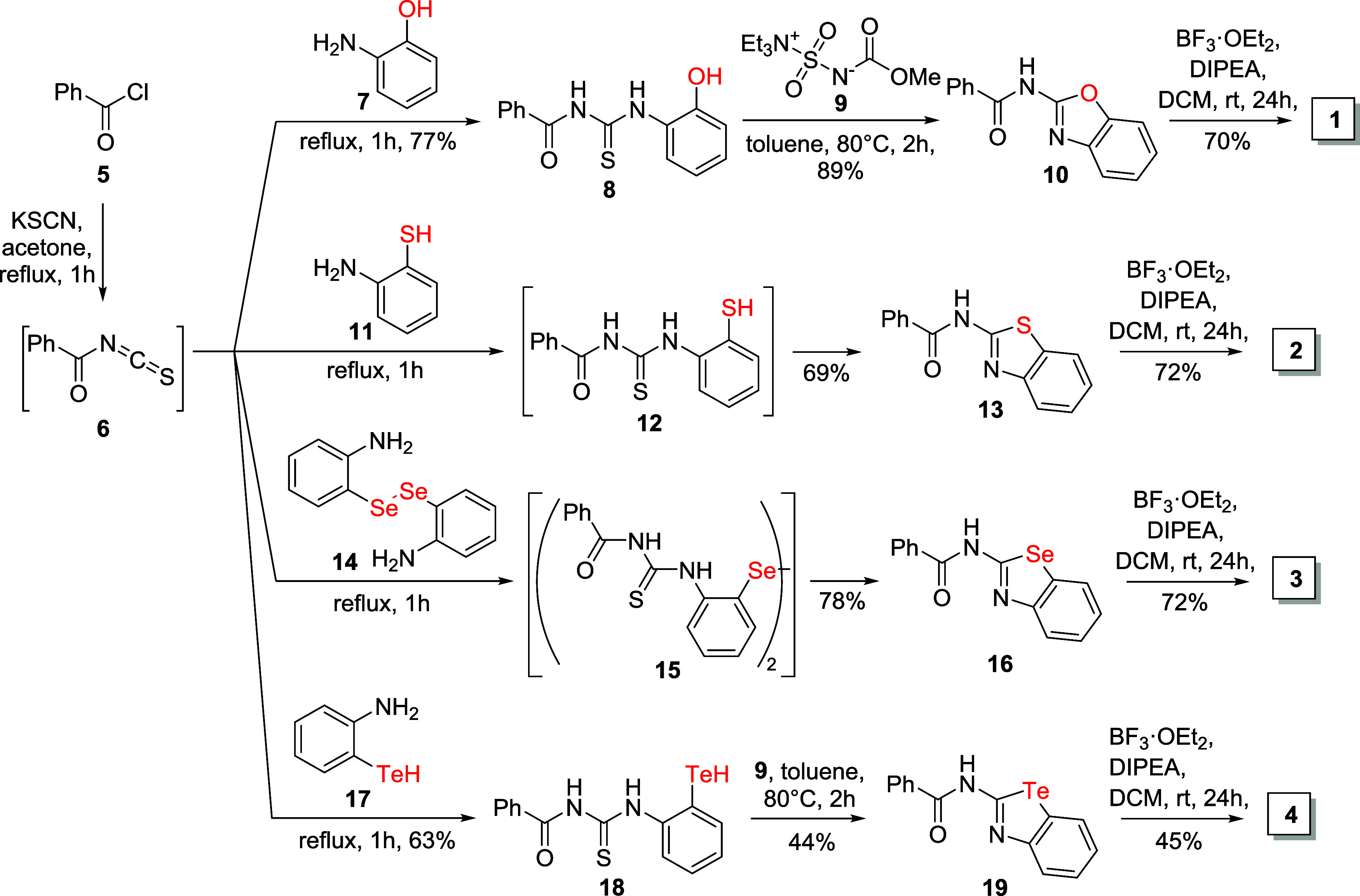
Synthesis of Boron Difluoride Complexes **1–4**

### Crystal Structure Analysis

The crystal structures of boron complexes **1–4** were confirmed by single-crystal X-ray diffraction technique (Figures S1–S4 and Tables S1–S4 in the Supporting Information, SI). In all four structures, pendant phenyl groups adopt near-to-coplanar conformation with respect to heterocyclic units, as evidenced by the torsion angles varying from 0.5° (one of the conformers of compound **2**) to 10.5° (structure **3**). Interestingly, the C-X-C angle (X = chalcogen) in the benzochalcogenazole ring of structures **1–3** significantly decreases from 105.4° for the benzoxazole derivative to 90.3, 85.6, and 79.7° for benzothiazole, benzoselenazole, and benzotellurazole analogues, respectively (Table S5 in the SI).

Compound **1** crystallizes in a monoclinic crystal system (Table S1 and Figure S5 in the SI) with four molecules in the unit cell. The analysis of the supramolecular structure revealed two main crystal motives. Alternated antiparallel molecules stack in columns via π···π/*n*···π interactions (*e* = 3.371 and *f* = 3.395 Å, Figure 2b,c). Meanwhile, in the horizontal plane, the neighboring molecules are engaged in CH···F (*a* = 2.496 Å, *b* = 2.571 Å, *c* = 2.417 Å) and CH···O (*d* = 2.687 Å) hydrogen bonds (Figure 2a).

**Figure 2:**
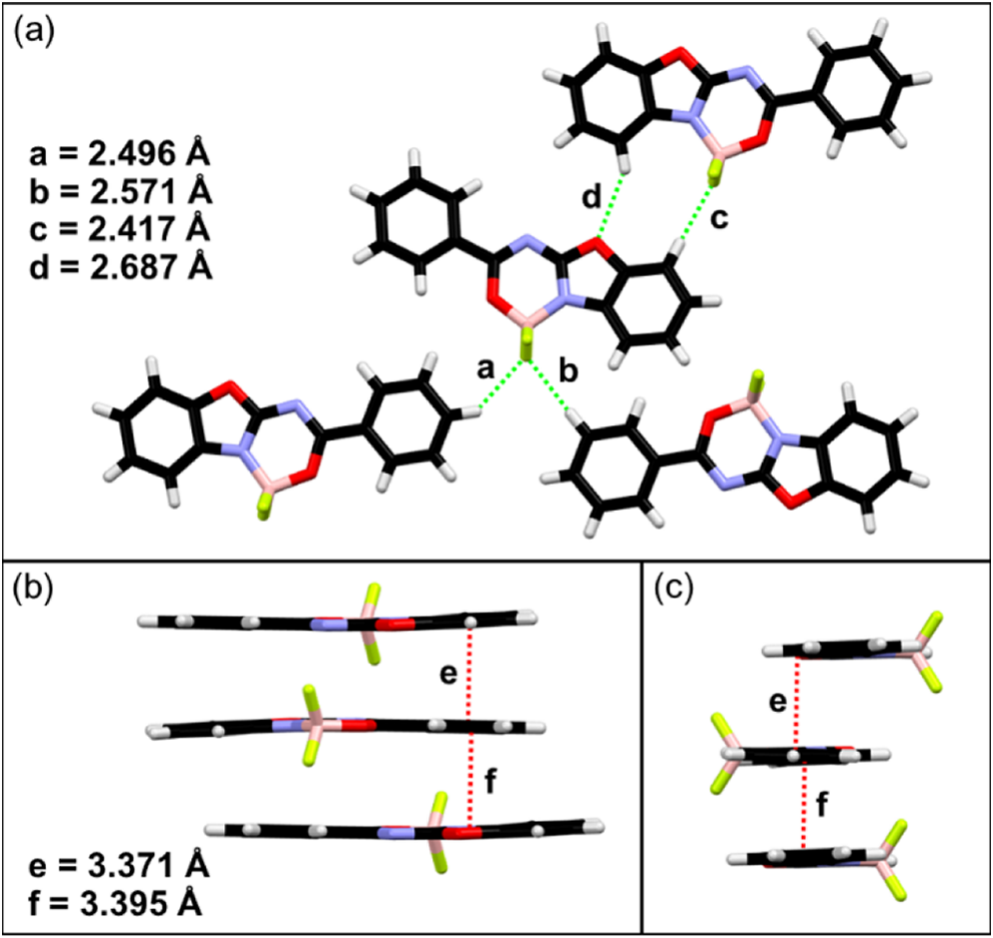
Intermolecular interactions in the crystal structure of dye **1**: CH···F and CH···O hydrogen bonds (a); π···π/*n*···π interactions, front view (b) and side view (c).

Crystal structure **2** acquires a monoclinic crystal system (Table S2 in the SI) with two independent molecules in the asymmetric part of the unit cell. Therefore, the unit cell consists of 8 molecules (Figure S6 in the SI). The molecules of each conformer form different stacking columns with “head-to-head” (*e* = 3.512 Å and *f* = 3.500 Å) and “head-to-tail” orientation (*g* = 3.450 Å and *h* = 3.367 Å, Figure 3b,c). Analogically to the structure of dye **1**, the molecules from neighboring columns interact via CH···F (*a* = 2.634 Å, *b* = 2.403 Å, *c* = 2.620 Å) and CH···S hydrogen bonds (Figure 3a).

**Figure 3:**
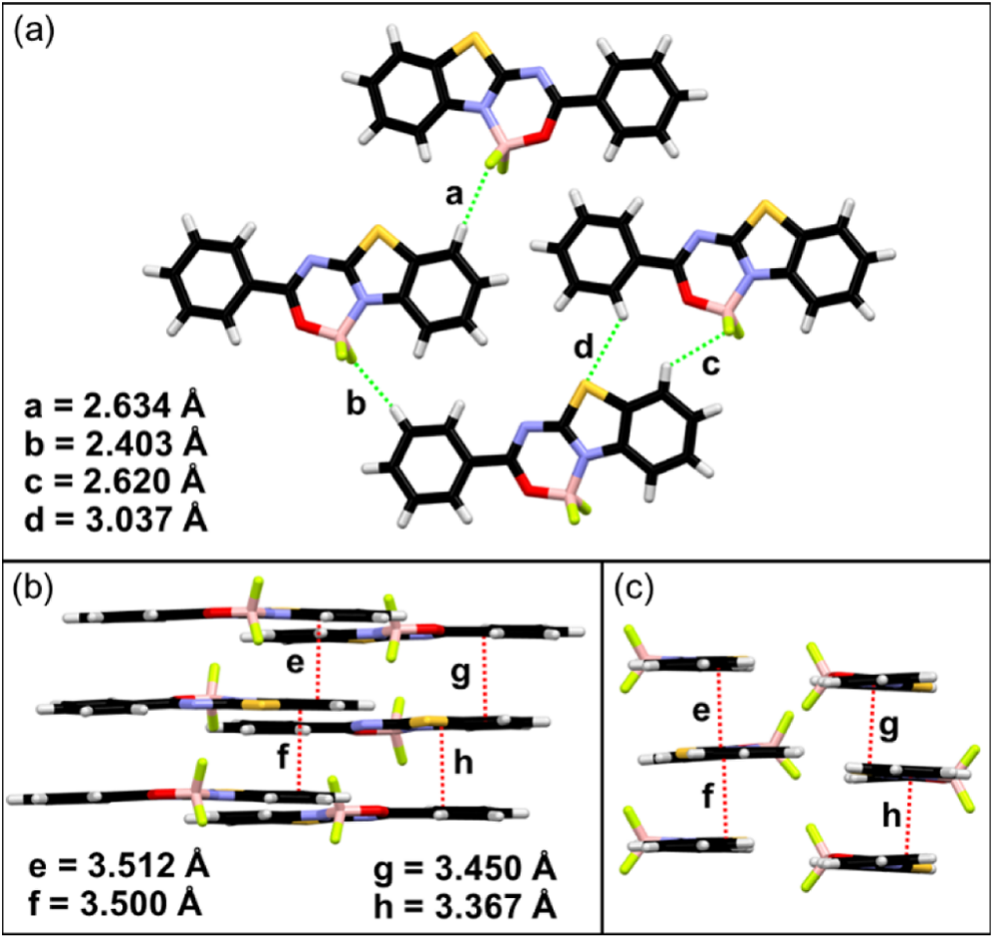
Intermolecular interactions in the crystal structure of dye **2**: CH···F and CH···S hydrogen bonds (a); π···π/*n*···π interactions, front view (b) and side view (c).

Compound **3** crystallizes in a monoclinic crystal system (Table S3 in the SI). The unit cell comprises 8 molecules, four of which are oriented parallel to each other and perpendicular to the other four molecules (Figure S7 in the SI). Coplanar molecules are bonded by π···π/*n*···π interactions (*e* = 3.398 Å and *f* = 3.448 Å, Figure 4b,c), forming columns. On the other hand, perpendicularly oriented molecules interact via various CH···F hydrogen bonds (*a* = 2.587 Å, *b* = 2.416 Å, *c* = 2.461 Å), as well as weak Se···F halogen-type bond interactions (*d* = 3.115 Å, Figure 4a).

**Figure 4:**
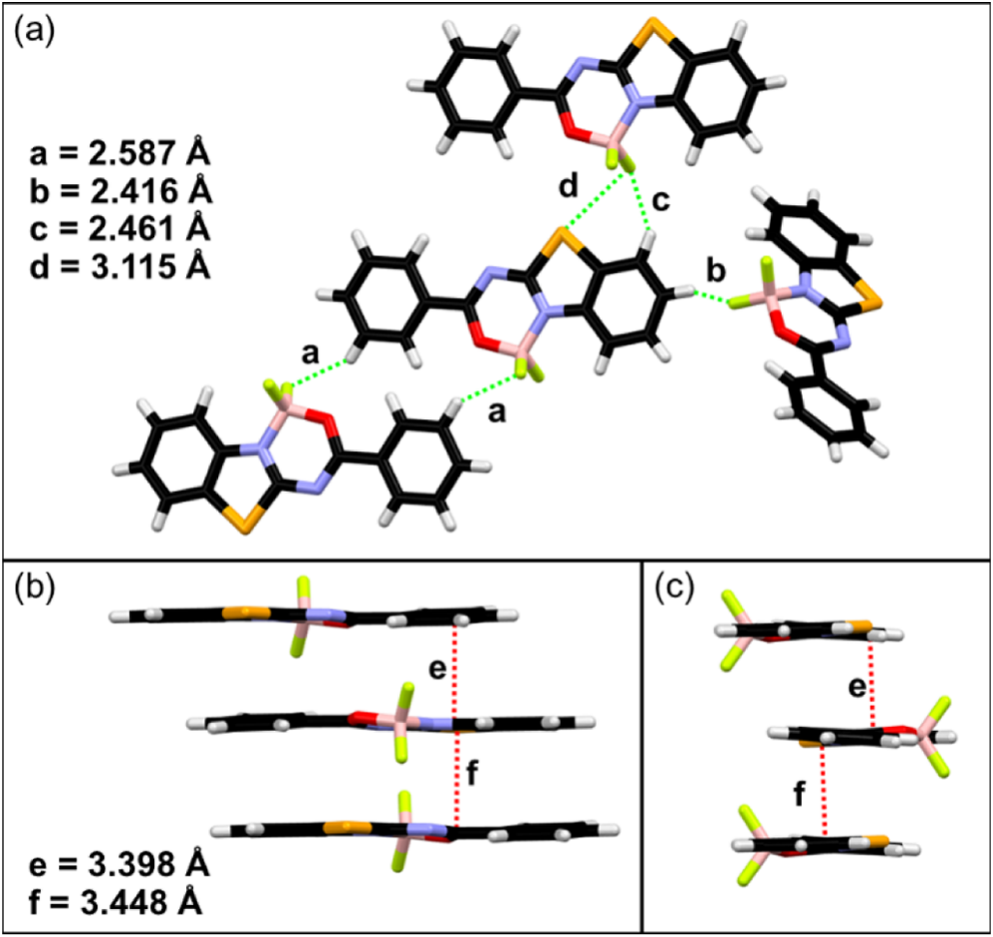
Intermolecular interactions in the crystal structure of dye **3**: CH···F hydrogen and Se···F halogen bond interactions (a); π···π/*n*···π interactions, front view (b), and side view (c).

Benzotellurazole-containing boron complex **4** crystallizes in an orthorhombic crystal system (Table S4 in the SI). The unit cell comprises 4 molecules (Figure S8 in the SI).

The molecular packing formed mainly with the participation of tellurium and fluorine atoms: Te···F (*b* = 3.379 Å) halogen bond, Te···π (*c* = 3.486 Å) interaction, CH···F (*a* = 2.419 Å, Figure 5a) hydrogen bond, and F···π (*d* = 3.133 Å, *e* = 3.131 Å, *f* = 3.059 Å, Figure 5b) interactions, supported by some weak π···π stacking (*g* = 3.387 Å, Figure 5b).

**Figure 5:**
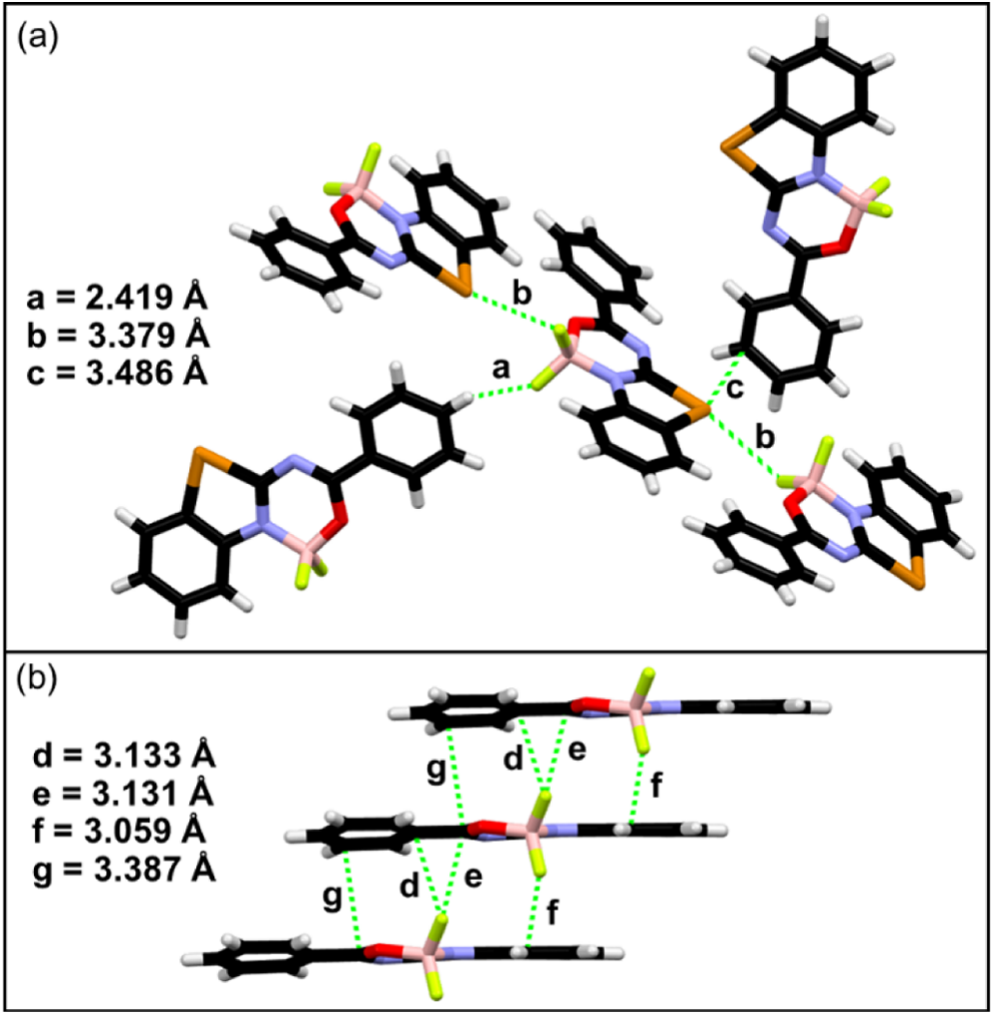
Intermolecular interactions in the crystal structure of dye **4**: CH···F hydrogen and Te···F halogen bond interactions and Te···π interactions (a); π···π/*n*···π interactions (b).

### Quantum Chemical Calculations

In order to shed light on the optical properties of synthesized dyes, quantum chemical calculations were performed. Molecules were optimized by the B3LYP method with the 6–31G­(d) basis set and additional LANL2DZ basis set was employed for Te atom. In the optimized structures, the C-X-C angle (X = chalcogen) in the chalcogenazole ring decreases with the increase of the chalcogen atom size: 106.0, 89.9, 86.0, and 79.6° for benzoxazole, benzothiazole, benzoselenazole, and benzotellurazole derivatives, respectively. This correlates well with the corresponding data from X-ray analysis (Table S5 in the SI).

The computations reveal that the phenyl ring is almost coplanar with a heterocyclic unit with torsion angles of 0.02, 1.06, 0.92, and 0.00° for dyes **1**, **2**, **3**, and **4**, respectively. This ensures efficient π-electron conjugation across the molecular framework. In accordance, the highest occupied and lowest unoccupied molecular orbitals (HOMO and LUMO) are evenly spread over the whole molecules. The energy difference between frontier orbitals systematically decreases with the increasing size of the chalcogen atom. Specifically, the HOMO–LUMO band gaps equal to 4.22 4.07, 4.00, and 3.84 eV for dyes **1**, **2**, **3**, and **4**, respectively (Figure 6). This trend is consistent with the bathochromic shift of the absorption bands observed in the experimental spectra and ionization potential order derived from cyclic voltammetry measurements, as demonstrated in the following paragraphs.

**Figure 6:**
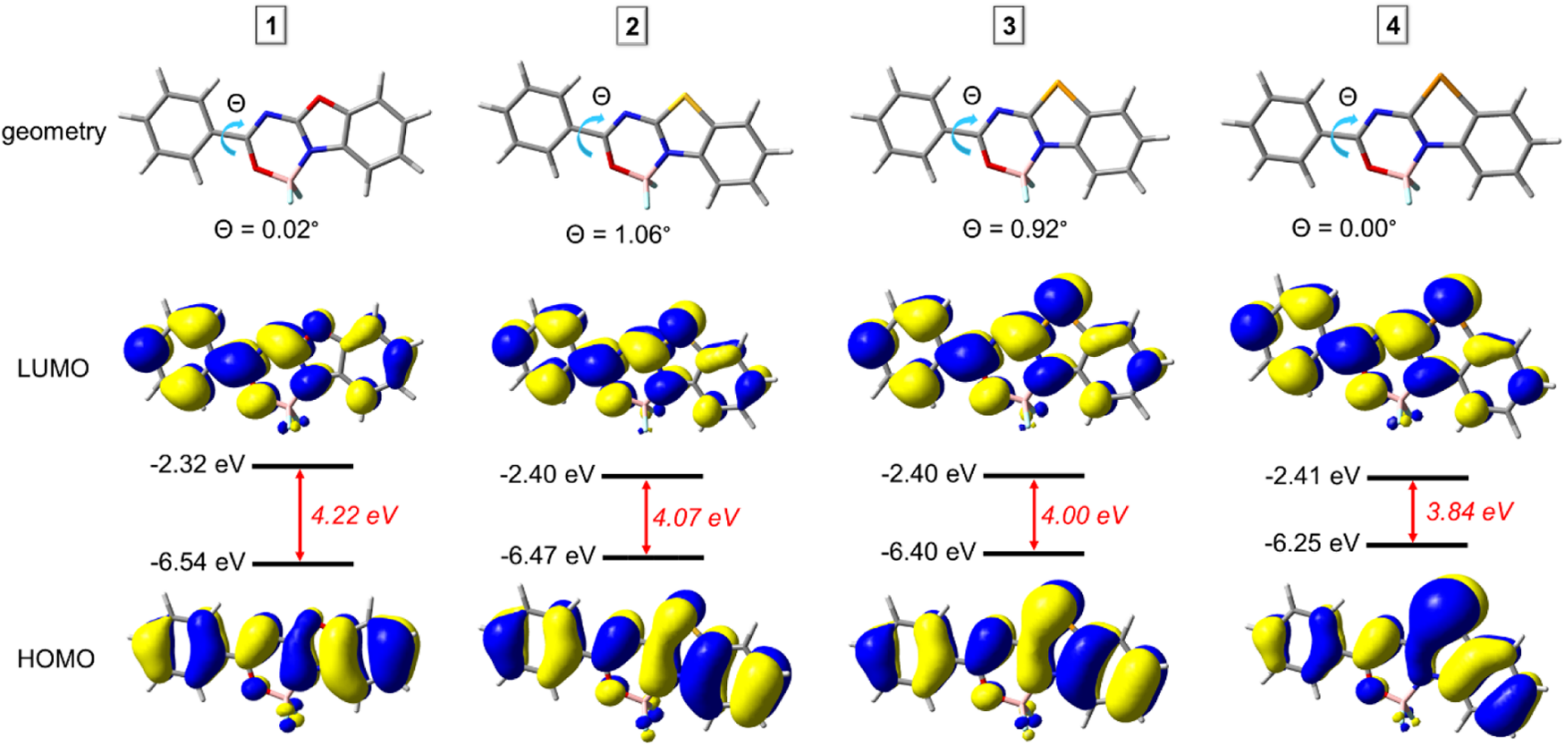
Optimized ground-state structures, HOMO, and LUMO for compounds **1–4**.

The narrowing of the HOMO–LUMO band gap mostly results from the elevation of HOMO. Specifically, HOMO increases from −6.54 eV for benzoxazole derivative **1** to −6.25 eV for benzotellurazole analogue **4**. The observed effect originates from the decreasing polarization of the carbon-chalcogen bond in series O → Te. This is confirmed by the calculated Hirshfeld atomic charges. In particular, the atomic partial charge of the two carbon atoms bonded to the chalcogen atom systematically decreases with the decrease of the electronegativity of the heavier chalcogen atoms (Table S7, in the SI). In contrast, the LUMO energy remains nearly constant at −2.40 eV for S-, Se-, and Te-containing systems and only slightly deviates for benzoxazole derivative **1** (−2.32 eV).

In order to understand the role of the chalcogen atom on the photophysical properties of boron complexes **1–4**, energies of singlet and triplet states as well as spin–orbit coupling elements (SOCME) were calculated using the PBE0 method with the ZORA-DEF2-TZVP basis set and RI-SOMF(1×) function to accelerate the SOC integrals in Orca 5.0. For Te, an additional basis set, SARC-ZORA-TZVP, was implemented. According to calculation results, it is clear that the values of SOCME between first singlet (S_1_) and triplet (T_1_) excited states are low for benzoxazole- and benzothiazole-based dyes (0.49 and 0.40 cm^–1^, respectively). Meanwhile, it increases significantly for the benzoselenazole analogue **3** (1.86 cm^–1^) (Table S8 in the SI), indicating a possibility of triplet state involvement in dye photophysics. Interestingly, for dye **4** SOC between the aforementioned state is diminished to 0.64 cm^–1^, however, the coupling between the second triplet (T_2_) and the S_1_ states is particularly high, with a value of 539.44 cm^–1^. Additionally, the calculated energy gap between S_1_ and T_2_ is lower (0.41 eV, Table S9 in the SI) compared to that between S_1_ and T_1_ (0.89 eV). These data suggest that the de-excitation of molecule **4** includes S_1_ → T_2_, T_2_ → T_1_, and T_1_ → S_0_ transitions.

### Electrochemical Properties

The electrochemical properties of boron difluoride complexes **1–4** were investigated by cyclic voltammetry (CV) (Figures S9–S12 and Table S10 in the SI). The values of ionization potentials (IPs) and electron affinities (EAs) were estimated using the following equations: IP = *E*_ox_^onset^ + 4.8 and EA = *E*_red_^onset^ + 4.8, where *E*_ox_^onset^ and *E*_red_^onset^ are the onset oxidation and the onset reduction potentials, respectively. The values of IPs decrease in the line of benzoxazole → benzothiazole → benzoselenazole → benzotellurazole derivatives: 6.46 6.39, 6.32, and 5.64 eV for dyes **1**, **2**, **3**, and **4**, respectively, consistent with the increasing of the HOMO energy level. In contrast, the values of EAs are of similar magnitude: 3.13, 3.15, 3.19, and 3.19 eV for dyes **1**, **2**, **3**, and **4**, respectively, indicating no significant change in the LUMO energy level of these dyes.

### Photophysical Properties of the Solutions and of the Solid Samples

First, we investigated the photophysical properties of the solutions of dyes **1–4** in toluene (Figure S13 and Table S11 in the SI). Compounds **1–4** absorb in the ultraviolet region. The solution of benzoxazole-containing dye **1** shows a broad main peak centered at 328 nm. Benzothiazole (**2**) and benzoselenazole (**3**) analogues exhibit bathochromically shifted absorption bands at 342 and 349 nm, with the shoulders at 355 and 362 nm, respectively. Meanwhile, the toluene solution of benzotellurazole derivative **4** demonstrates a much broader absorption band with the maximum at 380 nm and a hypsochromic shoulder at 362 nm (Figure S13a in the SI). According to the TD-DFT calculations, the main absorption peak of dyes **1–3** corresponds to the S_0_ → S_1_ transition, while transitions S_0_ → S_2_ and S_0_ → S_3_ have a negligible participation (Table S6 and Figures S14–S16 in the SI). In contrast, in the case of compound **4**, two transitions (S_0_ → S_1_ and S_0_ → S_3_) have relatively high oscillator strength (0.6170 and 0.2766, respectively, Table S6 in the SI), resulting in a much wider shape of the absorption spectra (Figure S17 in the SI). The comparison of the absorption spectra clearly indicates that the maximum of absorption bathochromically shifts in the lines of benzoxazole → benzothiazole → benzoselenazole → benzotellurazole derivatives.

Photoluminescence spectra of toluene solutions of dyes **1–3** have similar single-peak profiles. The maxima of emission intensities are located at 458, 445, and 456 nm for dyes **1**, **2**, and **3**, respectively (Figure S13b in the SI). In contrast, the toluene solution of benzotellurazole derivative **4** exhibits two emission peaks maximized at 464 and 529 nm. Photoluminescence quantum yields of the solutions of the investigated compounds in toluene are below 5% (Table S11 in the SI). To establish the influence of the triplet oxygen on the photoluminescence intensity, we recorded emission spectra before and after deoxygenation. The toluene solutions of benzoxazole, benzothiazole, and benzoselenazole-containing dyes **1–3** demonstrated no change after oxygen removal (Figure S18a–c in the SI). Meanwhile, the deoxygenation of the solution of benzotellurazole derivative **4** resulted in a significant increase of the emission intensity (Figure S18d in the SI). This observation partly indicates that the emission of tellurium-containing dye **4** occurs with the essential participation of triplet excited states. In more detail, the RTP nature of dye **4** was investigated in a polymer dispersion through temperature-dependent photophysical measurements and time-resolved emission spectroscopy (TRES), as shown below.

Due to the low emission efficiency of the investigated dyes in solutions, we studied their aggregation-induced emission ability. Thus, the emission spectra of the suspensions of dye **1** in tetrahydrofuran/water mixtures with different water contents (*f*_w_) were taken (Figures S21a,b in the SI). The value of the PLQY of dye **1** in tetrahydrofuran (*f*_w_ = 0%) is less than 0.1% (Table S12 in the SI), which is even lower than that of the corresponding toluene solution. This is probably caused by the lower viscosity of tetrahydrofuran (compared to toluene), which promotes intramolecular rotation and thus nonradiative deactivation of the excited dye molecules. The samples with water fraction from 0 to 85% exhibit a very weak emission. Meanwhile, the emission intensity gradually increases for the highly aqueous suspensions (*f*_w_ = 90 and 95%). This observation clearly indicates the AIE properties of benzoxazole-containing dye **1**. Analogical properties were observed for benzothiazole derivative **2** (Figure S21a,b in the SI). In contrast, benzoselenazole and benzotellurazole analogues **3** and **4** do not exhibit the AIE behavior (Figure S22 in the SI), suggesting their low solid-state photoluminescent efficiency.

The measurements of the photoluminescence properties of the crystalline sample of dyes **1–4** confirm this observation. Dyes **1** and **2** exhibit highly intensive SSE with intensity maxima at 454 and 457 nm, and PLQY of 45 and 48%, respectively (Figure S23 and Table S13 in the SI). The crystals of benzoselenazole analogue **3** are weakly emissive (PLQY = 4%), exhibiting a slightly bathochromically shifted emission band maximized at 472 nm. Meanwhile, the solid sample of benzotellurazole derivative **4** is nonemissive. The lifetimes of the excited state of the solid samples are in the short nanosecond range for benzoxazole and benzothiazole dyes **1** (2.46 ns) and **2** (2.64 ns) or subnanosecond range (0.28 ns) for benzoselenazole derivatives **3**, confirming the purely fluorescent nature. The high PLQYs and short nanosecond lifetimes of dyes **1** and **2** suggest their lasing ability. To gain more insight into such an ability, we investigated the photoluminescent properties of the molecular dispersions of the dyes in a polymeric matrix.

### Photophysical Properties of the Molecular Dispersions in the Polymer Matrix

Poly­(methyl methacrylate) (PMMA) was selected as the convenient polymer due to its transparency and excellent film formation ability. The dye/polymer ratio was 3:97. The films demonstrate absorption profiles similar to those of the corresponding toluene solutions. A systematic bathochromic shift in the main absorption maximum is observed across the benzoxazole → benzothiazole → benzoselenazole → benzotellurazole series with the absorption maxima located at 324, 342, 348, and 380 nm for dyes **1**, **2**, **3**, and **4**, respectively (Figure 7a and Table 1). The films of dyes **1–3** demonstrate broad emission bands maximized at 440–450 nm. The sample of benzoxazole-containing boron difluoride complex **1** shows a relatively high PLQY value of 22%, accompanied by a short lifetime of the excited state (1.01 ns). The PLQY value of the molecular dispersion of benzothiazole derivative **2** in PMMA is even much higher (48%), while the excited state lifetime is *ca.* 1.67 ns. The film containing benzoselenazole derivative **3** demonstrates a lower PLQY of 11% and a shorter lifetime of 0.61 ns. The short (sub)­nanosecond-range lifetimes of the films of the molecular dispersions of compounds **1–3** in PMMA indicate the prompt singlet nature of their photoluminescence. This is also confirmed by the comparison of their emission spectra recorded in an air atmosphere and in a vacuum (Figure S25 in the SI). They remain essentially unchanged, indicating that triplet states are not involved in photophysical processes.

**Figure 7:**
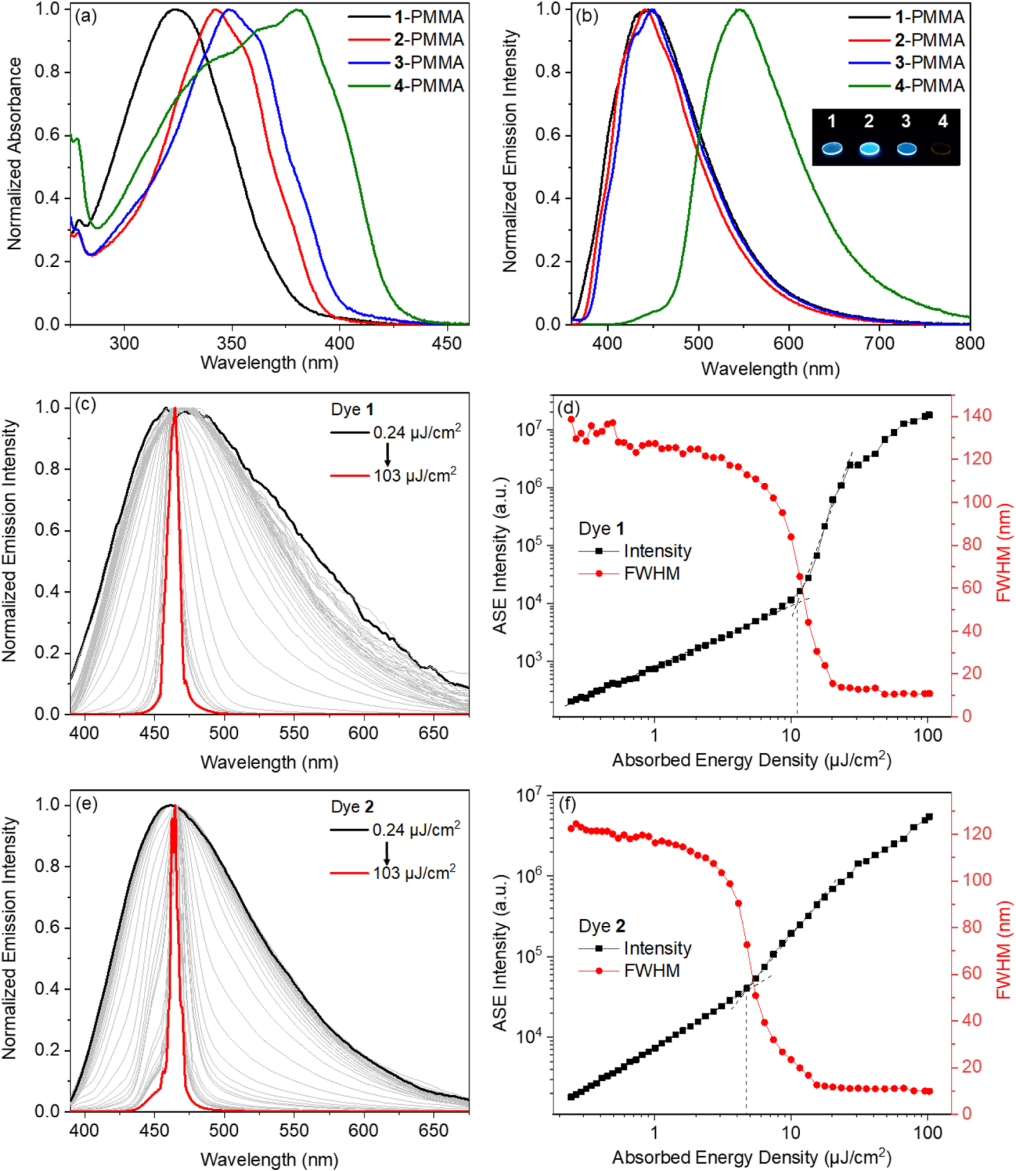
Absorption (a) and emission (b) spectra of the films of dye-doped PMMA containing compounds **1–4** (Insert: the photographs of the films of PMMA doped with dyes **1–4** taken under a UV light illumination). Normalized spectra of amplified spontaneous emission of dyes **1** (c) and **2** (e) molecularly dispersed in PMMA. Output intensity and fwhm values as a function of absorbed energy density for dyes **1** (d) and **2** (f).

**Table 1:** Photoluminescence Properties of Films of PMMA Doped with Compounds **1–4**

dye	λ_abs_, nm^a^	λ_PL_, nm^b^	fwhm_PL_, nm^c^	PLQY, %^d^	τ, ns^e^	*k*_r_, s^–1,^^f^	λ_ASE_, nm^g^	fwhm_ASE_, nm^h^	*E*_th‑ASE_, μJ/cm^2,^ ^i^
**1**	324	444	120	22	1.01	2.2·10^8^	465	10	11
**2**	342	440	105	45	1.67	2.7·10^8^	463	6.3	4.7
**3**	348	450	105	11	0.61	1.8·10^8^	–	–	–
**4**	380	470^j^/545^k^	121	0.5	9412	5.3·10^2^	–	–	–

a Wavelength of absorption maximum.

b Wavelength of photoluminescence maximum.

c Full width at half-maximum of the photoluminescence spectrum.

d Photoluminescence quantum yield measured in ambient atmosphere.

e Average excited-state lifetime.

f Rate constant of radiative deactivation.

g Wavelength of ASE maximum.

h Full width at half-maximum of the ASE spectrum.

i ASE threshold.

j Fluorescence detected as the high-energy weak intensive band.

k RTP detected as the low-energy strong, intensive band.

In sharp contrast, the film of benzotellurazole analogue **4** dispersed in PMMA shows strongly bathochromically shifted emission bands with a maximum at 545 nm (Figure 7b) with the low PLQY value of 0.5% (Table 1) and long lifetime of the excited state of *ca.* 9.4 μs. The comparison of the emission spectra, recorded in the air atmosphere and in a vacuum, demonstrates the quenching of the emission by oxygen (Figure S25d in the SI). All of these observations suggest that, in contrast to the films of compounds **1–3** dispersed in PMMA, the molecular dispersion of benzotellurazole-containing dye **4** in the same polymer exhibits RTP with a relatively fast lifetime of 9.4 μs (Table 1). Typically, phosphorescent OLED emitters containing iridium or platinum atoms are characterized by such fast lifetimes.^52,53^ Organic RTP emitters are primarily characterized by emission lifetimes in the microsecond range.^54^ The presence of heavy atoms causes a shortening of emission lifetimes in organic RTP emitters.^55^ Therefore, we predict a similar effect of tellurium on the RTP lifetime of dye **4**. We also investigated the photophysical properties of the films of PMMA doped with lower 1 and 2% dye contents. The emission spectra of these films are almost identical to those of the film with 3% of dyes, while the value of PLQY slightly decreases with the increasing dye concentration (Figure S27 and Table S14 in the SI). To investigate the nature of the microsecond luminescence of compound **4**, we additionally performed temperature-dependent photophysical measurements for the films of 1% solid solution in Zeonex. Zeonex matrix was selected to exclude any hypothetical influence of the polymer functional groups on the photophysical process. The photoluminescence intensity (Figure 8a,b), as well as lifetime values (Figure 8c and Table S15 in the SI), of this film gradually decrease in the temperature range from 100 to 300 K. This observation clearly confirms that the emission is the phosphorescence. To validate the statements above, we also recorded the time-resolved emission spectra of dye **4** dispersed in Zeonex (Figure 8d). The TRES experiment enabled us to distinguish between the fluorescence and RTP spectra of compound **4**. The intensity of the high-energy band (fluorescence) at 470 nm drops significantly within approximately 15 ns, whereas the intensity of the low-energy band (RTP) at 545 nm decreases slowly within 1 μs. The long-lived emission cannot be attributed to delayed fluorescence, as its spectrum appears at distinctly different wavelengths. Thus, the large singlet–triplet energy gap (∼0.51 eV) should prevent the reverse intersystem crossing needed for thermally activated delayed fluorescence. This interpretation is consistent with the observed decrease in emission intensity upon successive increases in temperature (Figure 8a,b).

**Figure 8:**
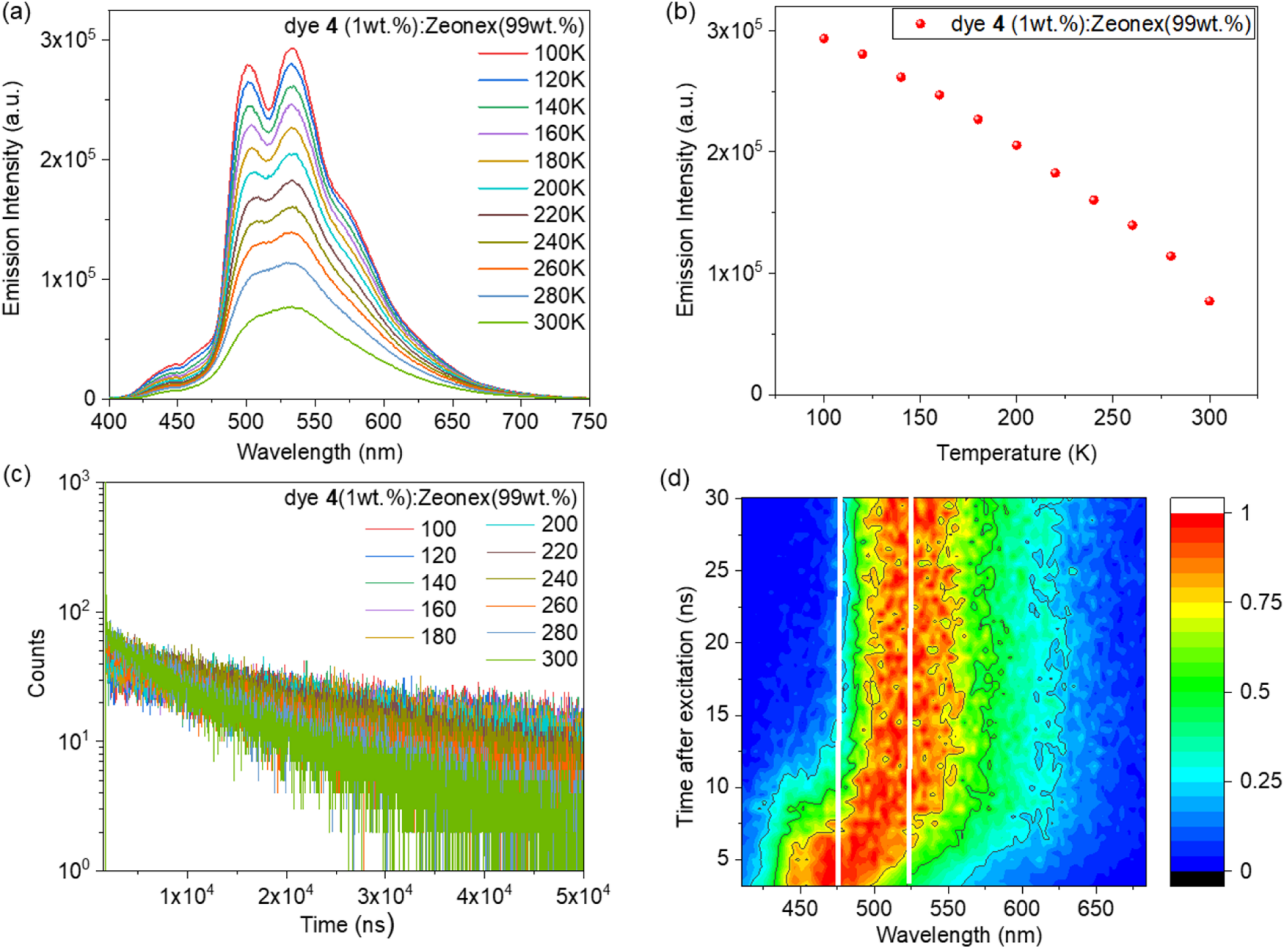
Photoluminescence spectra (a), emission intensity (b), and photoluminescence decay curves (c) of the films of the molecular dispersion of dye **4** (1 wt %) in Zeonex at different temperatures. Time-resolved emission spectra of the films of the molecular dispersion of dye **4** (1 wt %) in Zeonex (d).

As mentioned above, the emission spectra of the films of the dye-doped PMMA are broad: the values of fwhm are larger than 100 nm (Table 1). On the other hand, materials that demonstrate high PLQY and short lifetime could exhibit lasing properties, narrowing the emission spectra and increasing the color purity.^56^ In practice, to reduce research time and costs, the amplified spontaneous emission (ASE) threshold is typically assessed in place of the lasing threshold for new gain materials.^57–59^ Therefore, for the investigation of the potential for the amplification of light, we studied the ASE ability of the dyes. To this end, we used a nitrogen laser (operating at a wavelength of 337 nm) as the excitation source. A dye-doped PMMA film with a dye concentration of 3% was spin-casted on a Silica substrate at 1500 rpm during 60 s. The measured layer thickness of *ca.* 500 nm allows single-mode propagation in the film and corresponds to an absorption of 30% at 337 nm for compounds **1** and **2**. In order to create a stripe-shaped excitation onto the organic thin film, the laser was first focused onto a slit with a cylindrical ultraviolet (UV) lens (focal length of 300 mm). The slit had an adjustable aperture in order to tune the length of the excitation area. The slit was then relay-imaged onto the sample with a 1:1 telescope composed of two identical 100 mm focal length UV-coated singlets (Thorlabs LA4380-UV-ML) as depicted in Figure 9a. The final stripe dimensions onto the sample were 10.4 mm in length and 180 μm in width, measured at 50% of the maximum (Figure 9). Imaging of the slit onto the sample – instead of directly setting the sample close to the slit – enables defining a perfectly clean transition between pumped and unpumped regions and eliminates Fresnel diffraction patterns.^60^ For spectrum collection, the silica substrate with the spin-cast organic thin film was first cleaved in order to allow for a clean interface with a directional ASE visible beam that is more efficiently collected. Spectrum measurement was made upon approaching a fiber-coupled spectrometer (Horiba iHR550) with a fiber core diameter of 400 μm in close vicinity to the cleaved facet. For data processing, all spectra were first normalized and smoothed in order to remove noise and obtain a reliable measurement of the full width at half-maximum. The ASE curves showing typical nonlinear increase of the ASE intensity *vs* pump energy density and shrinking of the full widths at half maxima (fwhm) are shown in Figure 7d,f for compounds **1** and **2**, respectively. The normalized spectral evolution for increasing pump powers is shown in Figure 7c,e. The threshold value for ASE was taken at the energy or peak power value where the variation in the slope of the integrated intensity was the highest. Low ASE thresholds of 11 and 4.7 μJ/cm^2^ are reported for compounds **1** and **2** (Figure 7d,f).

**Figure 9:**
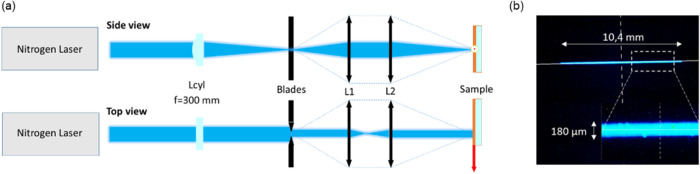
(a) Experimental setup for the ASE measurement (side and top view). (b) Image and dimensions of the pumping stripe.

The lower threshold obtained for the film of dye **2** dispersed in PMMA can be explained by its higher PLQY, which is also reflected in its stronger fluorescence at lower pump energy densities (Figure 7f). The gradual increase of the energy density of the nitrogen laser results in the emission spectra narrowing. The final ASE spectrum is characterized by a fwhm of 10 and 6.3 nm, more than 1 order of magnitude narrower than the fluorescence spectrum. The measured ASE maxima (λ_ASE_ = 465 and 463 nm) are shifted to the red compared to the PL spectra reported above (Table 1) because in the ASE setup configuration, the PL is collected after propagation along the stripe, making the self-absorption effect more significant. However, this effect is relatively mild here because the reabsorption is weak, as witnessed by the very small overlap between the absorption and fluorescence spectra for compounds **1** and **2** (Figure 7a,b). More specifically, the reabsorption coefficients are measured as only 1.07 and 0.75 cm^–1^ at λ_ASE_ for compounds **1** and **2**, respectively, which is much less than what is typically observed in organic materials.

Due to their low PLQY values, no ASE ability was observed for benzoselenazol and benzotellurazole derivatives **3** and **4**.

## Conclusions

We developed a synthetic pathway to a series of benzochalcogenazole-based boron difluoride complexes. The influence of the chalcogen atom on the electrochemical and photophysical properties is investigated. The dyes are weakly luminescent in solutions, but they demonstrate essential solid-state photoluminescence. Due to the high PLQY and short lifetime of the excited state, benzoxazole- and benzothiazole-containing compounds exhibit amplified spontaneous emission. Meanwhile, the increasing of the size of the chalcogen results in an increase of the phosphorescence ability of the dyes. Therefore, the benzotellurazole-based boron difluoride complex shows room temperature phosphorescence characterized by a short-microsecond lifetime of 9.4 μs. Overall, our work demonstrates the perceptivity of the elaboration of both lasing and phosphorescent dyes with a benzochalcogenazole-based boron difluoride scaffold. For further application of this class of dye in organic semiconducting laser diodes, new chalcogen-containing dyes are currently being investigated in our laboratory.

## Experimental Section

### General

All reagents and chemicals (at least analytical grade) were purchased from commercial sources (Thermo Scientific, TCI, Acros Organics, and Roth) and used without further purification. Synthesized compounds were purified via flash column chromatography on silica gel (Merck, 230–400 mesh). Reaction progress was controlled by thin layer chromatography (TLC), carried out on commercially available aluminum plates covered with silica gel (60 F254, Merck).

### Instrumental Methods

NMR spectra were recorded on a Varian Mercury 400 MHz (at 400 and 100 MHz for ^1^H and ^13^C NMR spectra, respectively), Varian V NMRS 500 MHz (at 500, 125, 470, and 95 MHz for ^1^H, ^13^C, ^19^F, and ^77^Se NMR spectra, respectively), or Varian V NMRS 600 MHz (at 600 and 150 MHz for ^1^H and ^13^C NMR spectra, respectively) spectrometers at room temperature using CDCl_3_ or DMSO-*d*_*6*_ as a solvent, and referenced externally to SiMe_4_. The multiplicities of the signals are indicated as “s”, “d’, “t”, or “m”, which stand for singlet, doublet, triplet, and multiplet, respectively. The data was evaluated by using MestReNova software.

High-resolution mass spectra (HRMS) were collected on a Synapt G2-S HDMS (Waters Inc.) mass spectrometer equipped with an atmospheric-pressure chemical ionization (APCI) ion source or electrospray ionization (ESI) ion source and quadrupole time-of-flight (q-TOF) mass analyzer. The instrument was controlled, and recorded data were processed using the MassLynx V4.1 software package (Waters Inc.). Spectra were collected in the ESI or APCI mode.

Single crystals of the investigated boron difluoride complexes were grown by slow evaporation of their solution in cyclohexane/dichloromethane (5:1) mixture for structure **1**, cyclohexane/dichloromethane (3:1) mixture for structure **2**, hexanes/dichloromethane (2:1) mixture for structure **3**, and chloroform/methanol (5:1) for structure **4** under ambient conditions. Single crystal X-ray diffraction measurements were carried out on an Agilent Supernova diffractometer at 100 K with monochromated Cu Kα radiation (1.54184 Å). The data reduction was made by using *CrysAlisPRO* software.^61^ The structures were solved by direct methods and refined with the olex2.refine^62^ refinement package using Gauss–Newton minimization. All non-hydrogen atoms were refined as anisotropic, while hydrogen atoms were placed in calculated positions and refined in riding mode. Compound **2** contains two symmetry-independent molecules in the unit cell. Crystallographic data of compounds **1–4** have been deposited with the Cambridge Crystallographic Data Centre (CCDC) and can be obtained, free of charge, from CCDC via https://www.ccdc.cam.ac.uk/structures/.

UV–visible (UV–vis) spectra in solutions were recorded on a Shimadzu UV-3600i Plus spectrophotometer at room temperature. Steady-state photoluminescence emission spectra were performed at room temperature on an Edinburgh Instruments Spectrophotometer (FS5, Edinburgh, U.K.) with a xenon lamp as the light source. The absolute photoluminescence quantum yields were measured with a calibrated SC-30 Integrating Sphere and were excited at the appropriate absorption wavelength in each case. Photoluminescence decay curves of the solutions and of the solid samples were recorded using a time-correlated single photon counting technique utilizing the PicoQuant PDL 820 ps pulsed diode laser as an excitation source (λ_ex_ = 340 nm for dyes **1–3** and λ_ex_ = 374 nm for dye **4**).

The ASE measurements were carried out using a Nitrogen laser at 337 nm as the pump source (Stanford Research Systems, NL100, 3.5 ns pulse duration).

### Synthesis

#### *N*-((2-Hydroxyphenyl)­carbamothioyl)­benzamide (**8**)

A mixture of benzoyl chloride (**5**, 0.60 mL, 5.17 mmol) and potassium thiocyanate (518 mg, 5.33 mmol) in dry acetone (10 mL) was refluxed for 1h. 2-Aminophenol (**7**, 564 mg, 5.17 mmol) was added, and the refluxing was continued for 1h more. After the mixture cooled, the solvent was evaporated in vacuo, and then, water (15 mL) was added. The mixture was stirred for 10 min, and the precipitate was filtered, dried, and recrystallized from ethanol to give the product **8** (1.085 g, 3.98 mmol, 77%) as a white solid. ^1^H NMR (600 MHz, DMSO-*d*_*6*_): δ = 12.96 (1H, s, NH), 11.47 (1H, s, NH), 10.24 (1H, s, OH), 8.55 (1H, dd, *J* = 8.1 Hz, *J* = 1.3 Hz, Ar–H), 7.98 (2H, dd, *J* = 8.2 Hz, *J* = 1.2 Hz, Ar–H), 7.66 (1H, tt, *J* = 7.5 Hz, *J* = 1.3 Hz, Ar–H), 7.54 (2H, dd, *J* = 8.1 Hz, *J* = 7.5 Hz, Ar–H), 7.08 (1H, ddd, *J* = 8.2 Hz, *J* = 7.7 Hz, *J* = 1.6 Hz, Ar–H), 6.96 (1H, dd, *J* = 8.1 Hz, *J* = 1.3 Hz, Ar–H), 6.85 (1H, ddd, *J* = 8.2 Hz, *J* = 7.7 Hz, *J* = 1.3 Hz, Ar–H) ppm; ^13^C NMR (150 MHz, DMSO-*d*_*6*_): δ = 177.49, 168.26, 148.92, 133.08, 132.16, 128.68 (2C), 128.43 (2C), 126.46, 125.96, 123.19, 118.32, 115.12 ppm. HRMS (APCI) calcd for C_14_H_13_N_2_O_2_S [M + H]^+^: 273.0698, found: 273.0694.

#### *N*-(Benzo­[*d*]­oxazol-2-yl)­benzamide (**10**)

*N*,*N*-Diethyl-*N*-{[(methoxycarbonyl)­amino]­sulfonyl}-ethanaminium inner salt (**9**, 529 mg, 2.22 mmol) was added to a suspension of compound **8** (550 mg, 2.02 mmol) in dry toluene (50 mL). The reaction mixture was stirred for 2h at 80 °C and concentrated. The product was purified by flash chromatography on silica gel with hexanes/dichloromethane (80:20 to 20:80, v/v) mixtures as an eluent to give product **10** (429 mg, 1.80 mmol, 89%) as a white solid. ^1^H NMR (500 MHz, DMSO-*d*_*6*_): δ = 12.04 (1H, br s, N–H), 7.95–8.21 (2H, m, Ar–H), 7.45–7.77 (5H, m, Ar–H), 7.27–7.38 (2H, m, Ar–H) ppm; ^13^C NMR (125 MHz, DMSO-*d*_*6*_): δ = 164.91, 155.46, 147.91, 140.64, 132.73, 128.54 (2C), 128.37, 124.65, 123.88 (3C), 118.46, 110.19 ppm. HRMS (ESI-TOF) calcd for C_14_H_11_N_2_O_2_ [M + H]^+^: 239.0821, found: 239.0818.

#### *N*-(Benzo­[*d*]­thiazol-2-yl)­benzamide (**13**)

A mixture of benzoyl chloride (**5**, 0.50 mL, 4.31 mmol) and potassium thiocyanate (431 mg, 4.44 mmol) in dry acetone (10 mL) was refluxed for 1h. A solution of 2-aminobenzenethiol (**11**, 539 mg, 4.31 mmol) in acetone (2 mL) was added, and the refluxing was continued for 1h more. After the mixture was cooled, the solvent was evaporated in vacuo, and then water (15 mL) was added. The mixture was stirred for 10 min, and the precipitate was filtered, dried, and recrystallized from ethanol to give product **13** (755 mg, 2.97 mmol, 69%) as a yellowish solid. ^1^H NMR (400 MHz, DMSO-*d*_*6*_): δ = 12.88 (1H, br s, NH), 8.16 (2H, dd, *J* = 7.6 Hz, *J* = 1.4 Hz, Ar–H), 8.01 (1H, d, *J* = 7.7 Hz, Ar–H), 7.79 (1H, d, *J* = 8.0 Hz, Ar–H), 7.65 (1H, t, *J* = 7.3 Hz, Ar–H), 7.56 (2H, dd, *J* = 7.7 Hz, *J* = 7.3 Hz, Ar–H), 7.46 (1H, ddd, *J* = 8.2 Hz, *J* = 7.2 Hz, *J* = 1.2 Hz, Ar–H), 7.33 (1H, ddd, *J* = 8.2 Hz, *J* = 7.6 Hz, *J* = 1.0 Hz, Ar–H) ppm; ^13^C NMR (100 MHz, DMSO-*d*_*6*_): δ = 166.00, 158.90, 148.26, 132.80, 131.94, 131.48, 128.59 (2C), 128.30 (2C), 126.13, 123.64, 121.68, 120.28 ppm. HRMS (ESI-TOF) calcd for C_14_H_11_N_2_OS [M + H]^+^: 255.0592, found: 255.0599.

#### *N*-(Benzo­[*d*]­[1,3]­selenazol-2-yl)­benzamide **(16**)

A mixture of benzoyl chloride (**5**, 0.50 mL, 4.31 mmol) and potassium thiocyanate (431 mg, 4.44 mmol) in dry acetone (10 mL) was refluxed for 1h. A solution of 2,2′-diselanediyldianiline (**14**, 737 g, 2.15 mmol) in acetone (2 mL) was added, and the refluxing was continued for 1h more. After cooling, the solvent was evaporated in vacuo, and then water (15 mL) was added. The mixture was stirred for 10 min, and the precipitate was filtered, dried, and purified by flash column chromatography on silica gel (hexanes/DCM = from 4:1 to 1:2, v/v) to give product **16** (1.015 g, 3.37 mmol, 78%) as a yellowish solid. ^1^H NMR (500 MHz, CDCl_3_): δ = 10.05 (1H, br s, NH), 7.96 (2H, dd, *J* = 8.4 Hz, *J* = 1.2 Hz, Ar–H), 7.89 (1H, m, Ar–H), 7.52 (1H, dd, *J* = 7.5 Hz, *J* = 7.4 Hz, Ar–H), 7.37 (2H, dd, *J* = 8.1 Hz, *J* = 7.5 Hz, Ar–H), 7.34 (1H, m, Ar–H), 7.19–7.27 (2H, m, Ar–H) ppm; ^13^C NMR (125 MHz, CDCl_3_): δ = 166.63, 161.00, 148.51, 134.30, 133.05, 132.05, 128.92 (2C), 127.90 (2C), 126.15, 124.42, 124.02, 121.78 ppm; ^77^Se NMR (95 MHz, CDCl_3_): δ = 593.13 ppm. HRMS (ESI-TOF) calcd for C_14_H_9_N_2_OSe [M – H]^−^: 300.9880, found: 300.9879.

#### 2-Aminobenzenetellurol (**17**)

Compound **17** was synthesized according to the modified literature procedure.^63^ The mixture of 2-iodoaniline (1.32 g, 6.03 mmol), copper­(II) oxide (48 mg, 0.60 mmol), potassium hydroxide (675 mg, 12.05 mmol), and tellurium (1.54 g, 12.05 mmol) in dry DMSO (12 mL) was stirred at 90 °C for 24. Then, the solvent was evaporated in vacuo, and the product was purified by flash column chromatography on silica gel with hexanes/dichloromethane (100:0 to 80:20, v/v) mixtures as an eluent to give product **17** (574 mg, 2.60 mmol, 43%) as a gray solid. ^1^H NMR (400 MHz, CDCl_3_): δ = 7.66 (1H, dd, *J* = 7.6 Hz, *J* = 1.6 Hz, Ar–H), 7.14 (2H, ddd, *J* = 8.6 Hz, *J* = 7.6 Hz, *J* = 1.6 Hz, Ar–H), 6.75 (1H, dd, *J* = 8.0 Hz, *J* = 1.3 Hz, Ar–H), 6.59 (1H, ddd, *J* = 8.6 Hz, *J* = 7.4 Hz, *J* = 1.3 Hz, Ar–H) ppm; ^13^C NMR (100 MHz, CDCl_3_): δ = 149.42, 140.61, 130.47, 119.68, 114.52, 100.56 ppm.

#### *N*-[(2-Hydrotellurophenyl)­carbamothioyl]­benzamide (**18**)

A mixture of benzoyl chloride (**5**, 0.25 mL, 2.15 mmol) and potassium thiocyanate (216 mg, 2.22 mmol) in dry acetone (10 mL) was refluxed for 1h. A solution of 2-aminobenzenetellurol (**17**, 475 mg, 2.15 mmol) in acetone (2 mL) was added, and the refluxing was continued for 1h more. After the mixture cooled, the solvent was evaporated in vacuo, and then water (10 mL) was added. The mixture was extracted with dichloromethane (3 × 20 mL). The combined organic phases were dried over anhydrous Na_2_SO_4_ and filtered. The solvent was removed under reduced pressure, and the crude product was purified by flash column chromatography on silica gel with a hexanes/dichloromethane (50:50 to 0:100, v/v) mixture as an eluent to give product **18** (523 mg, 1.36 mmol, 63%) as an orange solid. ^1^H NMR (600 MHz, DMSO-*d*_*6*_): δ = 12.53 (1H, s, NH), 11.70 (1H, s, NH), 7.99 (2H, dd, *J* = 8.2 Hz, *J* = 1.2 Hz, Ar–H), 7.65–7.68 (2H, m, Ar–H), 7.53 (2H, dd, *J* = 8.3 Hz, *J* = 7.3 Hz, Ar–H), 7.42 (1H, dd, *J* = 8.1 Hz, *J* = 1.4 Hz, Ar–H), 7.36 (1H, ddd, *J* = 8.8 Hz, *J* = 7.4 Hz, *J* = 1.5 Hz, Ar–H), 7.08 (1H, ddd, *J* = 8.8 Hz, *J* = 7.6 Hz, *J* = 1.4 Hz, Ar–H) ppm; ^13^C NMR (100 MHz, DMSO-*d*_*6*_): δ = 180.52, 168.28, 141.30, 139.42, 133.21, 131.91, 128.80 (2C), 128.69, 128.41 (2C), 127.84, 126.68, 121.66 ppm. HRMS (ESI-TOF) calcd for C_14_H_11_N_2_O_2_Te [M – H]^−^: 384.9654, found: 384.9652.

#### *N*-(Benzo­[*d*]­[1,3]­tellurazol-2-yl)­benzamide (**19**)

*N*,*N*-Diethyl-*N*-{[(methoxycarbonyl)­amino]­sulfonyl}-ethanaminium inner salt (**9**, 348 mg, 1.46 mmol) was added to a suspension of compound **18** (510 mg, 1.33 mmol) in dry toluene (40 mL). The reaction mixture was stirred for 2h at 80 °C and concentrated. The product was purified by flash chromatography on silica gel with hexanes/dichloromethane (80:20 to 20:80, v/v) mixtures as an eluent to give product **19** (205 mg, 0.59 mmol, 44%) as a yellow solid. ^1^H NMR (400 MHz, CDCl_3_): δ = 8.02 (2H, d, *J* = 7.6 Hz, Ar–H), 7.89 (1H, d, *J* = 7.6 Hz, Ar–H), 7.45–7.51 (2H, m, Ar–H), 7.36 (2H, dd, *J* = 7.8 Hz, *J* = 7.6 Hz, Ar–H), 7.31 (1H, dd, *J* = 8.2 Hz, *J* = 7.3 Hz, Ar–H), 7.14 (1H, dd, *J* = 7.3 Hz, *J* = 7.6 Hz, Ar–H) ppm; ^13^C NMR (100 MHz, CDCl_3_): δ = 170.70, 163.37, 148.02, 133.49, 132.79, 131.14, 128.67 (2C), 128.13 (2C), 127.72, 126.95, 123.86, 120.48 ppm. HRMS (ESI-TOF) calcd for C_14_H_11_N_2_OTe [M + H]^+^: 352.9934, found: 352.9930.

#### General Procedure A: Synthesis of Complexes **1**–**4**

Distilled *N*,*N*-diisopropylethylamine (1.79–5.85 mL, 10.29–33.58 mmol, 20 equiv) was added to a suspension of amide **10**, **13**, **16**, or **19** (0.51–1.80 mmol, 1 equiv) in dry DCM (25 mL) under an argon atmosphere. The mixture was stirred for 15 min at room temperature. Then, BF_3_·Et_2_O (0.34–2.07 mL, 5.15–16.79 mmol, 10 equiv) was added. The reaction mixture was stirred for 24 h at room temperature and then washed with a saturated aqueous solution of NaHCO_3_ (30 mL). The organic phase was dried in anhydrous Na_2_SO_4_ and filtered. The solvent was removed under reduced pressure, and the crude product was purified by flash column chromatography on silica gel.

#### 1,1-Difluoro-3-phenyl-1*H*-1λ^4^,10λ^4^-benzo­[4,5]­oxazolo­[3,2-*c*]­[1,3,5,2]­oxadiazaborinine (**1**)

Product **1** was obtained as a white crystalline solid in 70% yield (337 mg) using general procedure A from compound **10** (400 mg, 1.68 mmol), *N*,*N*-diisopropylethylamine (5.85 mL, 33.58 mmol), and BF_3_·Et_2_O (2.07 mL, 16.79 mmol). Flash column chromatography purification was performed with hexanes/dichloromethane mixtures (8:1 to 2:1, v/v) as an eluent. ^1^H NMR (500 MHz, CDCl_3_): δ = 8.43 (2H, dd, *J* = 8.5 Hz, *J* = 1.2 Hz, Ar–H), 7.76 (1H, d, *J* = 7.6 Hz, Ar–H), 7.69 (1H, ddd, *J* = 7.6 Hz, *J* = 7.4 Hz, *J* = 1.1 Hz, Ar–H), 7.62 (1H, dd, *J* = 7.4 Hz, *J* = 1.1 Hz, Ar–H), 7.46–7.56 (4H, m, Ar–H) ppm; ^13^C NMR (125 MHz, CDCl_3_): δ = 173.82, 163.04, 146.13, 135.09, 130.72, 130.64 (2C), 129.58, 128.75 (2C), 126.85, 126.54, 115.36, 111.55 ppm; ^19^F NMR (470 MHz, CDCl_3_): δ = – 135.96 (2F, m, BF_2_) ppm. HRMS (APCI) calcd for C_14_H_10_BN_2_O_2_F_2_ [M + H]^+^: 287.0803, found: 287.0802. HRMS (ESI-TOF) calcd for C_14_H_10_BN_2_O_2_F_2_ [M + H]^+^: 287.0803, found: 287.0804.

#### 1,1-Difluoro-3-phenyl-1*H*-1λ^4^,10λ^4^-benzo­[4,5]­thiazolo­[3,2-*c*]­[1,3,5,2]­oxadiazaborinine (**2**)

Product **2** was obtained as a white crystalline solid in 72% yield (360 mg) using general procedure A from compound **13** (420 mg, 1.65 mmol), *N*,*N*-diisopropylethylamine (5.75 mL, 33.03 mmol), and BF_3_·Et_2_O (2.04 mL, 16.52 mmol). Flash column chromatography purification was performed with hexanes/dichloromethane mixtures (4:1 to 1:1.5, v/v) as an eluent. ^1^H NMR (500 MHz, CDCl_3_): δ = 8.40 (2H, d, *J* = 7.6 Hz, Ar–H), 8.08 (1H, d, *J* = 8.2 Hz, Ar–H), 7.81 (1H, d, *J* = 8.1 Hz, Ar–H), 7.65 (1H, t, *J* = 7.4 Hz, Ar–H), 7.61 (1H, dd, *J* = 8.2 Hz, *J* = 7.5 Hz, Ar–H), 7.52 (2H, dd, *J* = 7.6 Hz, *J* = 7.4 Hz, Ar–H), 7.49 (1H, dd, *J* = 8.1 Hz, *J* = 7.5 Hz, Ar–H) ppm; ^13^C NMR (125 MHz, CDCl_3_): δ = 174.00, 168.45, 140.03, 134.44, 130.94, 130.44 (2C), 128.65 (2C), 128.40, 127.17, 126.51, 122.14, 118.64 ppm; ^19^F NMR (470 MHz, CDCl_3_): δ = −135.80 (2F, m, BF_2_) ppm. HRMS (ESI-TOF) calcd for C_14_H_10_BN_2_OF_2_S [M + H]^+^: 303.0575, found: 303.0579.

#### 1,1-Difluoro-3-phenyl-1*H*-1λ^4^,10λ^4^-benzo­[4,5]­[1,3]­selenazolo­[3,2-*c*]­[1,3,5,2]­oxadiazaborinine (**3**)

Product **3** was obtained as a yellowish crystalline solid in 72% yield (416 mg) using general procedure A from compound **16** (503 mg, 1.54 mmol), *N*,*N*-diisopropylethylamine (5.39 mL, 30.93 mmol), and BF_3_·Et_2_O (1.91 mL, 15.47 mmol). Flash column chromatography purification was performed with hexanes/dichloromethane mixtures (8:1 to 2:1, v/v) as an eluent. ^1^H NMR (500 MHz, CDCl_3_): δ = 8.40 (2H, dd, *J* = 8.4 Hz, *J* = 1.2 Hz, Ar–H), 8.14 (1H, d, *J* = 8.4 Hz, Ar–H), 7.82 (1H, d, *J* = 8.0 Hz, Ar–H), 7.65 (1H, dd, *J* = 7.6 Hz, *J* = 7.4 Hz, Ar–H), 7.58 (1H, ddd, *J* = 8.4 Hz, *J* = 7.8 Hz, *J* = 1.1 Hz, Ar–H), 7.52 (2H, dd, *J* = 8.0 Hz, *J* = 7.5 Hz, Ar–H), 7.42 (1H, ddd, *J* = 8.0 Hz, *J* = 7.6 Hz, *J* = 1.0 Hz, Ar–H) ppm; ^13^C­{H} NMR (125 MHz, CDCl_3_): δ = 179.14, 166.89, 141.54, 134.47, 130.83, 130.54 (2C), 129.22, 128.67 (2C), 128.19, 126.44, 125.05, 120.35 (t, *J*_C–F_ = 2.7 Hz) ppm; ^19^F NMR (470 MHz, CDCl_3_): δ = – 135.28 (2F, m, BF_2_) ppm; ^77^Se NMR (95 MHz, CDCl_3_): δ = 542.40 ppm. HRMS (ESI-TOF) calcd for C_14_H_10_BN_2_OF_2_Se [M + H]^+^: 351.0019, found: 351.0023.

#### 1,1-Difluoro-3-phenyl-1*H*-1λ^4^,10λ^4^-benzo­[4,5]­[1,3]­tellurazolo­[3,2-*c*]­[1,3,5,2]­oxadiazaborinine (**4**)

Product **4** was obtained as a yellowish amorphous solid in 45% yield (93 mg) by using general procedure A from compound **19** (180 mg, 0.51 mmol), *N*,*N*-diisopropylethylamine (1.79 mL, 10.29 mmol), and BF_3_·Et_2_O (0.63 mL, 5.15 mmol). Flash column chromatography purification was performed with hexanes/dichloromethane mixtures (5:1 to 2:1, v/v) as an eluent. ^1^H NMR (500 MHz, CDCl_3_): δ = 8.38 (2H, d, *J* = 7.8 Hz, Ar–H), 8.27 (1H, d, *J* = 7.9 Hz, Ar–H), 7.79 (1H, dd, *J* = 7.6 Hz, *J* = 0.9 Hz, Ar–H), 7.64 (1H, t, *J* = 7.4 Hz, Ar–H), 7.55 (1H, ddd, *J* = 8.0 Hz, *J* = 7.9 Hz, *J* = 1.0 Hz, Ar–H), 7.51 (2H, dd, *J* = 7.8 Hz, *J* = 7.4 Hz, Ar–H), 7.30 (1H, ddd, *J* = 8.0 Hz, *J* = 7.6 Hz, *J* = 0.9 Hz, Ar–H) ppm; ^13^C NMR (125 MHz, CDCl_3_): δ = 180.08, 164.25, 145.99, 134.38, 131.33, 130.62, 130.58 (2C), 128.68 (2C), 128.51, 126.19, 122.56 (t, *J*_C–F_ = 4.1 Hz), 122.47 ppm; ^19^F NMR (470 MHz, CDCl_3_): δ = −134.35 (2F, m, BF_2_) ppm; ^125^Te NMR (158 MHz, CDCl_3_): δ = 780.90 ppm. HRMS (APCI) calcd for C_14_H_10_BN_2_OF_2_Te [M + H]^+^: 400.9916, found: 400.9918.

## Supplementary Material



## Notes

ORTEP diagrams for the X-ray structures and crystal data of compounds **1–4**; (TD)­DFT calculations data; additional photophysical spectra and data; NMR spectra of the synthesized compounds (PDF)

## Abbreviations

RTP: room temperature phosphorescence
OLED: organic light-emitting diode
PDT: photodynamic therapy
BODIPY: 4,4-difluoro-4-bora-3a,4a-diaza-*s*-indacene
HOMO: highest occupied molecular orbital
LUMO: lowest unoccupied molecular orbital
SSE: solid-state emission
PLQY: photoluminescence quantum yield
AIE: aggregation-induced emission
ASE: amplified spontaneous emission
SOCME: spin–orbit coupling matrix elements
SOC: spin–orbit coupling
DIPEA: diisopropylethylamine
CV: cyclic voltammetry
IP: ionization potential
EA: electron affinity
DFT: density functional theory
TD-DFT: time-dependent density functional theory
PMMA: poly­(methyl methacrylate)
fwhm: full width at half-maximum
TRES: time-resolved emission spectroscopy
