# Supplementary material for: Chalcogen-Influenced Benzochalcogenazolo-Based N,O‑Coordinated Difluoroboron Complexes: From Lasing Dyes to Room-Temperature-Phosphorescence Emitters
Source: Inorg Chem. 2025 Jul 7;64(28):14704–16. doi: 10.1021/acs.inorgchem.5c02557 (PMC12284858; doi:10.1021/acs.inorgchem.5c02557)
Supplement: Supplementary file 1 [file ic5c02557_si_001.pdf]

Supporting Information

Chalcogen-Influenced Benzochalcogenazolo-Based *N,O*-Coordinated Difluoroboron Complexes: From Lasing Dyes to Room-Temperature Phosphorescence Emitters

Stepan Kutsiy[a,b], Radosław Pytlarz[a], Andrii Hotynchan[c], Paulina H. Marek-Urban[d], Roman Luboradzki[e], Enzo Jean-Woldemar[f], Dmytro Volyniuk[g], Sébastien Chénais[f], Sébastien Forget[f,*], Krzysztof Durka[d,*], Juozas V. Grazulevicius[g,*], Mykhaylo A. Potopnyk[a,c,*]

[a] Institute of Organic Chemistry, Polish Academy of Sciences, Kasprzaka 44/52, 01-224, Warsaw, Poland, Email: mykhaylo.potopnyk@icho.edu.pl

[b] Department of Electronic Devices, Lviv Polytechnic National University, Sviatoho Yura sq. 1, Lviv 79013, Ukraine

[c] Department of Organic Chemistry, Faculty of Chemistry, Ivan Franko National University of Lviv, Kyryla and Mefodia 6, Lviv 79005, Ukraine, Email: potopnyk@gmail.com

[d] Faculty of Chemistry, Warsaw University of Technology, Noakowskiego 3, 00-664 Warsaw, Poland, Email: krzysztof.durka@pw.edu.pl

[e] Institute of Physical Chemistry, Polish Academy of Sciences, Kasprzaka 44/52, 01-224, Warsaw, Poland

[f] Laboratoire de Physique des Lasers, Université Sorbonne Paris Nord, CNRS, UMR 7538, F-93430 Villetaneuse, France, Email: sebastien.forget@univ-paris13.fr

[g] Department of Polymer Chemistry and Technology, Kaunas University of Technology, Barsausko 59, LT-51423 Kaunas, Lithuania, Email: juozas.grazulevicius@ktu.lt

Contents

1. Single crystal X-ray diffraction and structure refinement S3

2. Theoretical calculations S12

3. Electrochemical properties S20

4. Absorption and emission properties in solutions S23

5. Photophysical properties of crystalline samples of dyes **1–3** S31

6. Photophysical properties of dye-doped PMMA and zeonex films S32

7. Copies of NMR spectra S36

8. References S50

1. Single crystal X-ray diffraction and structure refinement

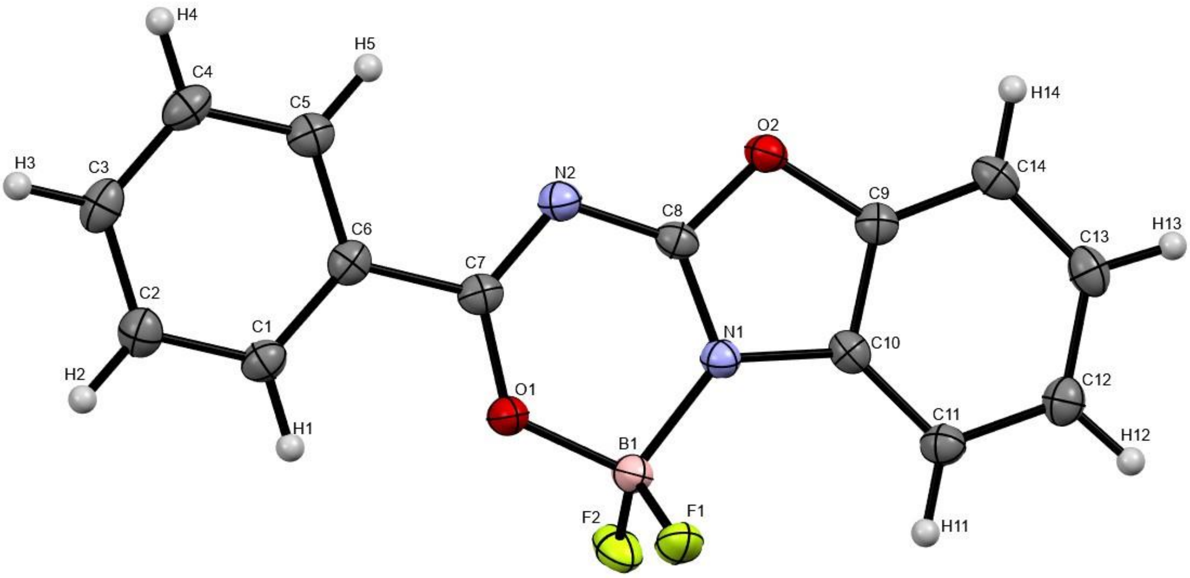

Figure S1. ORTEP diagram of complex **1**. The ellipsoid contour of probability level is 50%.

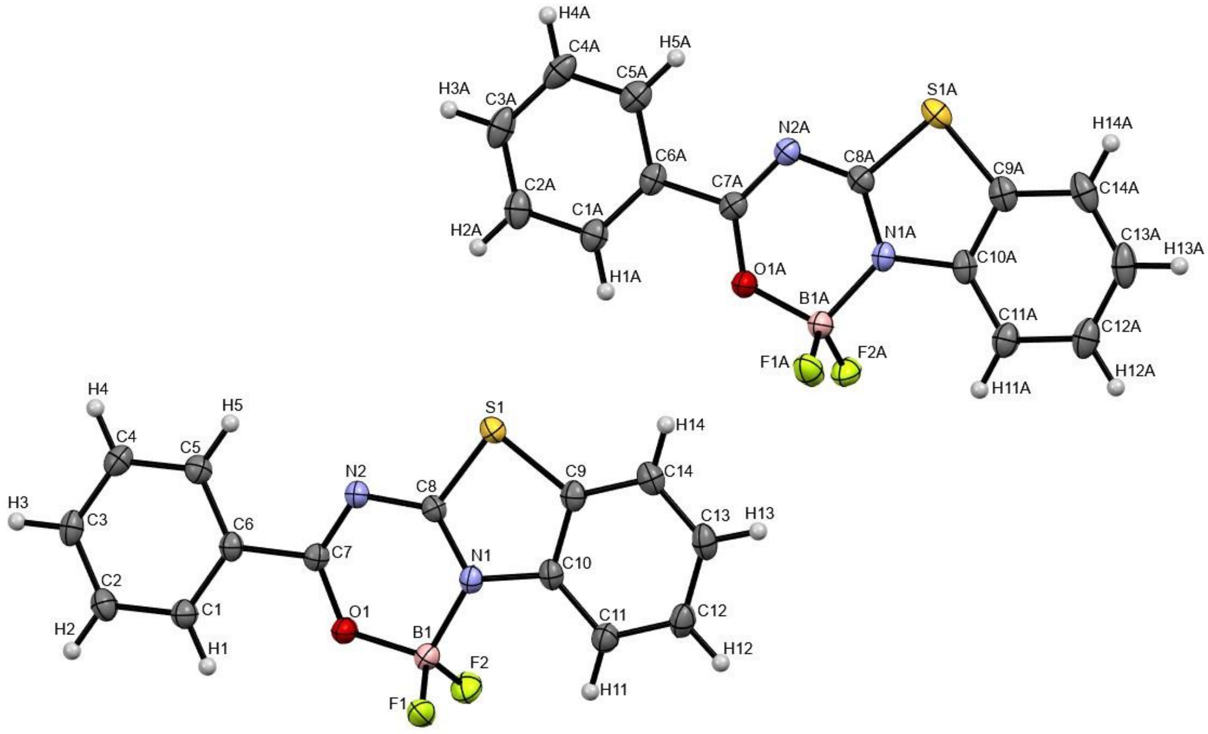

Figure S2. ORTEP diagram of complex **2**. The ellipsoid contour of probability level is 50%.

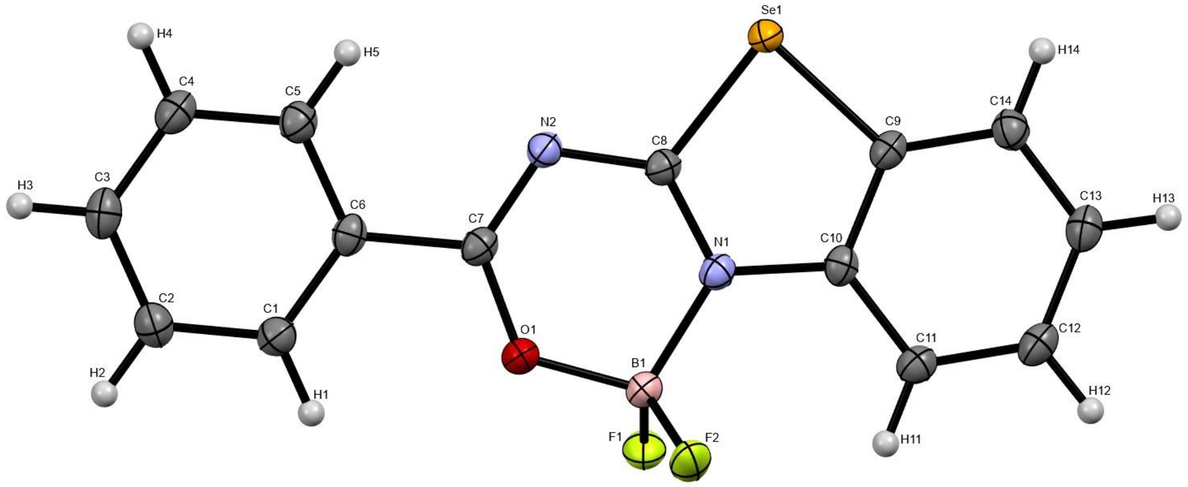

Figure S3. ORTEP diagram of complex **3**. The ellipsoid contour of probability level is 50%.

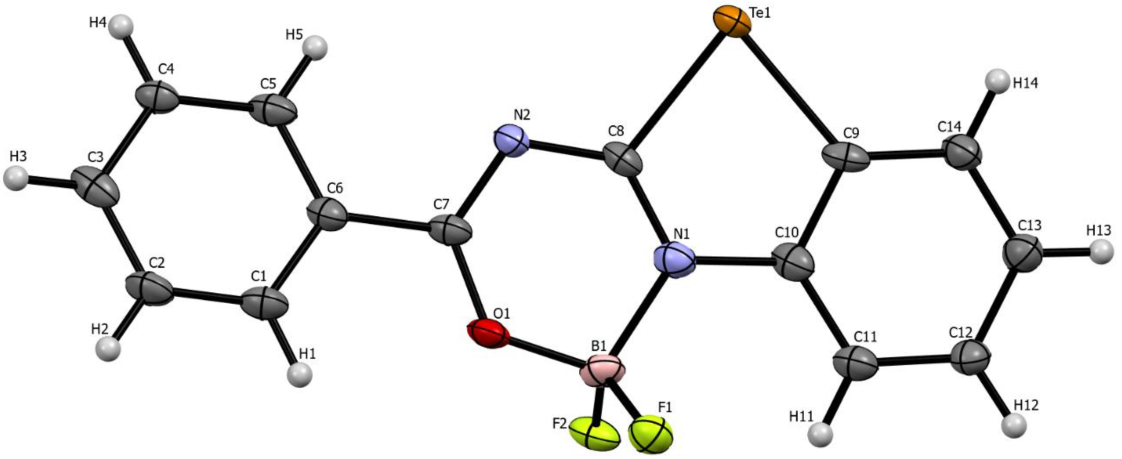

Figure S4. ORTEP diagram of complex **4**. The ellipsoid contour of probability level is 50%.

Table S1. Crystal data for compound 1.

Compound	**1**	
Empirical formula	$C_{14}H_9BF_2N_2O_2$	
Moiety formula	$C_{13}H_{14}BF_2N_3OS$	
Formula weight	286.061	
CCDC No.	CCDC2405618	
Wavelength	1.54184	
Crystal system	monoclinic	
Space group	$P2_1$/n	
Unit cell dimensions	*a* = 7.14359(7)Å	
	b = 13.1222(1)Å	β = 98.9663(9)°
	c = 13.34419(12)Å	
Volume	1235.594(19)Å^3	
Z	4	
Density Calc.	1.538 g/cm^3	
Absorption coefficient	1.054 mm^{-1}	
F(000)	586.398	
Crystal	Colorless block	
Crystal size	0.3 × 0.1 × 0.05 mm	
Index ranges	-7 ≤ h ≤ 8, -15 ≤ k ≤ 15, -16 ≤ l ≤ 16	
Reflections collected (all / independent)	22457 / 2321 [R_{int} = 0.0185]	
Absorption correction	multi-scan	
Refinement method	Gauss-Newton minimization	
Restraints / parameters	0 / 190	
Goodness-of-fit on F^2	1.0744	
Final R indices [F^2 > 2σ(F^2)]	R_1 = 0.0299, wR_2 0.0781	
R indices (all data)	R_1 = 0.0301, wR_2 = 0.0782	

Table S2. Crystal data for compound 2.

Compound	**2**	
Empirical formula	$2(C_{14}H_9BF_2N_2OS)$	
Moiety formula	$C_{14}H_9BF_2N_2OS$	
Formula weight	604.255	
CCDC No.	CCDC2406087	
Wavelength	1.54184	
Crystal system	monoclinic	
Space group	$P2_1$/c	
Unit cell dimensions	*a* = 13.85627(8) Å	
	b = 25.27260(15) Å	β = 98.1991(6) º
	c = 7.30630(5) Å	
Volume	2532.40(3) Å^3	
Z	4	
Density Calc.	1.585 g/cm^3	
Absorption coefficient	2.503 mm^{-1}	
F(000)	1239.135	
Crystal	Colorless block	
Crystal size	0.4 × 0.1 × 0.08 mm	
Index ranges	-16 ≤ h ≤ 16, -30 ≤ k ≤ 30, -8 ≤ l ≤ 8	
Reflections collected (all / independent)	83961 / 4793 [R_{int} = 0.0426]	
Absorption correction	multi-scan	
Refinement method	Gauss-Newton minimization	
Restraints / parameters	0 / 379	
Goodness-of-fit on F^2	1.0502	
Final R indices [$F^2 > 2\sigma(F^2)$]	R_1 = 0.0338, wR_2 = 0.0909	
R indices (all data)	R_1 = 0.0340, wR_2 = 0.0910	

Table S3. Crystal data for compound 3.

Compound	**3**	
Empirical formula	$C_{14}H_9BF_2N_2OSe$	
Moiety formula	$C_{14}H_9BF_2N_2OSe$	
Formula weight	349.021	
CCDC No.	CCDC2405615	
Wavelength	1.54184	
Crystal system	monoclinic	
Space group	C2/c	
Unit cell dimensions	*a* = 10.28844(15) Å	
	b = 11.39305(16) Å	β = 98.5281(15) °
	c = 22.7714(4) Å	
Volume	2639.67(7) $Å^3$	
Z	8	
Density Calc.	1.756 g/cm^3	
Absorption coefficient	4.077 mm^{-1}	
F(000)	1374.209	
Crystal	Yellow block	
Crystal size	0.1 × 0.15 × 0.15 mm	
Index ranges	$-12 \leq h \leq 12$, $-13 \leq k \leq 13$, $-27 \leq l \leq 27$	
Reflections collected (all / independent)	24065 / 2510 [R_{int} = 0.0317]	
Absorption correction	multi-scan	
Refinement method	Gauss-Newton minimization	
Restraints / parameters	0 / 190	
Goodness-of-fit on F^2	1.0589	
Final R indices [$F^2 > 2\sigma(F^2)$]	R_1 = 0.0231, wR_2 = 0.0585	
R indices (all data)	R_1 = 0.0232, wR_2 = 0.0585	

Table S4. Crystal data for compound 4.

Compound	**4**
Empirical formula	$C_{14}H_9BF_2N_2OTe$
Moiety formula	$C_{14}H_9BF_2N_2OTe$
Formula weight	397.664
CCDC No.	CCDC2455383
Wavelength	1.54184
Crystal system	orthorhombic
Space group	$P2_12_12_1$
Unit cell dimensions	a = 4.89089(7) Å
	b = 16.29530(19) Å
	c = 16.3703(2) Å
Volume	1304.69 (3) Å^3
Z	4
Density Calc.	2.025 g/cm^3
Absorption coefficient	18.251 mm^{-1}
F(000)	763.705
Crystal	Yellow needle
Crystal size	0.5 × 0.04 × 0.04 mm
Index ranges	$-5 \leq h \leq 4$, $-19 \leq k \leq 19$, $-19 \leq l \leq 20$
Reflections collected (all / independent)	23878 / 2470 [R_{int} = 0.1291]
Absorption correction	multi-scan
Refinement method	Gauss-Newton minimization
Restraints / parameters	0 / 215
Goodness-of-fit on F^2	1.0500
Final R indices [$F^2 > 2\sigma(F^2)$]	R_1 = 0.0330, wR_2 = 0.0852
R indices (all data)	R_1 = 0.0331, wR_2 = 0.0853

Table S5. Selected geometrical parameters of compounds **1–4** obtained from crystallography.

	**1**	**Conformer 2A**	**Conformer 2B**	**3**	**4**
The B1-N1 bond distances (Å)	1.552(2)	1.566(2)	1.567(2)	1.556(3)	1.576(6)
The B1-O1 bond distances (Å)	1.489(2)	1.488(2)	1.479(2)	1.478(2)	1.472(7)
The B1-F1 and B1-F2 bond distances (Å)	1.376(1)<br>1.370(2)	1.373(2)<br>1.373(2)	1.374(2)<br>1.373(2)	1.379(2)<br>1.379(2)	1.385(8)<br>1.360(6)
The O1-C7 bond distances (Å)	1.304(1)	1.304(2)	1.309(2)	1.310(2)	1.301(6)
The C7-N2 bond distances (Å)	1.322(1)	1.311(2)	1.311(2)	1.312(2)	1.321(6)
The N2-C8 bond distances (Å)	1.337(1)	1.352(2)	1.347(2)	1.355(2)	1.337(7)
The C8-N1 bond distances (Å)	1.322(1)	1.327(2)	1.330(2)	1.326(2)	1.328(6)
The C8-O2 (S1, Se1 or Te1) bond distances (Å)	1.345(1)	1.726(2)	1.726(2)	1.870(2)	2.101(5)
The O2 (S1, Se1 or Te1)-C9 bond distances (Å)	1.400(1)	1.743(2)	1.742(2)	1.886(2)	2.079(5)
The C9-C10 bond distances (Å)	1.381(1)	1.398(2)	1.394(2)	1.395(2)	1.402(7)
The C10-N1 bond distances (Å)	1.402(1)	1.404(2)	1.399(2)	1.401(2)	1.420(7)
The O1-B1-N1 angle (deg)	105.04(9)	106.2(1)	106.5(1)	107.2(1)	106.9(4)
The N1-B1-F1/F2 angles (deg)	110.62(9)<br>110.39(9)	110.3(1)<br>110.4(1)	109.6(1)<br>110.4(1)	110.3(2)<br>110.2(2)	110.5(4)<br>109.7(4)
The angles O1-B1-F1/F2 (deg)	109.48(9)<br>109.9(1)	109.4(1)<br>109.4(1)	109.8(1)<br>109.1(1)	109.5(2)<br>108.9(2)	108.0(4)<br>110.7(4)
The F1-B1-F2 angle (deg)	111.2(1)	111.0(1)	111.3(1)	110.7(2)	110.8(4)
The B1-O1-C7 angle (deg)	126.35(9)	125.6(1)	125.4(1)	124.4(1)	124.2(4)
The O1-C7-N2 angle (deg)	125.4(1)	125.4(1)	125.2(1)	125.3(2)	125.2(5)
The C7-N2-C8 angle (deg)	114.34(9)	116.1(1)	116.4(1)	116.3(2)	116.4(4)
The N2-C8-N1 angle (deg)	128.1(1)	126.4(1)	126.2(1)	126.0(2)	126.4(5)
The C8-N1-B1 angle (deg)	120.74(9)	120.3(1)	120.0(1)	120.0(2)	119.0(4)
The N1-C8-O2(S1, Se2, or Te1) angle (deg)	112.46(9)	113.7(1)	113.5(1)	114.0(1)	113.5(3)
The C8-O2(S1, Se1, or Te1)-C9 angle (deg)	105.41(8)	90.33(8)	90.26(8)	85.55(8)	79.7(2)
The O2(S1, Se1, or Te1)-C9-C10 angle (deg)	108.53(9)	110.4(1)	110.5(1)	110.5(1)	111.7(4)
The C9-C10-N1 angle (deg)	106.21(9)	112.5(1)	112.5(1)	115.0(2)	117.2(4)
The C10-N1-C8 angle (deg)	107.38(9)	113.0(1)	113.2(1)	114.9(2)	117.7(4)
The C1-C6-C7-O1 torsion angle (deg)	-2.2(1)	7.5(2)	0.6(2)	-10.5(3)	6.4(7)
The C5-C6-C7-N2 torsion angle (deg)	-2.4(2)	8.5(2)	0.5(2)	-9.1(3)	8.9(8)

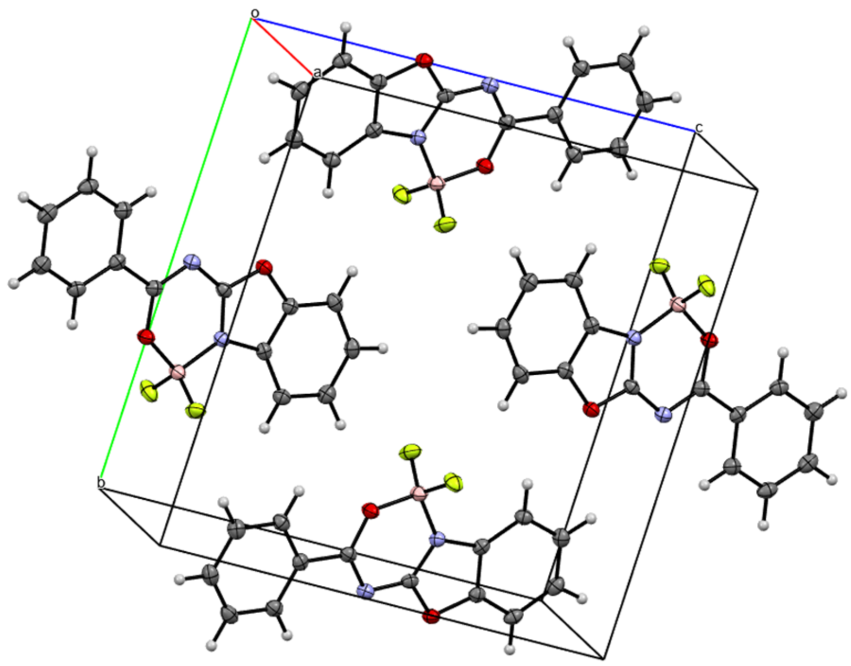

Figure S5. Unit cell of the crystal structure of complex **1**.

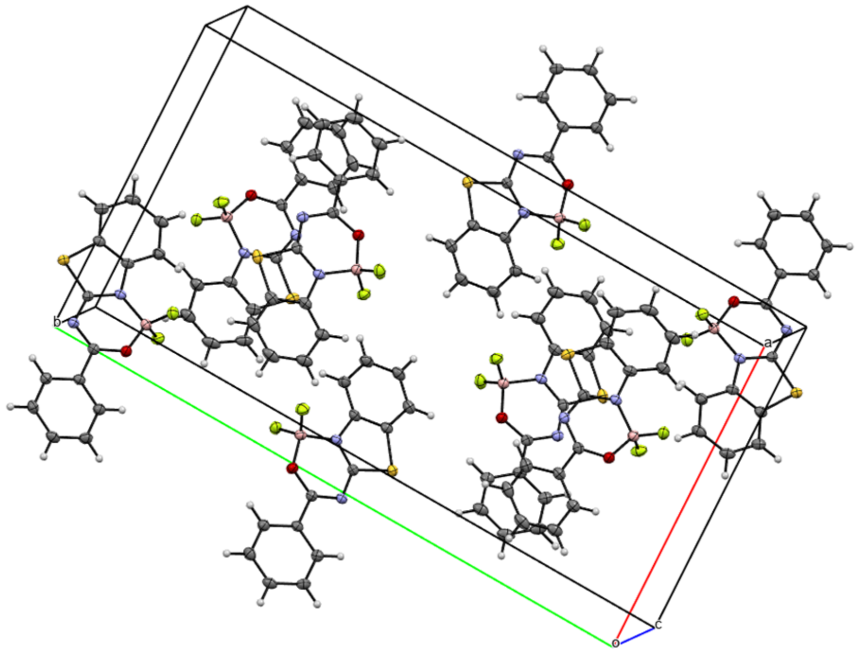

Figure S6. Unit cell of the crystal structure of complex **2**.

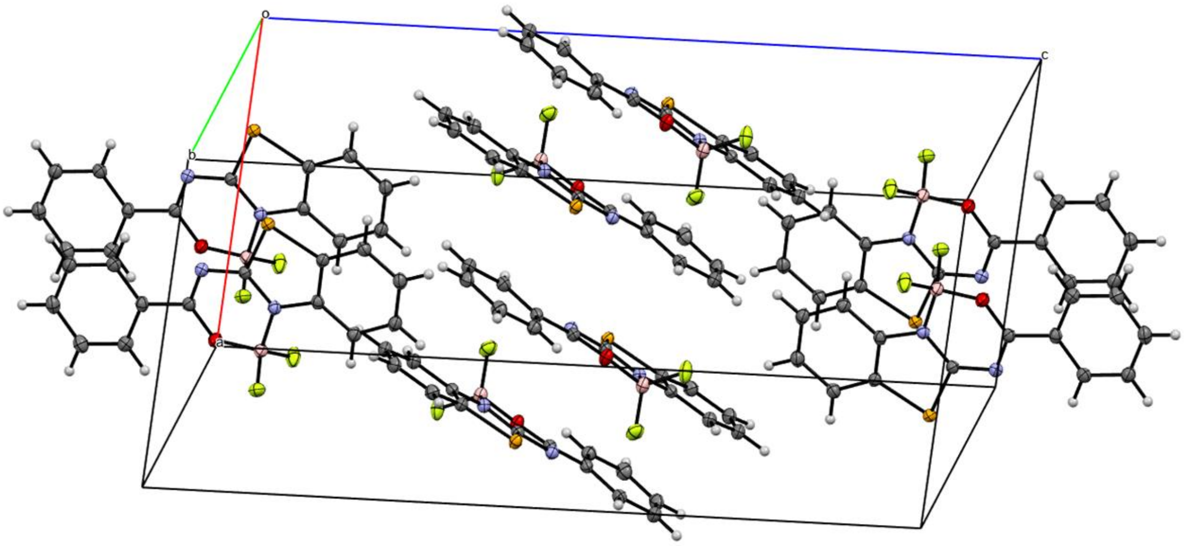

Figure S7. Unit cell of the crystal structure of complex **3**.

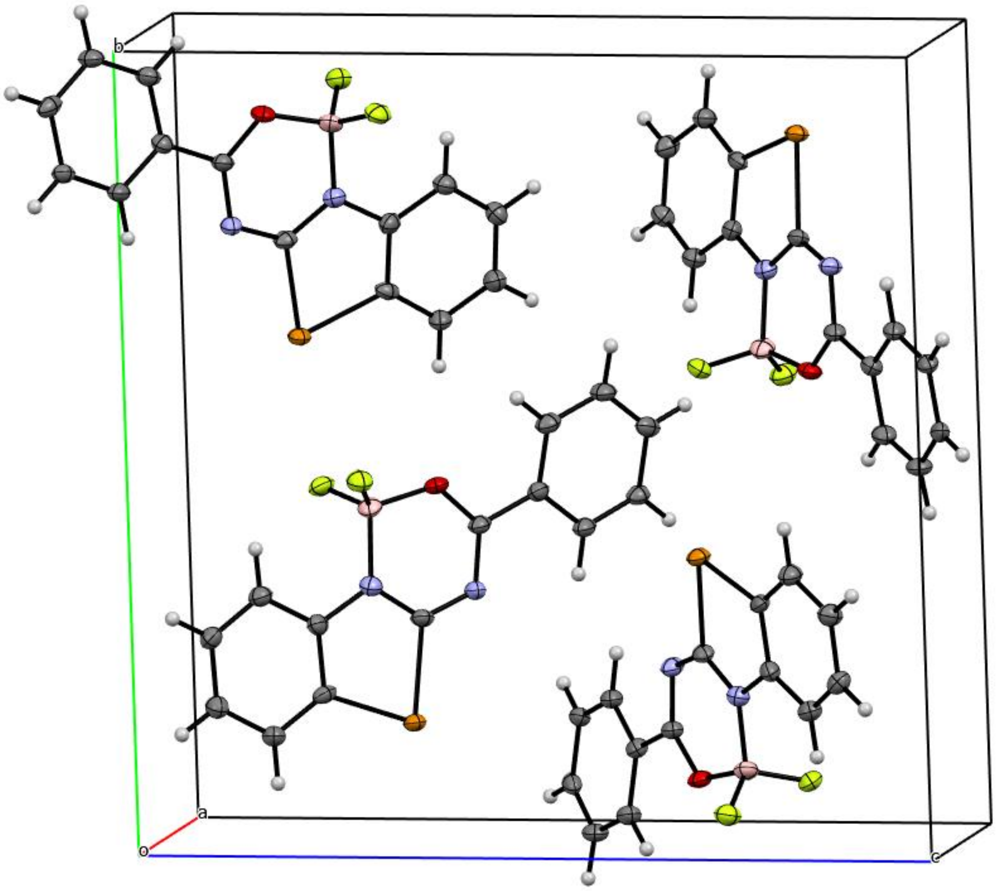

Figure S8. Unit cell of the crystal structure of complex **4**.

2. Theoretical calculations

The ground-state geometries of molecules **1–4** were fully optimized by B3LYP method and 6-31G(d) basis set using Gaussian 16 software package.[1] HOMO and LUMO were visualized with Gaussview 6.0.

Table S6. Calculated properties of the 6 lowest singlet excited states for **1–4** determined through TD-DFT.

Dye	Transition	Energy (eV)	Wavelength (nm)	Oscillator strength	Expansion coefficients for single-electron excitations*
**1**	$S_0 \to S_1$	3.7223	333.08	0.9281	0.70483 H→L
	$S_0 \to S_2$	4.2548	291.40	0.0283	-0.11009 H-3→L+2 / 0.11952 H-2→L /0.67862 H-1→L
	$S_0 \to S_3$	4.3882	282.54	0.0261	0.68674 H-2→L / -0.12195 H-1→L
	$S_0 \to S_4$	4.7134	263.05	0.1870	0.69026 H-3→L
	$S_0 \to S_5$	4.8957	253.25	0.0002	0.70186 H-4→L
	$S_0 \to S_6$	5.2529	236.03	0.0064	0.25741 H-2→L+3 / 0.60110 H→L+2 / -0.13956 H→L+3
**2**	$S_0 \to S_1$	3.5779	346.53	0.9350	0.70479 H→L
	$S_0 \to S_2$	4.0001	309.95	0.0299	0.69779 H-1→L
	$S_0 \to S_3$	4.1708	297.27	0.0258	0.69230 H-2→L
	$S_0 \to S_4$	4.5850	270.41	0.0848	-0.48848 H-4→L / 0.49674 H-3→L
	$S_0 \to S_5$	4.6099	268.95	0.0823	0.50249 H-4→L / 0.47904 H-3→L
	$S_0 \to S_6$	5.1343	241.48	0.0128	-0.16000 H-1→L+1 / -0.28195 H-1→L+2 / 0.14135 H-1→L+5 / 0.48219 H→L+1 / -0.33079 H→L+2
**3**	$S_0 \to S_1$	3.5183	352.40	0.8740	0.70448 H→L
	$S_0 \to S_2$	3.8587	321.31	0.0645	0.69917 H-1→L
	$S_0 \to S_3$	4.1661	297.60	0.0237	0.69236 H-2→L
	$S_0 \to S_4$	4.5027	275.36	0.0052	0.69053 H-4→L / -0.11986 H-3→L
	$S_0 \to S_5$	4.5765	270.92	0.1808	0.11917 H-4→L / 0.67886 H-3→L
	$S_0 \to S_6$	4.7853	259.09	0.0002	-0.19986 H-1→L+2 / 0.11351 H→L+1 / 0.65816 H→L+2
**4**	$S_0 \to S_1$	3.3574	369.29	0.6170	0.70271 H→L
	$S_0 \to S_2$	3.5259	351.64	0.0000	0.16192 H-1→L+1 / 0.68440 H→L+1
	$S_0 \to S_3$	3.6554	339.18	0.2766	0.70109 H-1→L
	$S_0 \to S_4$	3.9841	311.20	0.0002	0.68302 H-1→L+1 / -0.16258 H→L+1
	$S_0 \to S_5$	4.1515	298.65	0.0209	0.10046 H-3→L+4 / 0.69253 H-2→L
	$S_0 \to S_6$	4.3015	288.23	0.0001	0.70189 H-5→L

*H – HOMO, L – LUMO.

Table S7. Hirshfeld atomic charges with hydrogens summed into heavy atoms.

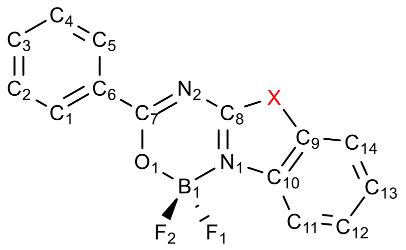

	**1** (X = O)	**2** (X = S)	**3** (X = Se)	**4** (X = Te)
C1-H1	0.0285	0.0274	0.0272	0.0273
C2-H2	0.0189	0.0182	0.0180	0.0179
C3-H3	0.0335	0.0321	0.0318	0.0315
C4-H4	0.0174	0.0163	0.0160	0.0155
C5-H5	0.0236	0.0214	0.0208	0.0202
C6	-0.0161	-0.0157	-0.0157	-0.0154
C7	0.1946	0.1908	0.1904	0.1905
**C8**	**0.2088**	**0.1185**	**0.0992**	**0.0981**
**C9**	**0.0656**	**-0.0244**	**-0.0429**	**-0.0473**
C10	0.0386	0.0442	0.0422	0.0403
C11-H11	0.0349	0.0278	0.0251	0.0228
C12-H12	0.0214	0.0232	0.0219	0.0193
C13-H13	0.0212	0.0192	0.0181	0.0157
C14-H14	0.0277	0.0193	0.0143	0.0083
N1	-0.0610	-0.0548	-0.0567	-0.0573
N2	-0.1980	-0.1988	-0.1998	-0.2028
O1	-0.1501	-0.1517	-0.1513	-0.1494
B1	0.2328	0.2314	0.2310	0.2312
F1	-0.2312	-0.2322	-0.2320	-0.2282
F2	-0.2313	-0.2247	-0.2240	-0.2282
**X**	-0.0797	0.1125	0.1663	0.1899
SUM	0.000	0.000	0.000	0.000
**BF_2**	**-0.230**	**-0.225**	**-0.225**	**-0.225**
**(O,N)-Ligand**	**0.230**	**0.225**	**0.225**	**0.225**
**(O,N)-Ligand**[a]	**0.309**	**0.113**	**0.059**	**0.035**

[a]Ligand without X chalcogen atom.

Optimized geometry for compound 1 obtained using the B3LYP method and 6-31G(d) basis set.

Symbolic Z-matrix:

C	4.98497200	-1.19103100	0.27439100
C	3.59499000	-1.19422700	0.32693000
C	2.86570500	-0.06621000	-0.09546700
C	3.55668100	1.05021800	-0.59907200
C	4.94813000	1.04055300	-0.66082000
H	5.53852600	-2.06056500	0.61603800
H	3.06133400	-2.05921400	0.70417900
H	3.01609300	1.91364600	-0.96718300
H	5.47176200	1.90340300	-1.06109200
C	1.39490800	-0.12494500	-0.02346800
C	-0.84727300	0.74098100	0.09488900
O	0.89389500	-1.32486300	-0.13917400
N	-1.39024000	-0.46460600	-0.00699600
C	-2.99823300	1.08788500	-0.00687000
B	-0.52301700	-1.76998500	0.10107800
F	-0.62126700	-2.29540900	1.36716000
F	-0.89844600	-2.65246900	-0.87855500
C	5.66487500	-0.07379000	-0.21886700
H	6.75026100	-0.07409700	-0.26386100
C	-2.77558100	-0.28712500	-0.08790200
C	-3.83570100	-1.17894800	-0.22768700
H	-3.66797400	-2.24671000	-0.30681200
C	-5.11419500	-0.61887200	-0.26905000
H	-5.97299000	-1.27396200	-0.37644000
C	-4.25444200	1.66457100	-0.04682800
H	-4.39793400	2.73690700	0.01812000
C	-5.32086800	0.76888100	-0.17967600
H	-6.33302500	1.15875900	-0.21802200
C	0.55107700	0.98752300	0.15420700
C	0.98863900	2.31667700	0.39711900
N	1.30151200	3.41861200	0.60844700
O	-1.76571400	1.72133400	0.10806300

Imaginary Frequency = 0

E(RB3LYP) = -1023.62048940 a.u.

E(TD-HF/TD-DFT) = -1023.48369658 a.u.

Optimized geometry for compound 2 obtained using the B3LYP method and 6-31G(d) basis set.

Symbolic Z-matrix:

C	5.09506600	-1.32936200	0.24387400
C	3.70612100	-1.29546900	0.31340700
C	3.00110100	-0.15431400	-0.11455800

C	3.71459500	0.93666600	-0.64182500
C	5.10452400	0.89037600	-0.71966700
H	5.63009100	-2.20871500	0.58994000
H	3.15490100	-2.14137800	0.70839000
H	3.19152500	1.80834300	-1.01622600
H	5.64540100	1.73396100	-1.13774400
C	1.53008600	-0.17436300	-0.02359800
C	-0.70548100	0.78298100	0.09358100
O	0.99491300	-1.36009600	-0.13382300
N	-1.27890200	-0.41406300	-0.00667600
C	-3.17106300	0.95535700	-0.05961700
B	-0.41977800	-1.73036100	0.17572800
F	-0.50914900	-2.15134000	1.48241900
F	-0.83283600	-2.68188500	-0.72501500
C	5.79760600	-0.23653400	-0.27174900
H	6.88199100	-0.26571900	-0.32976700
C	-2.67268100	-0.35902400	-0.10881800
C	-3.54536500	-1.44473200	-0.24830100
H	-3.15644400	-2.45328800	-0.30852700
C	-4.90898700	-1.18084900	-0.31991700
H	-5.60290000	-2.00877800	-0.42727800
C	-4.53924700	1.21743600	-0.13386700
H	-4.91771500	2.23347700	-0.09444600
C	-5.40339200	0.13256600	-0.26245100
H	-6.47296300	0.30844200	-0.32264100
C	0.71355700	0.95245800	0.16155300
C	1.20913700	2.26145800	0.40656300
N	1.54302500	3.35773500	0.61977100
S	-1.84617200	2.10572800	0.09589300

Imaginary Frequency = 0

E(RB3LYP) = -1346.59363050 a.u.

E(TD-HF/TD-DFT) = -1346.46214532 a.u.

Optimized geometry for compound 3 obtained using the B3LYP method and 6-31G(d) basis set.

Symbolic Z-matrix:

C	-5.35797300	1.29523900	0.29236500
C	-3.96818900	1.32079800	0.34683900
C	-3.21915900	0.21742100	-0.10487800
C	-3.89070200	-0.89607000	-0.64009100
C	-5.28210200	-0.90904900	-0.70290400
H	-5.92669900	2.14564500	0.65659000
H	-3.44980700	2.18432800	0.74806100
H	-3.33480900	-1.73915500	-1.03220200
H	-5.79052000	-1.76943400	-1.12745400
C	-1.74959600	0.29925200	-0.02837000
C	0.52961000	-0.56182500	0.06088100

O	-1.26657300	1.50821900	-0.11594500
N	1.05825400	0.65381600	-0.02869100
C	3.08812900	-0.55992200	-0.07141000
B	0.13943900	1.93359800	0.15606500
F	0.24206100	2.37500600	1.45527000
F	0.48379700	2.89422600	-0.76511000
C	-6.01834200	0.18031900	-0.23166200
H	-7.10355000	0.16320200	-0.27794400
C	2.45713400	0.69679100	-0.11511300
C	3.22317800	1.86365500	-0.23753500
H	2.73786100	2.82921100	-0.29498500
C	4.60713900	1.74478800	-0.29756200
H	5.21138100	2.64186100	-0.39109700
C	4.47603600	-0.67457300	-0.13538700
H	4.95809700	-1.64626900	-0.10076400
C	5.23225700	0.48961300	-0.24626900
H	6.31459800	0.42124100	-0.29703300
C	-0.87943700	-0.79050600	0.12112100
C	-1.30750700	-2.12864000	0.33297400
N	-1.58475200	-3.24552300	0.51957500
Se	1.80378700	-1.93439000	0.07524800

Imaginary Frequency = 0

E(RB3LYP) = -3347.78240963 a.u.

E(TD-HF/TD-DFT) = -3347.65311433 a.u.

Optimized geometry for compound 4 obtained using the B3LYP method and 6-31G(d) basis set.

Symbolic Z-matrix:

C	-5.71633700	0.84803200	-0.00023200
C	-4.34806600	1.10743200	-0.00011400
C	-3.43089100	0.04238800	-0.00009000
C	-3.90230600	-1.28190300	-0.00018500
C	-5.27107500	-1.53426000	-0.00029900
H	-6.42178200	1.67376900	-0.00025000
H	-3.97978500	2.12707900	-0.00004900
H	-3.18591600	-2.09539900	-0.00016200
H	-5.63127100	-2.55888400	-0.00037200
C	-1.98313600	0.31192900	0.00004100
C	0.17598200	-0.42649300	0.00010100
O	-1.63003500	1.56656600	-0.00002600
N	-1.13426300	-0.70496500	0.00016000
N	0.71915900	0.79133600	-0.00004800
C	2.86722900	-0.30771900	-0.00015400
B	-0.22285700	2.06638300	0.00036000
F	-0.00546100	2.80153000	1.14500700

F	-0.00520400	2.80247900	-1.14363700
C	-6.17995000	-0.47100500	-0.00032300
H	-7.24801700	-0.67034600	-0.00040800
C	2.12400300	0.89146600	-0.00019300
C	2.79552700	2.12452000	-0.00038500
H	2.23271400	3.04921800	-0.00050200
C	4.18581100	2.14193400	-0.00051800
H	4.70372900	3.09626800	-0.00063300
C	4.26258400	-0.27967100	-0.00025300
H	4.83399200	-1.20263500	-0.00016700
C	4.92043700	0.94916400	-0.00042900
H	6.00608200	0.97741100	-0.00051600
Te	1.59322400	-1.98070100	0.00012600

Imaginary Frequency = 0

E(RB3LYP) = -956.414944095 a.u.

E(TD-HF/TD-DFT) = -956.291563517 a.u.

Spin-orbit coupling (SOC) matrix elements for compounds **1–4** were calculated with PBE0 method using ZORA-DEF2-TZVP basis set and RI-SOMF(1X) function to accelerate the SOC integrals in Orca 5.0. For Te additional basis set SARC-ZORA-TZVP were implemented.

Table S8. Calculated SOCMEs of the triplet states with the singlet excited and ground states for dyes **1–4** calculated with PBE0 method using DEF2-TZVP basis set in Orca 5.0.

Dye	T	S	X	Y	Z	$\vert \langle T \vert HSO \vert S \rangle \vert^2$
**1**	1	0	(0.00. -1.26)	(0.00. 0.00)	(0.00. 0.00)	1.26
	1	1	(0.00. 0.49)	(0.00. 0.00)	(0.00. 0.00)	0.49
	2	1	(0.00. -0.37)	(0.00. 0.00)	(0.00. 0.00)	0.37
	3	1	(0.00. -0.48)	(0.00. 0.00)	(0.00. 0.00)	0.48
	4	1	(0.00. 0.27)	(0.00. 0.00)	(0.00. 0.00)	0.27
	5	1	(0.00. -0.35)	(0.00. 0.00)	(0.00. 0.00)	0.35
	6	1	(0.00. 0.36)	(0.00. 0.00)	(0.00. 0.00)	0.36
**2**	1	0	(0.00. 1.66)	(0.00. 0.14)	(0.00. -0.65)	1.78
	1	1	(0.00. -0.36)	(0.00. -0.18)	(0.00. 0.01)	0.40
	2	1	(0.00. 0.17)	(0.00. 0.23)	(0.00. -0.25)	0.38
	3	1	(0.00. 0.72)	(0.00. 0.31)	(0.00. -0.51)	0.94
	4	1	(0.00. 0.13)	(0.00. 0.18)	(0.00. -0.19)	0.29
	5	1	(0.00. -0.57)	(0.00. -0.93)	(0.00. 0.99)	1.47
	6	1	(0.00. -0.30)	(0.00. -0.36)	(0.00. 0.63)	0.79
**3**	1	0	(0.00. -5.10)	(0.00. -1.31)	(0.00. 5.41)	7.55
	1	1	(0.00. 0.02)	(0.00. -1.76)	(0.00. 0.60)	1.86
	2	1	(0.00. -0.10)	(0.00. -0.77)	(0.00. 1.38)	1.58
	3	1	(0.00. 1.09)	(0.00. 2.07)	(0.00. -2.50)	3.42
	4	1	(0.00. 0.30)	(0.00. 1.08)	(0.00. -1.24)	1.67
	5	1	(0.00. -1.00)	(0.00. -4.37)	(0.00. 4.98)	6.63
	6	1	(0.00. 0.24)	(0.00. -9.16)	(0.00. 15.44)	17.95
**4**	1	0	(0.00. 19.52)	(0.00. 0.02)	(0.00. -0.20)	19.52
	1	1	(0.00. -0.53)	(0.00. 0.10)	(0.00. 0.35)	0.64
	2	1	(0.00. 0.07)	(0.00. -164.89)	(0.00. 513.62)	539.44
	3	1	(0.00. 3.40)	(0.00. 0.27)	(0.00. -1.10)	3.58
	4	1	(0.00. -0.56)	(0.00. 0.03)	(0.00. 0.23)	1.14
	5	1	(0.00. 0.87)	(0.00. -0.02)	(0.00. -0.18)	0.89
	6	1	(0.00. -1.16)	(0.00. 0.01)	(0.00. 0.28)	1.19
	1	2	(0.00. 0.00)	(0.00. -30.91)	(0.00. -290.32)	291.96
	1	3	(0.00. -1.08)	(0.00. -0.04)	(0.00. 0.16)	1.09
	1	4	(0.00. -0.01)	(0.00. 8.88)	(0.00. -245.83)	246.00
	1	5	(0.00. 0.20)	(0.00. 0.07)	(0.00. -0.11)	0.24
	1	6	(0.00. -0.03)	(0.00. 149.02)	(0.00. 269.46)	307.92

Table S9. Calculated energies of first six singlet and triplet states for compounds **1–4** using PBE0 method with ZORA-DEF2-TZVP basis set and RI-SOMF(1X) function to accelerate the SOC integrals in Orca 5.0. For Te additional basis set SARC-ZORA-TZVP were implemented.

Dye	Excited state	S_n, eV	T_n, eV
**1**	1	3.91	2.89
	2	4.41	3.38
	3	4.60	3.63
	4	4.90	3.72
	5	4.95	4.29
	6	5.25	4.34
**2**	1	3.76	2.75
	2	4.18	3.29
	3	4.35	3.47
	4	4.66	3.68
	5	4.79	3.90
	6	5.10	4.32
3	1	3.69	2.70
	2	4.02	3.27
	3	4.34	3.38
	4	4.57	3.67
	5	4.76	3.81
	6	4.77	4.31
**4**	1	3.48	2.59
	2	3.53	3.07
	3	3.78	3.11
	4	4.13	3.25
	5	4.32	3.64
	6	4.35	3.72

3. Electrochemical properties

Cyclic voltammetry measurements were carried out using Bio logic SAS SP-200 potentiostat at room temperature. The experimental consisted of solutions of the studied materials in dry acetonitrile with tetrabutylammonium hexafluorophosphate. Measurements were conducted in a three-electrode cell with an Ag/AgCl reference electrode, a glassy carbon electrode as the working electrode, and a platinum wire auxiliary electrode, using ferrocene as the reference.

Table S10. Onset oxidation and reduction potentials, ionization potentials, and electron affinities of compounds **l–4**.

Dye	E^{onset}_{ox} (V) [a]	E^{onset}_{red} (V) [b]	IP (eV) [c]	EA (eV) [d]	E_g (eV) [e]
**1**	1.66	-1.67	6.46	3.13	3.33
**2**	1.59	-1.65	6.39	3.15	3.24
**3**	1.52	-1.61	6.32	3.19	3.13
**4**	0.84	-1.61	5.64	3.19	2.45

[a] E^{onset}_{ox} – oxidation potential. [b] E^{onset}_{red} – reduction potential. [c] IP – the ionization potential obtained from cyclic voltammetry, IP = E^{onset}_{ox} + 4.8. [e] EA – the electron affinity, EA = E^{onset}_{red} + 4.8. [e] Energy gap, E_g = IP – EA.

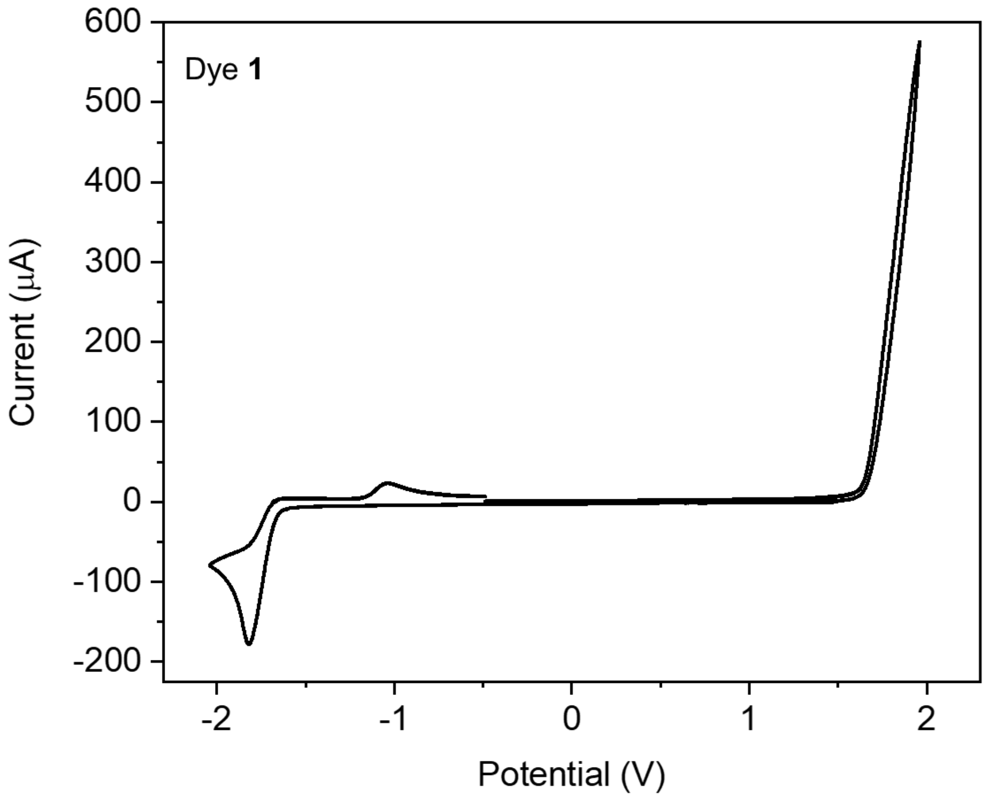

Figure S9. Cyclic voltammogram of compound **1**.

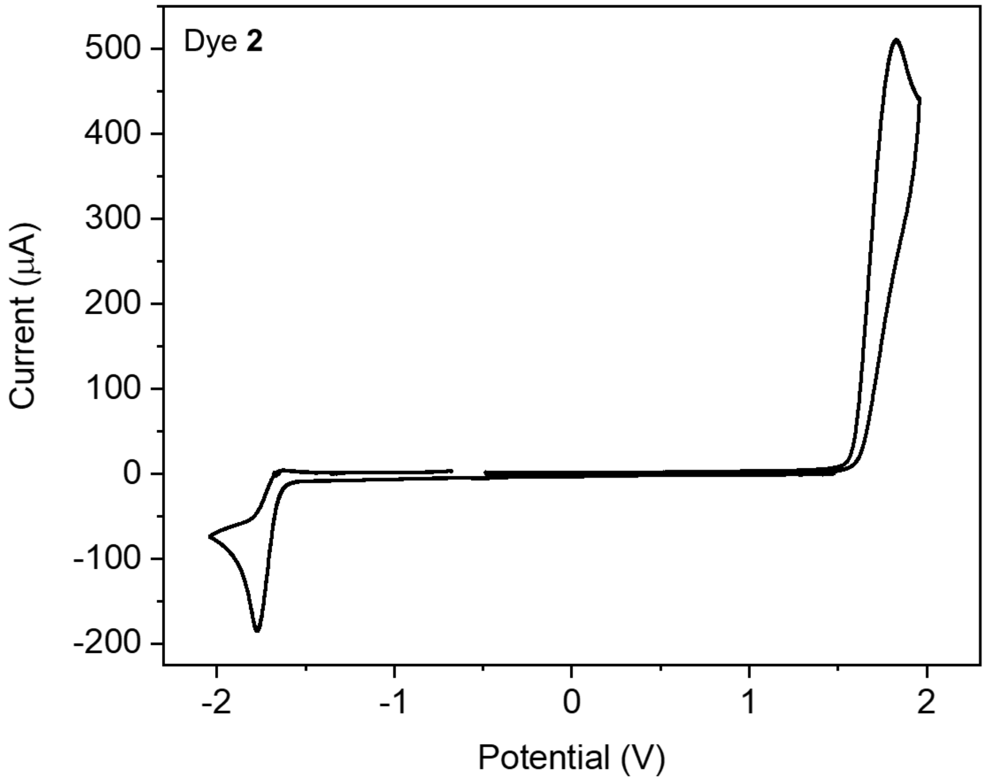

Figure S10. Cyclic voltammogram of compound **2**.

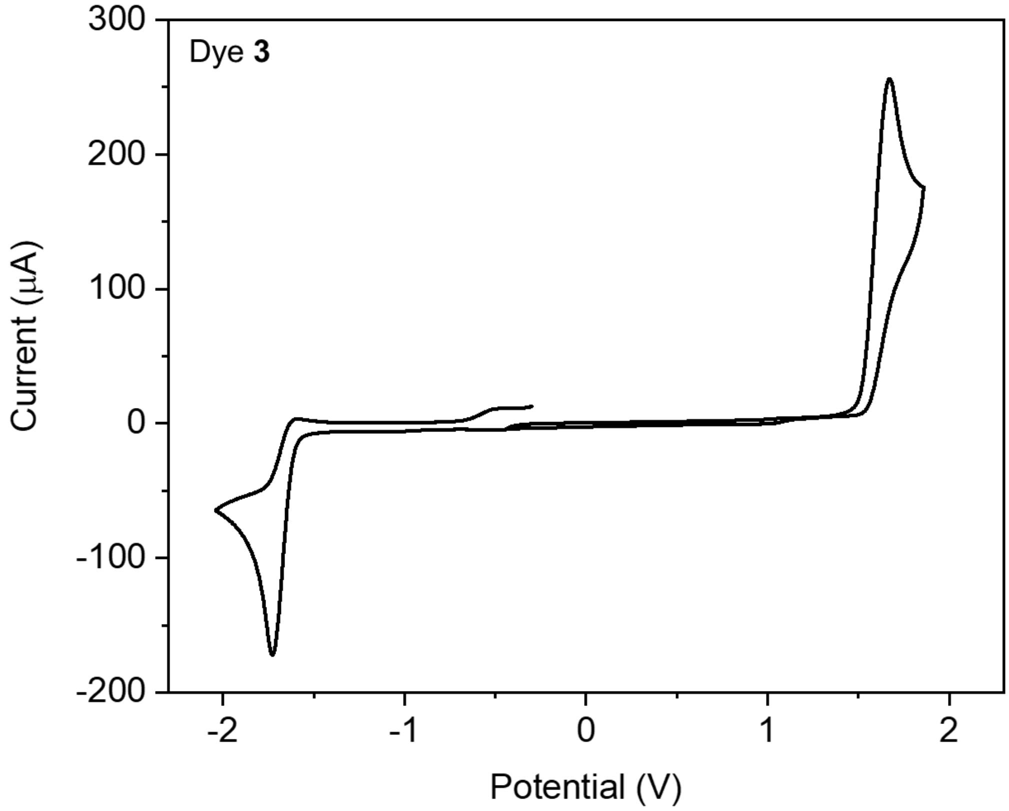

Figure S11. Cyclic voltammogram of compound **3**.

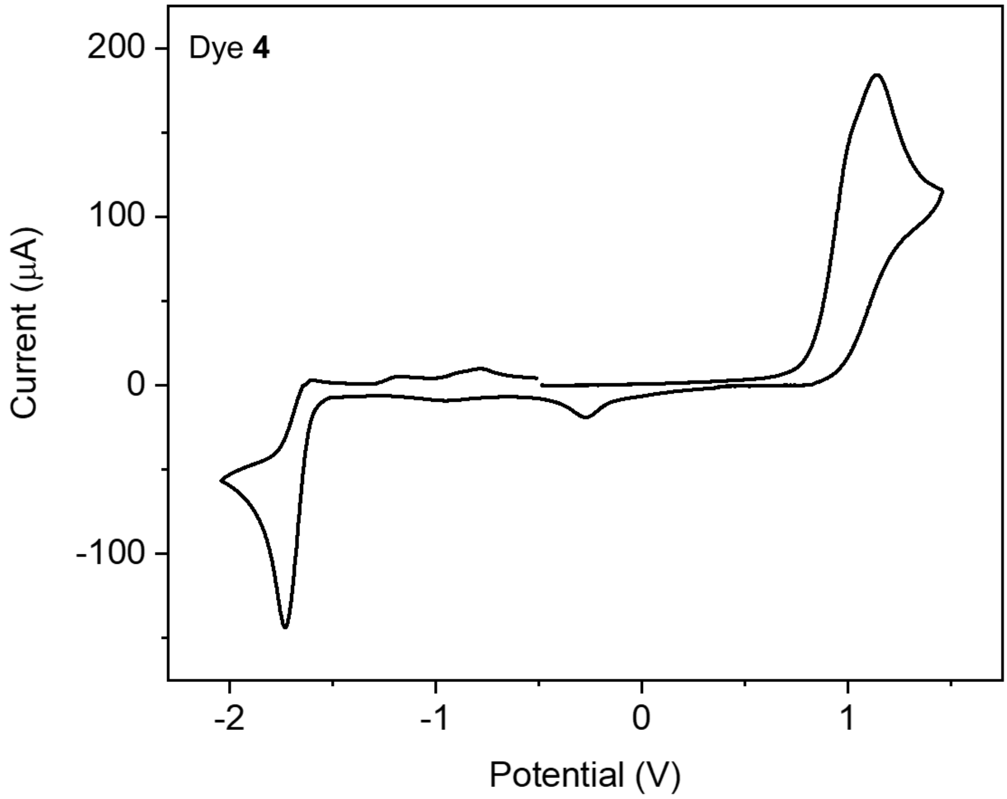

Figure S12. Cyclic voltammogram of compound **4**.

4. Absorption and emission properties in solutions

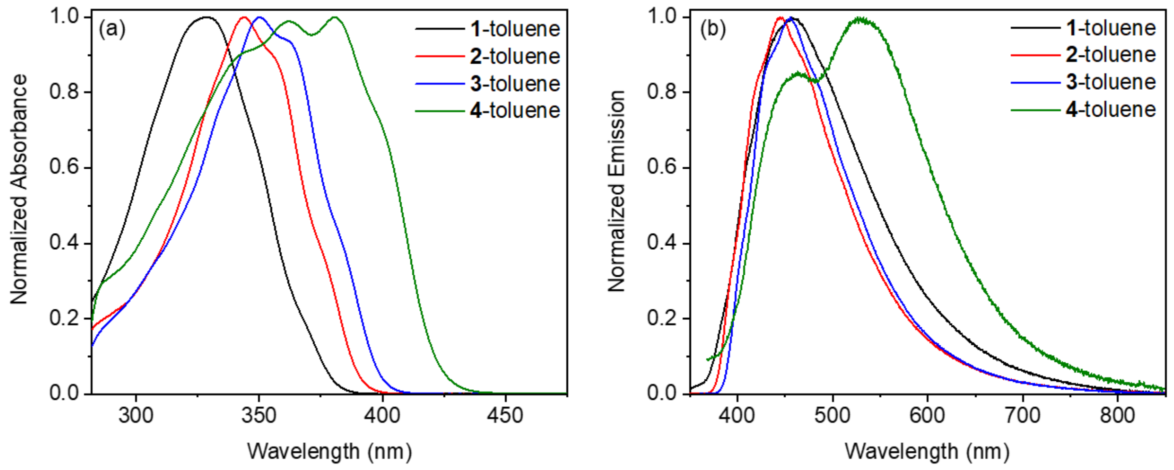

Figure S13. Absorption (a) and photoluminescence (b) spectra of the solutions of dyes **1**–**4** in toluene.

Table S11. Photophysical data for the solutions in toluene of complexes **1**−**4**.

Dye	λ_{abs} [nm] [a)]	ε [$M^{-1}\cdot cm^{-1}$] [b)]	λ_{em} [nm] [c)]	PLQY [%] [d)]
**1**	324	25600	441	1.3
**2**	342	29500	442	5.0
**3**	348	28000	450	4.4
**4**	380	18800	464, 545	0.1

[a)] Absorption maximum; [b)] Molar absorption coefficient; [c)] Photoluminescence maximum; [d)] Photoluminescence quantum yield.

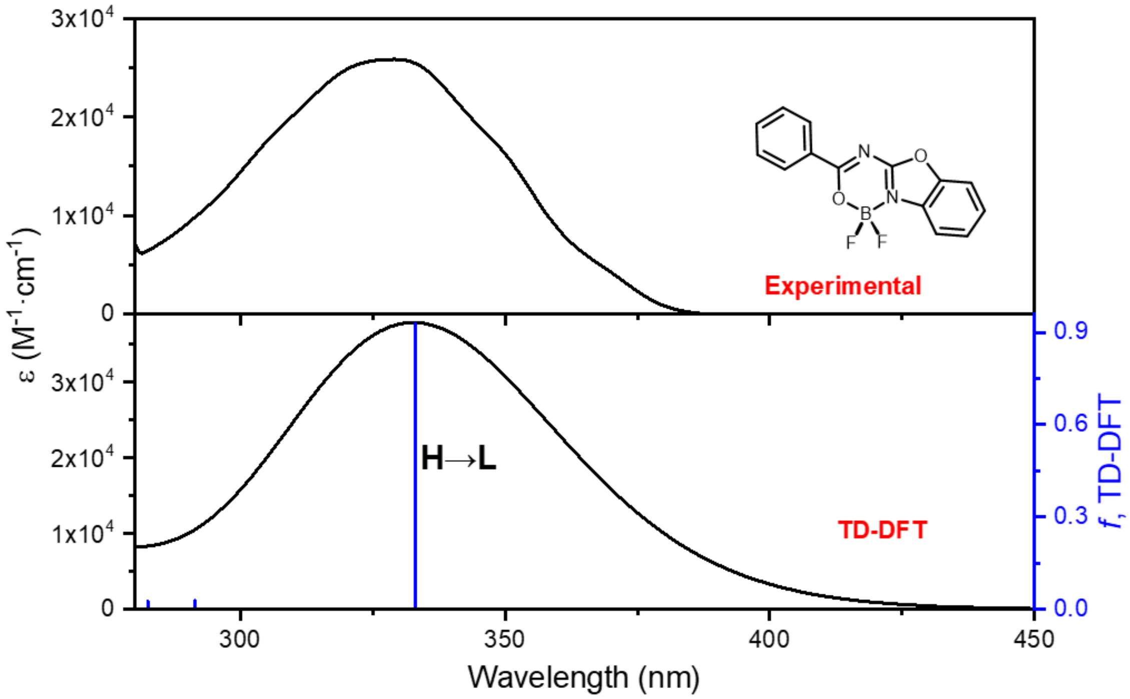

Figure S14. Experimental (top) and TD-DFT-predicted (bottom) absorption spectra of dye **1** in toluene.

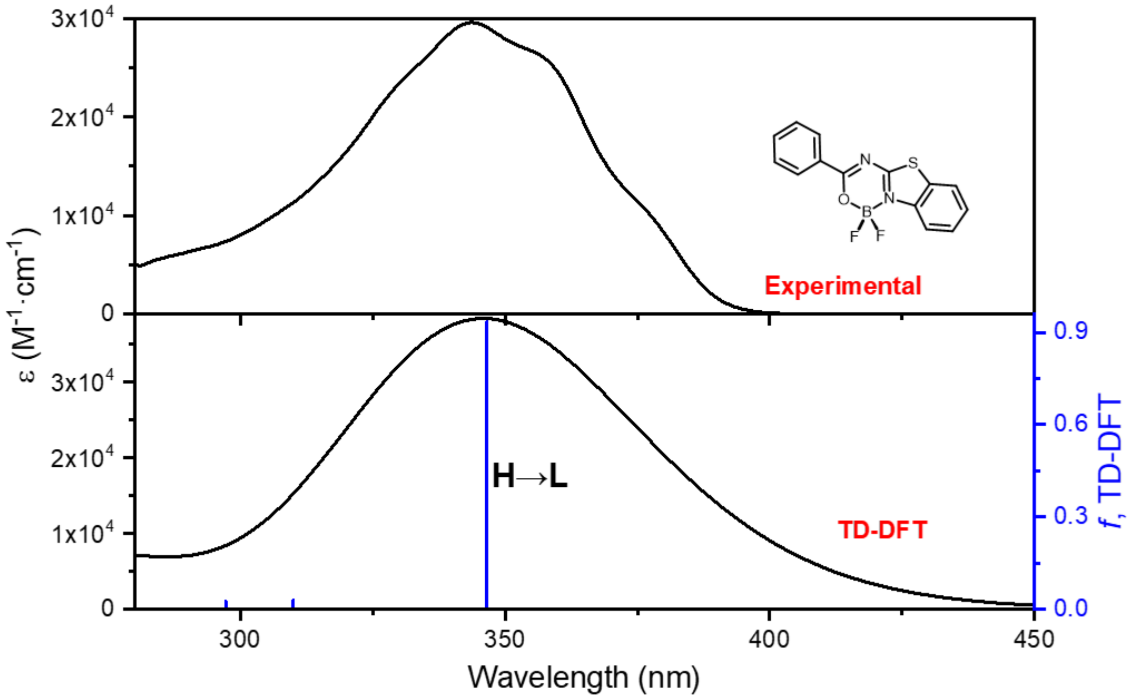

Figure S15. Experimental (top) and TD-DFT-predicted (bottom) absorption spectra of dye **2** in toluene.

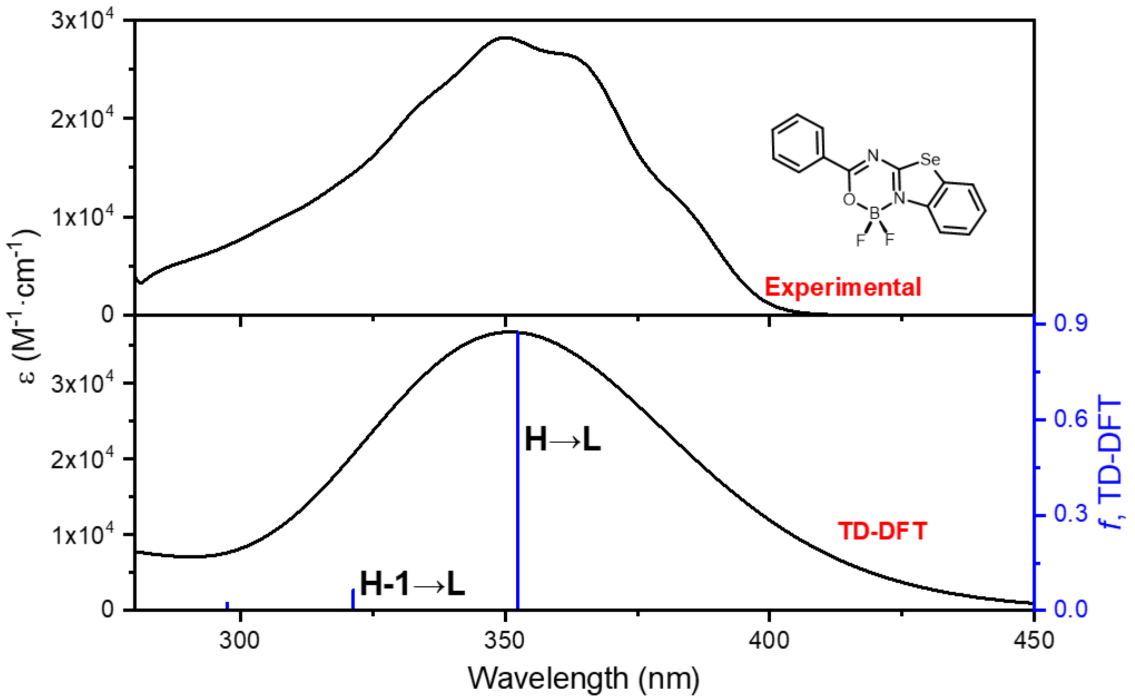

Figure S16. Experimental (top) and TD-DFT-predicted (bottom) absorption spectra of dye **3** in toluene.

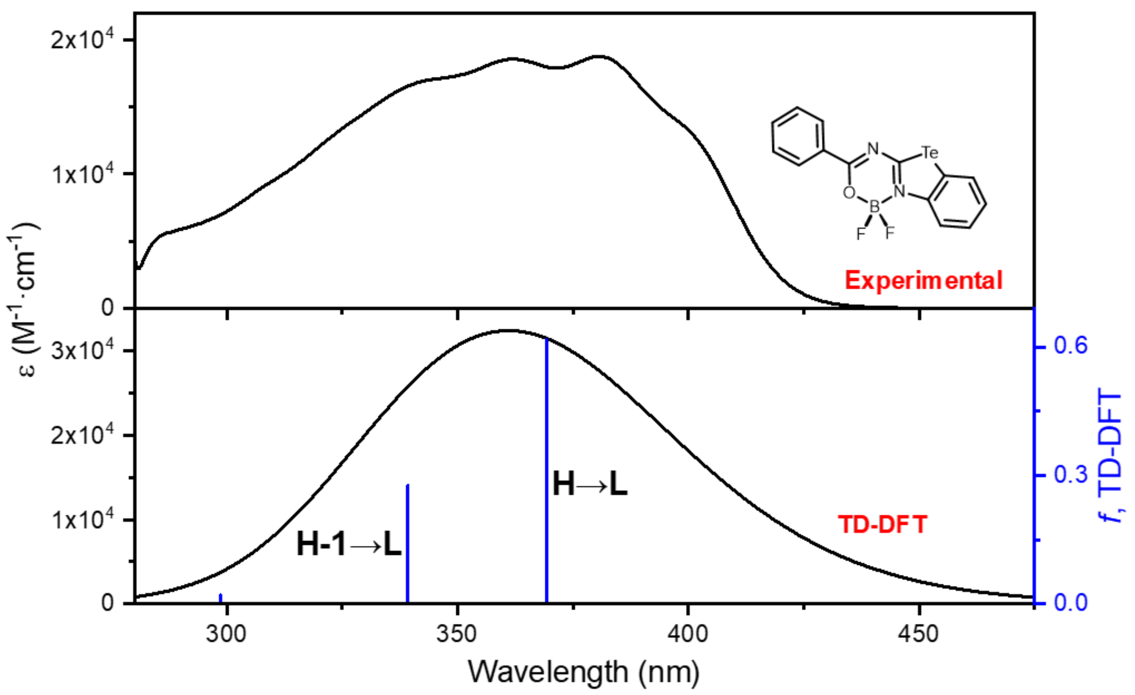

Figure S17. Experimental (top) and TD-DFT-predicted (bottom) absorption spectra of dye **4** in toluene.

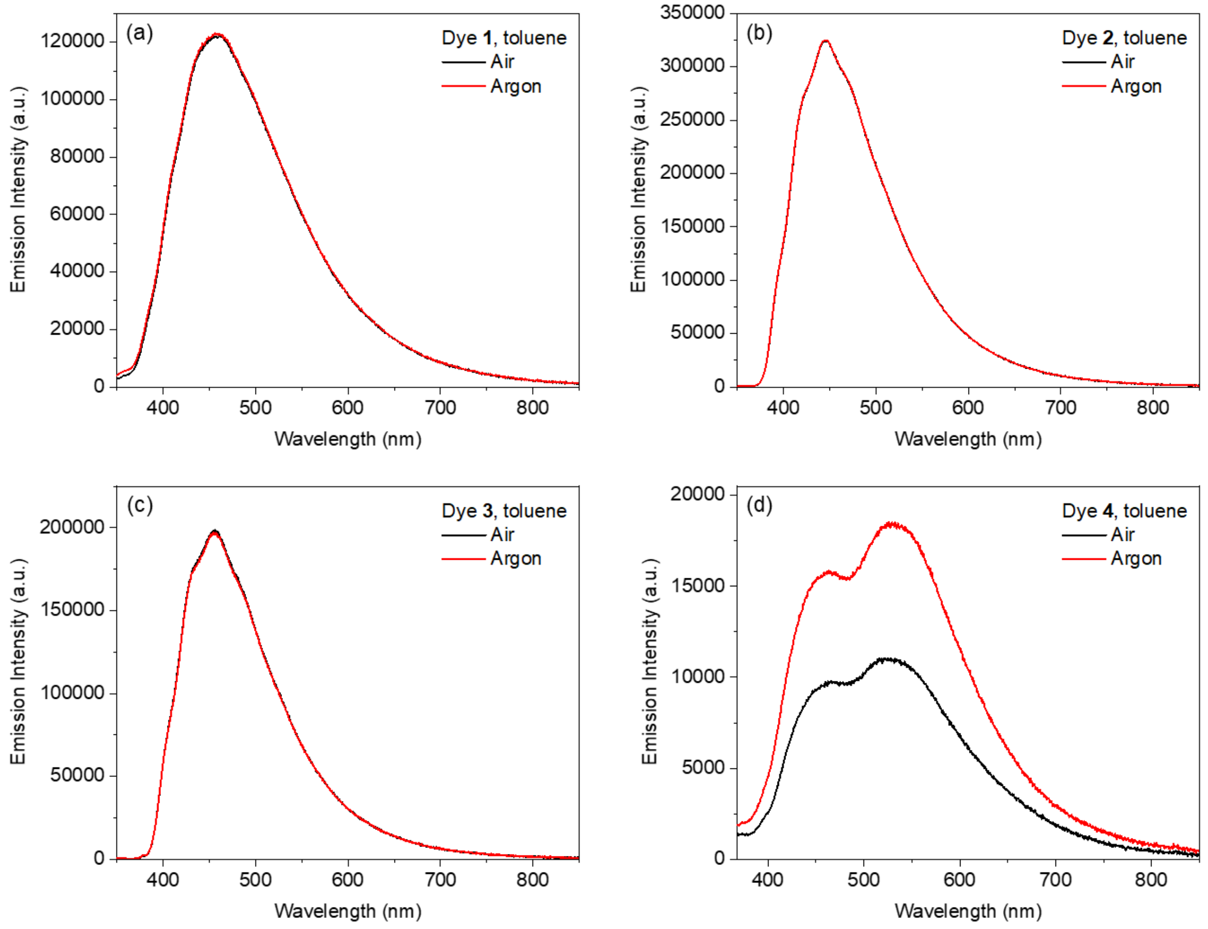

Figure S18. Photoluminescence spectra of the toluene solution of compounds **1** (a), **2** (b), **3** (c), and **3** (d) at air and argon conditions.

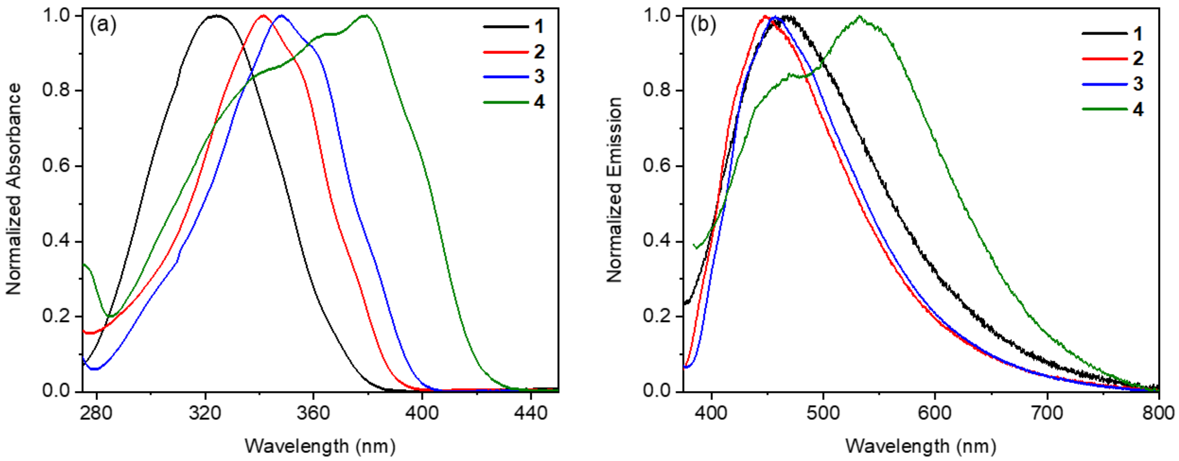

Figure S19. Normalized absorption (left) and emission (right) spectra of compounds **1–4** in tetrahydrofuran.

Table S12. Photophysical data for the solutions in tetrahydrofuran of boron difluoride complexes **1−4**.

Dye	λ_{abs} [nm] [a)]	ε [$M^{-1}\cdot cm^{-1}$] [b)]	λ_{em} [nm] [c)]	PLQY [%] [d)]
**1**	324	38100	466	< 0.1
**2**	341	31000	448	< 0.1
**3**	348	27100	457	< 0.1
**4**	379	21100	532	< 0.1

[a)] Absorption maximum; [b)] Molar absorption coefficient; [c)] Photoluminescence maximum; [d)] Photoluminescence quantum yield.

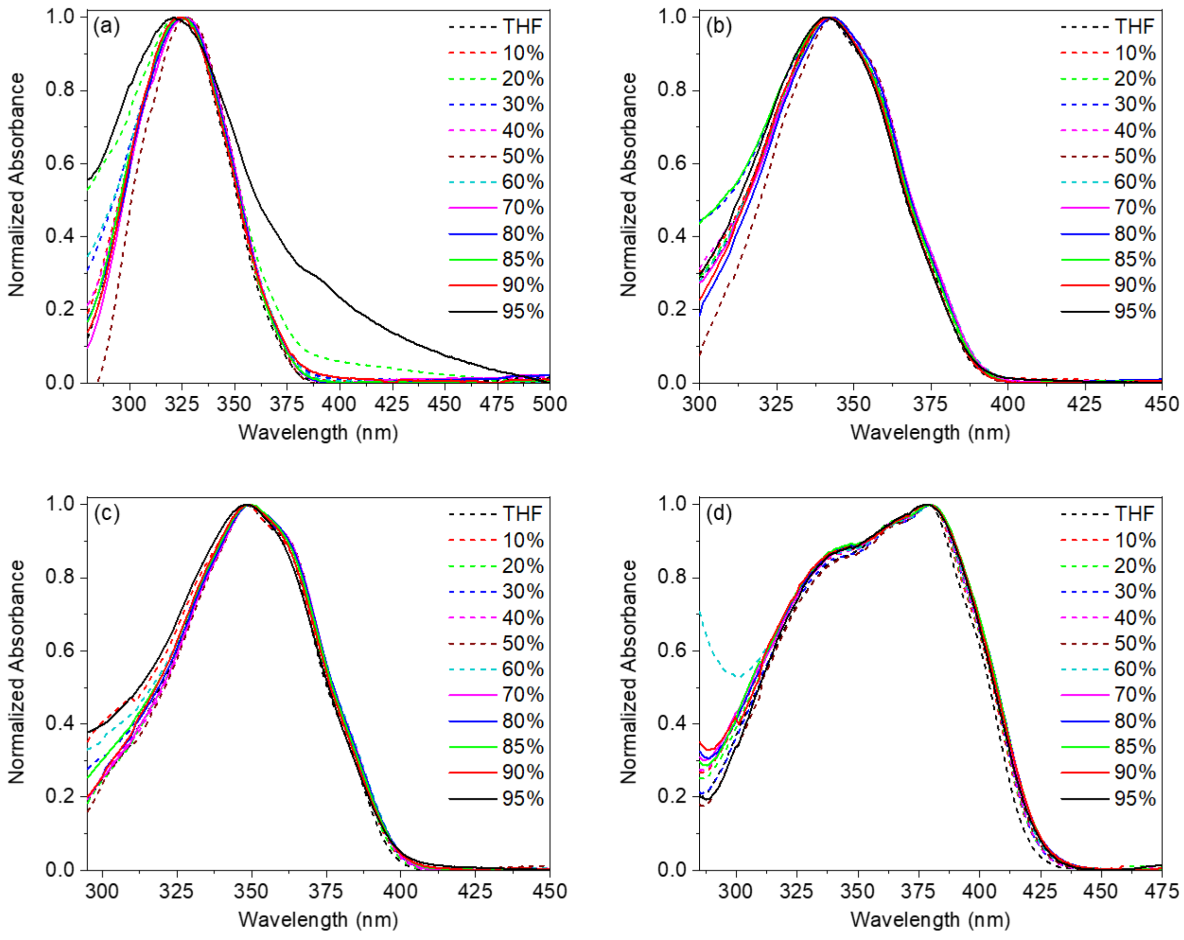

Figure S20. Absorption spectra of the dispersions of dyes **1** (a), **2** (b), **3** (c), and **4** (d) in tetrahydrofuran/water mixtures of varying water content.

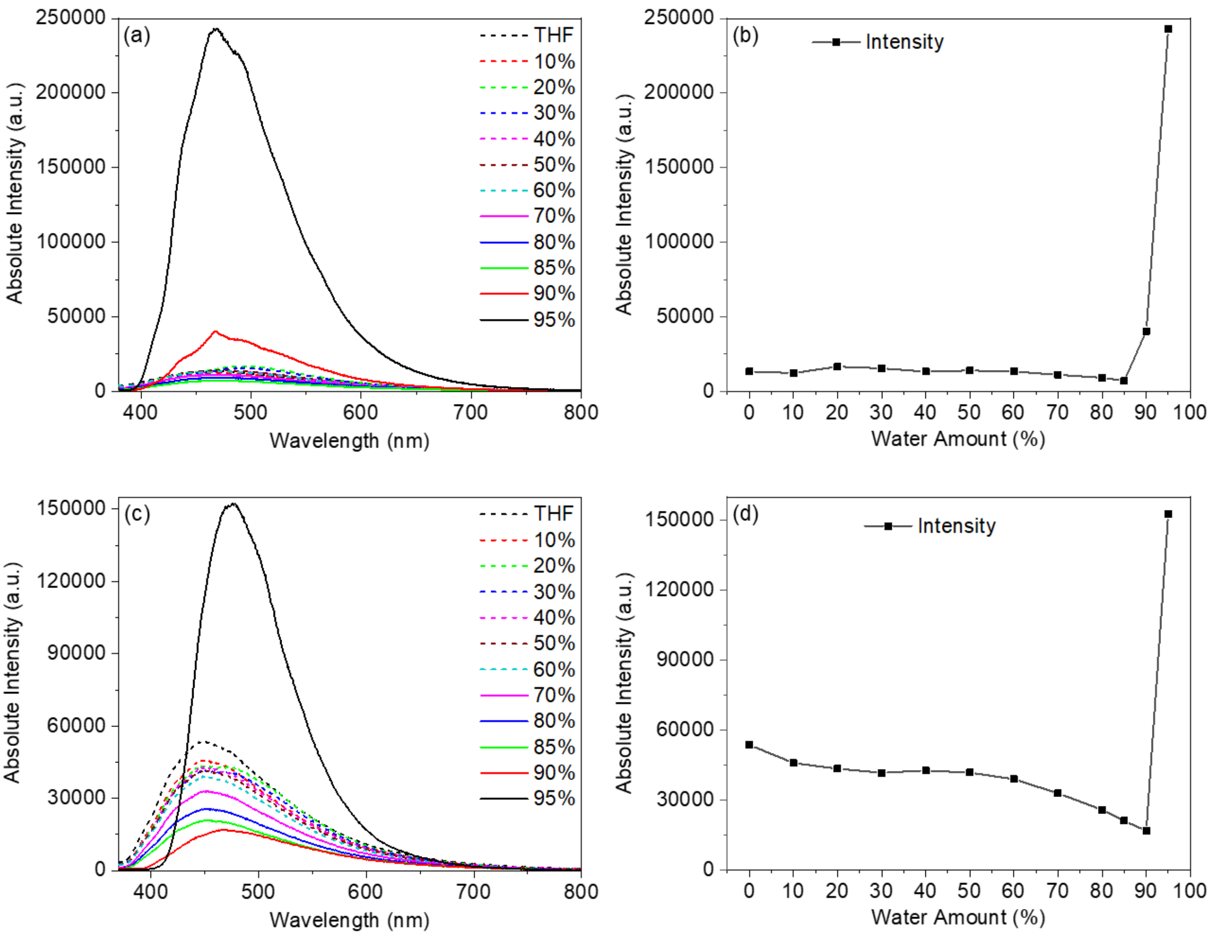

Figure S21. Photoluminescence spectra of the dispersions of boron complexes **1** (a) and **2** (c) in tetrahydrofuran/water mixtures of varying water contents. The dye concentration used was 5.0×10^{-6} M, λ_{ex} = 320 nm for both dyes. Plots of emission intensity of compounds **1** (b) and **2** (b) versus f_w.

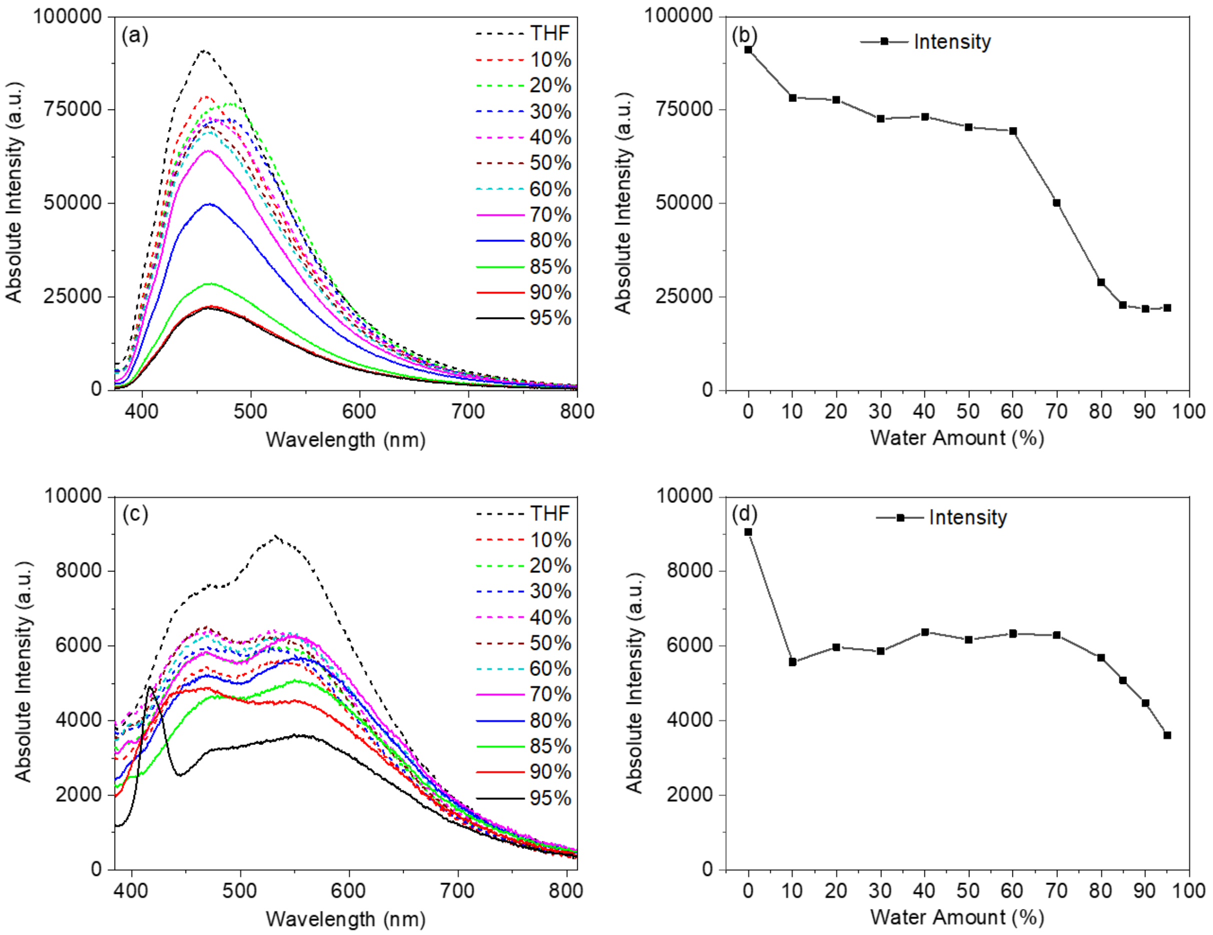

Figure S22. Photoluminescence spectra of the dispersions of boron complexes **3** (a) and **4** (c) in tetrahydrofuran/water mixtures of varying water contents. The dye concentration used was 5.0×10^{-6} M, λ_{ex} = 320 nm for both dyes. Plots of emission intensity of compounds **3** (b) and **4** (d) versus f_w.

5. Photophysical properties of crystalline samples of dyes 1–3

Table S13. Photophysical data for the solid-state samples of complexes **1−3**.

Dye	λ_{em} [nm] [a)]	PLQY [%] [b)]	τ [ns] [c)]
**1**	454	45	2.46
**2**	457	48	2.64
**3**	472	4.0	0.28

[a)] Photoluminescence maximum; [b)] Photoluminescence quantum yield; [c)] Average excited-state lifetime.

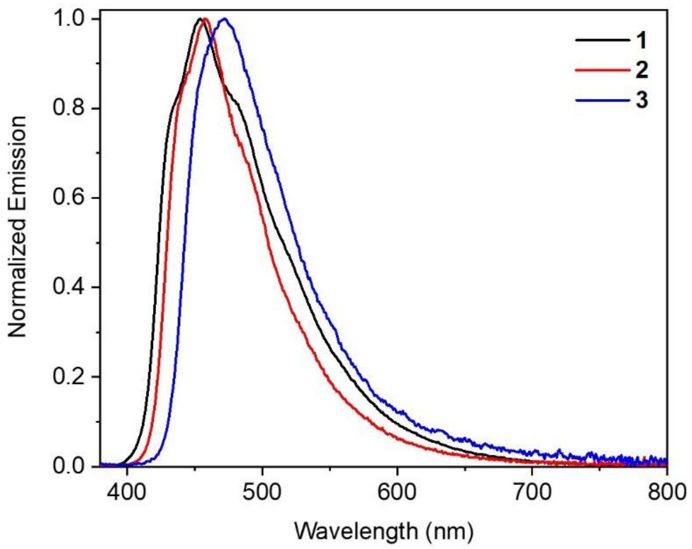

Figure S23. Solid-state photoluminescence spectra of boron difluoride complexes **1–3**.

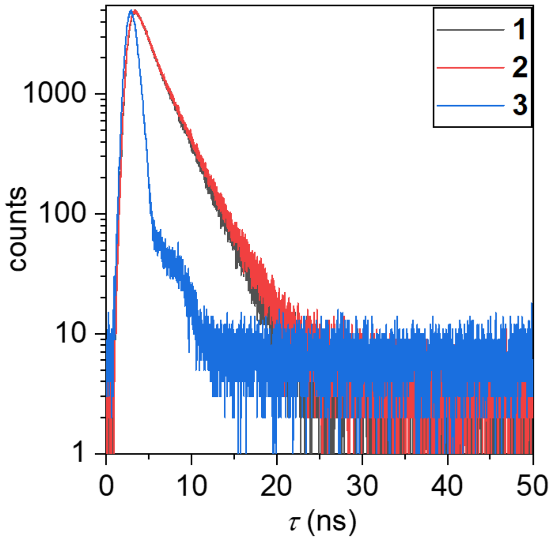

Figure S24. Photoluminescence decay curves of solid-state samples of dyes **1–3**.

6. Photophysical properties of dye-doped PMMA and zeonex films

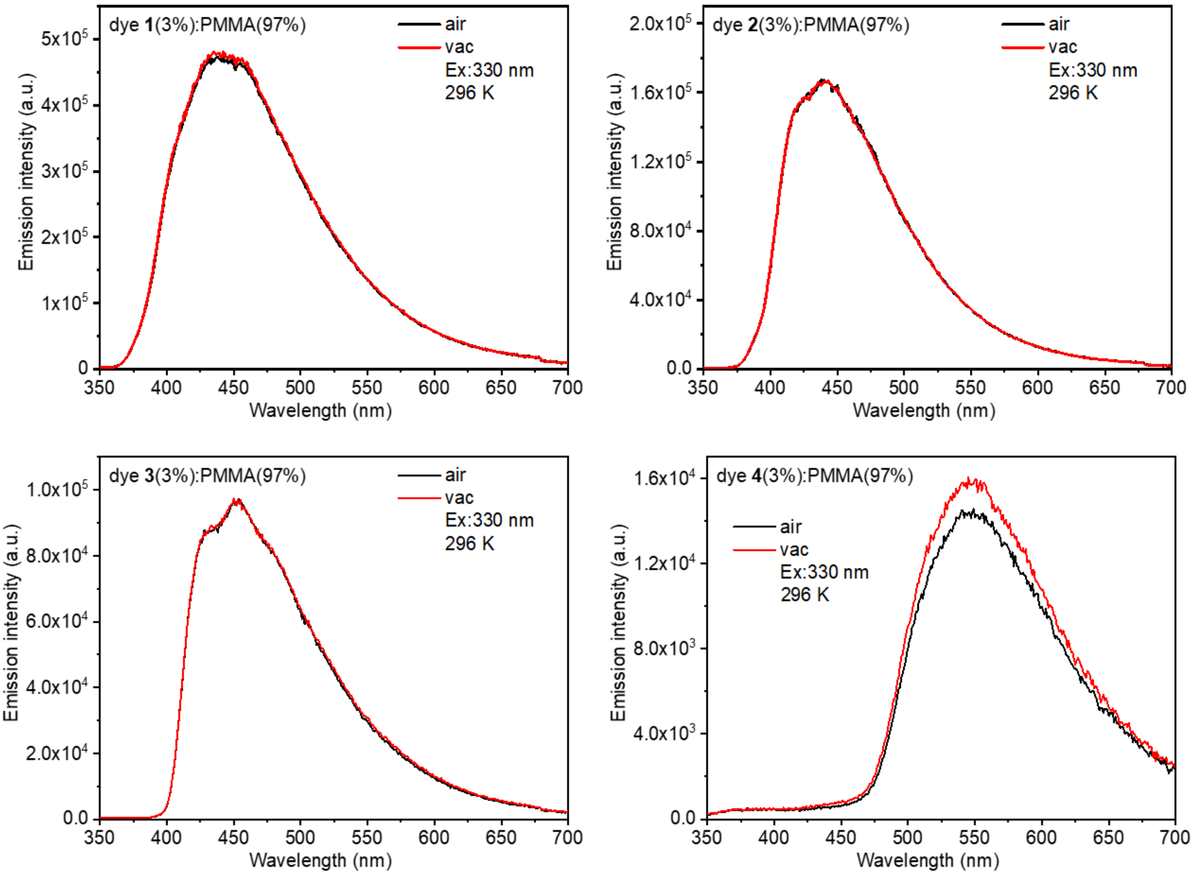

Figure S25. Photoluminescence spectra of PMMA-based films of dyes **1–4** at air and under vacuum.

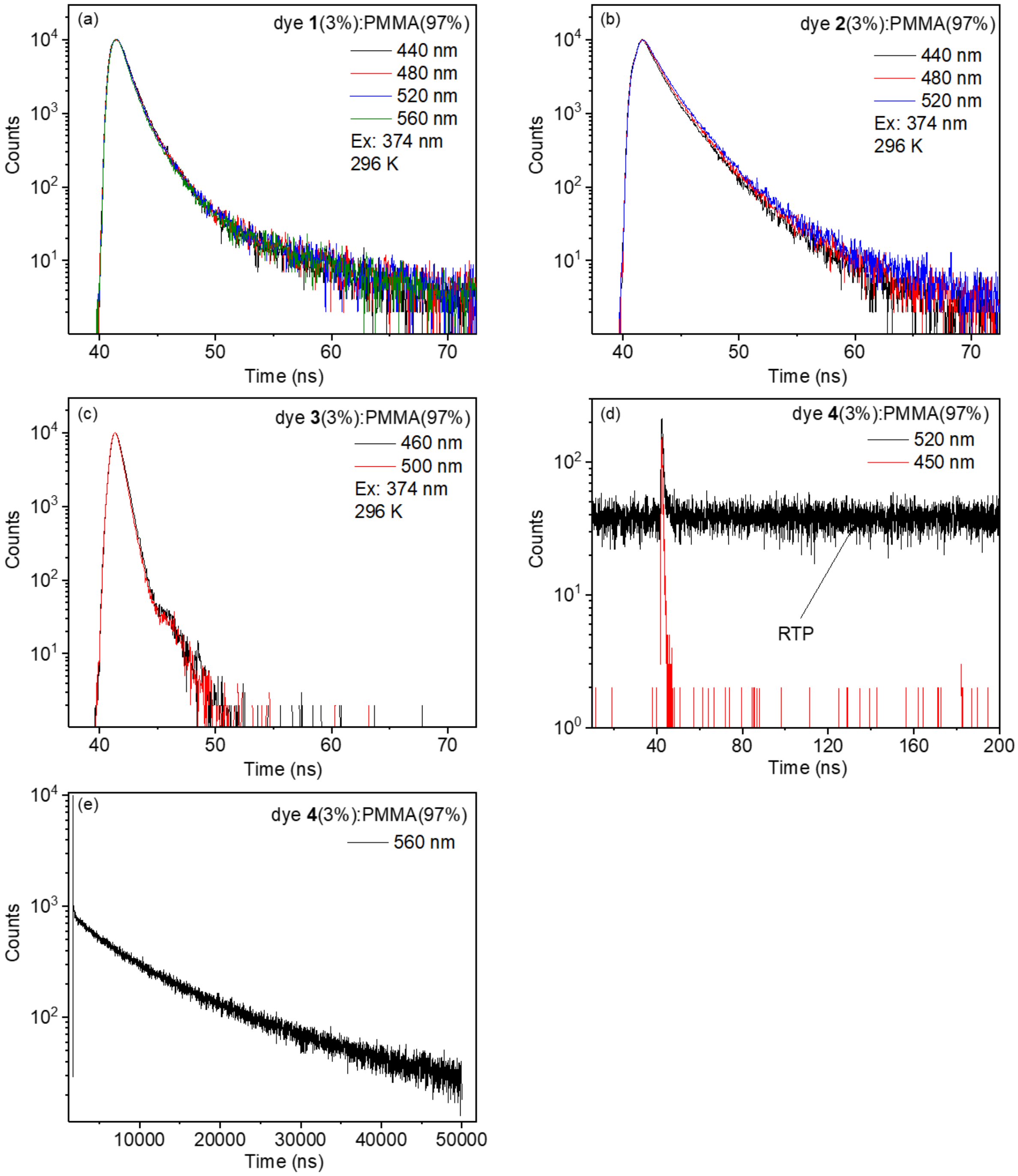

Figure S26. Photoluminescence decay curves of PMMA-based films of dyes **1** (a), **2** (b), **3** (c) and **4** (d, e) in nanosecond (a, b, c, d), microsecond (e) ranges.

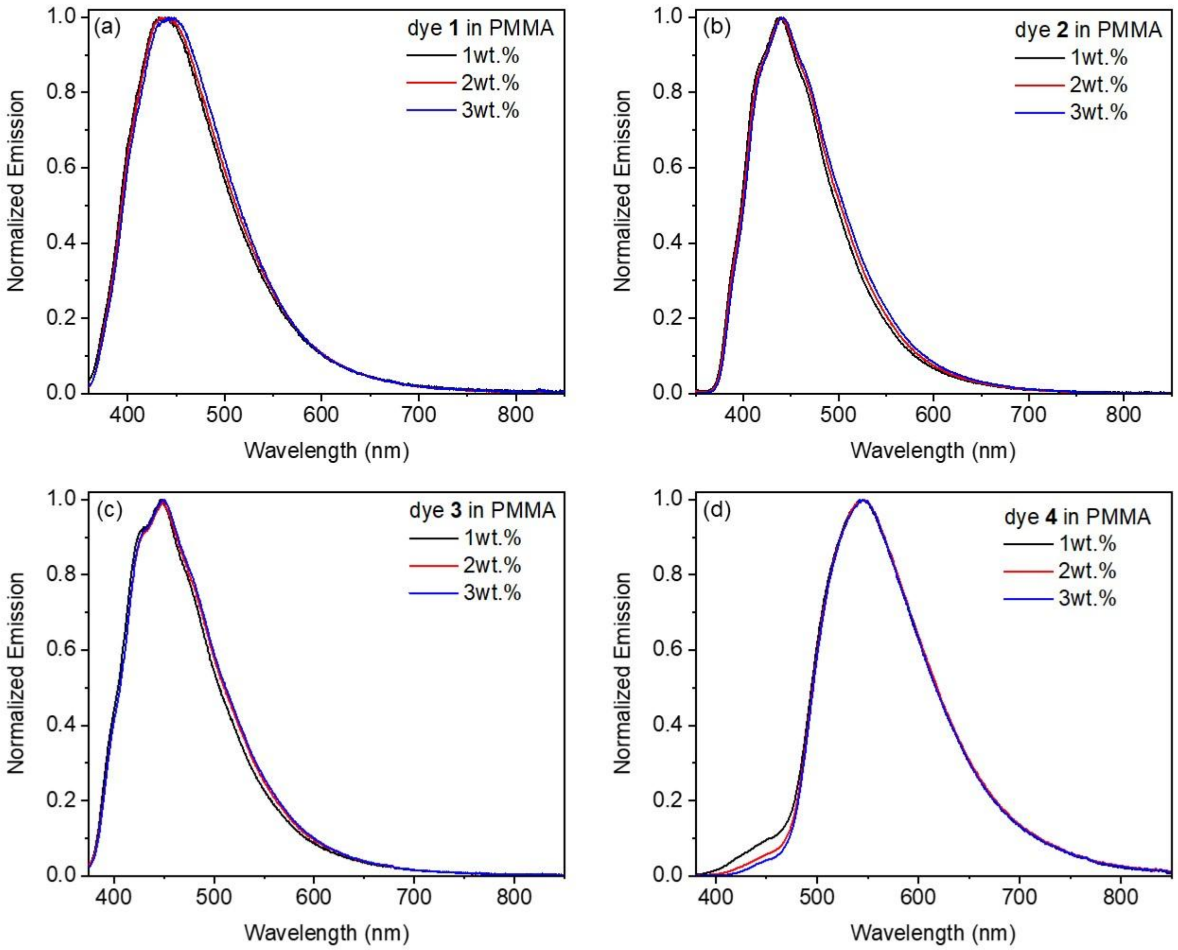

Figure S27. Photoluminescence spectra of PMMA-based films of dyes **1** (a), **2** (b), **3** (c), **4** (d) with 1wt.%, 2wt.% and 3wt.% of the dyes.

Table S14. Photophysical data for PMMA-based films of dyes **1−4**.

Dye	Percentage of dye [%]	λ_{em} [nm] [a)]	PLQY [%] [b)]
**1**	1	440	25.1
	2	442	23.8
	3	444	22.3
**2**	1	439	48.6
	2	440	46.4
	3	440	44.7
**3**	1	448	13.1
	2	449	12.2
	3	449	11.0
**4**	1	545	0.72
	2	545	0.53
	3	545	0.49

[a)] Photoluminescence maximum; [b)] Photoluminescence quantum yield.

Table S15. Lifetimes of dye-doped Zeonex film of dye **4** (1% in Zeonex) at different temperatures.

T, K	τ, ns
100	35094.88
120	34558.19
140	31934.46
160	27958.70
180	29598.86
200	22123.41
220	16912.26
240	15819.79
260	12525.94
280	10891.07
300	9613.59

7. Copies of NMR spectra

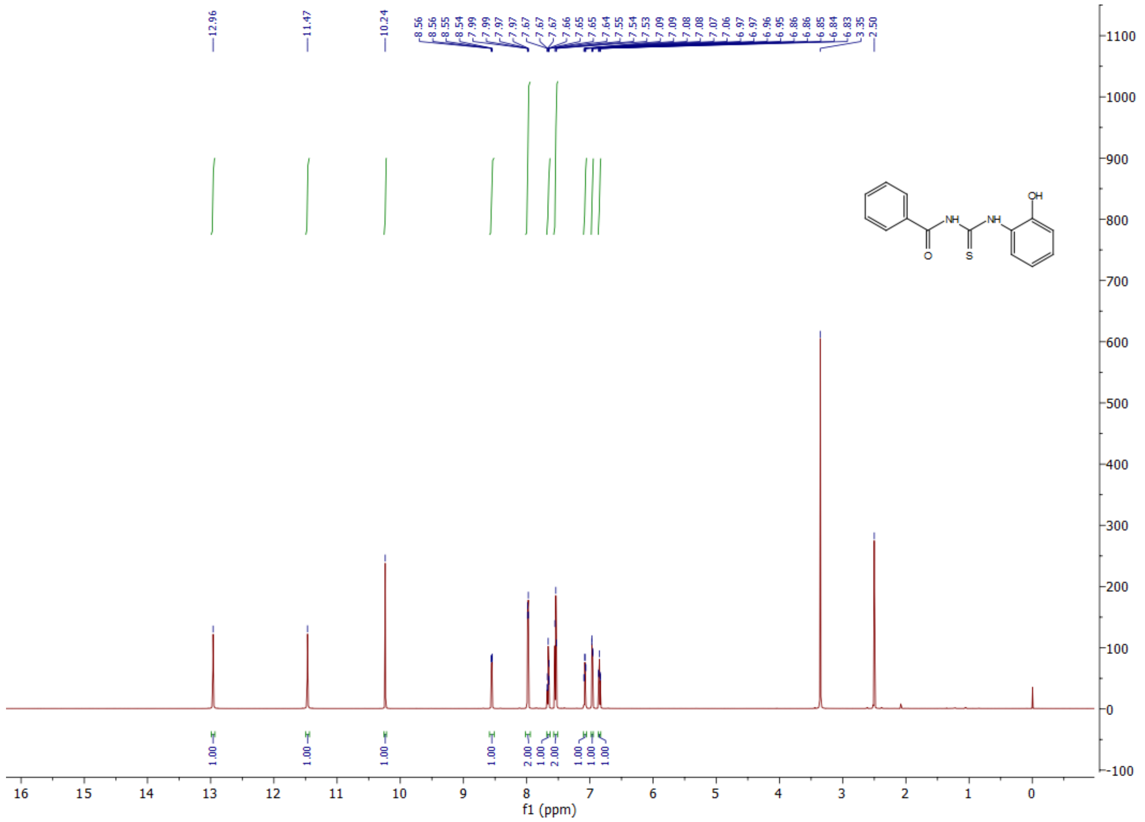

Figure S28. ^{1}H NMR (600 MHz, DMSO-d_6) spectrum of compound **8**.

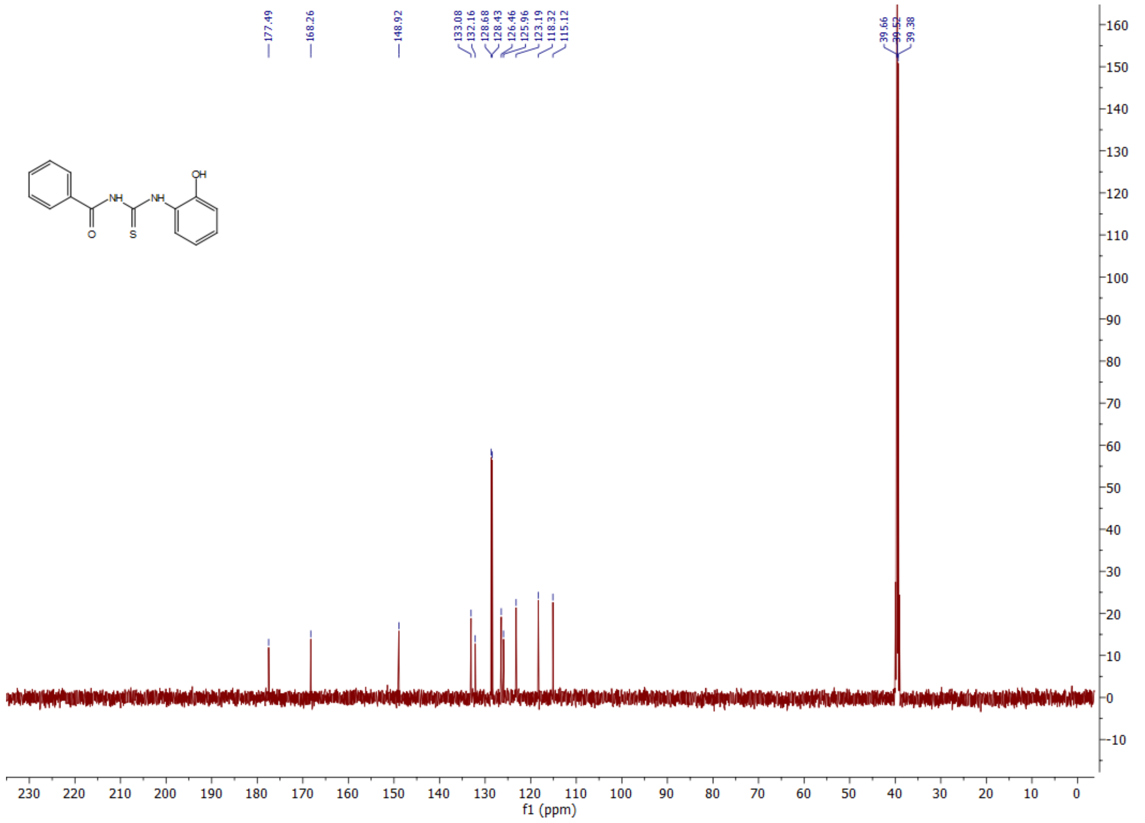

Figure S29. $^{13}C\{H\}$ NMR (150 MHz, DMSO-d_6) spectrum of compound **8**.

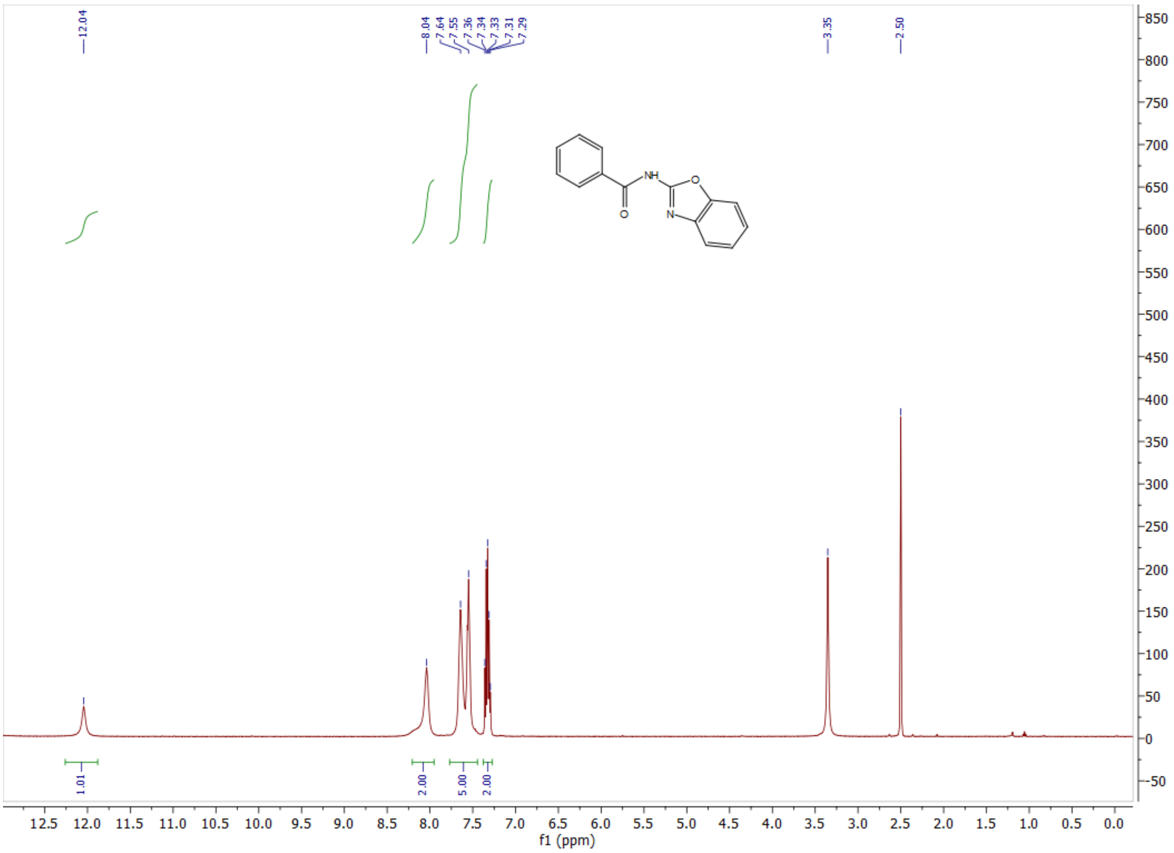

Figure S30. ^{1}H NMR (500 MHz, DMSO-d_6) spectrum of compound **10**.

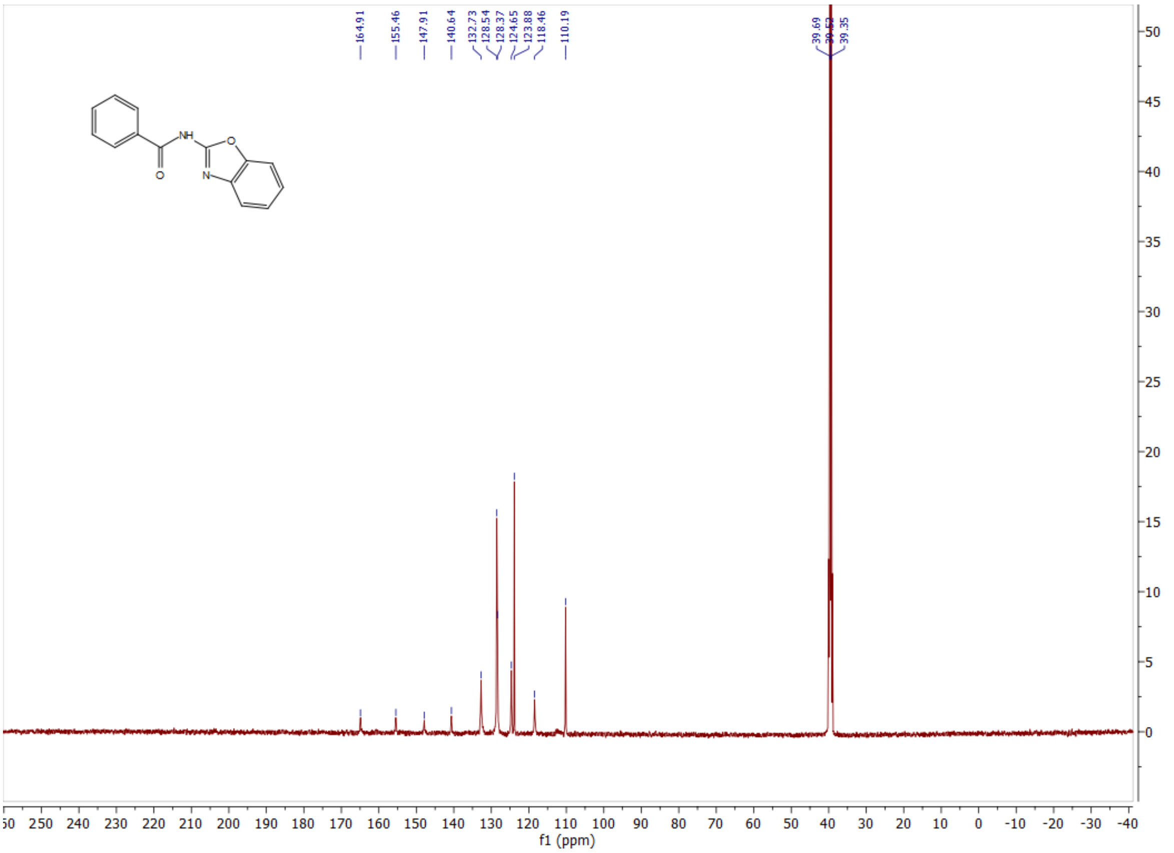

Figure S31. ^{13}C{H} NMR (125 MHz DMSO-d_6) spectrum of compound **10**.

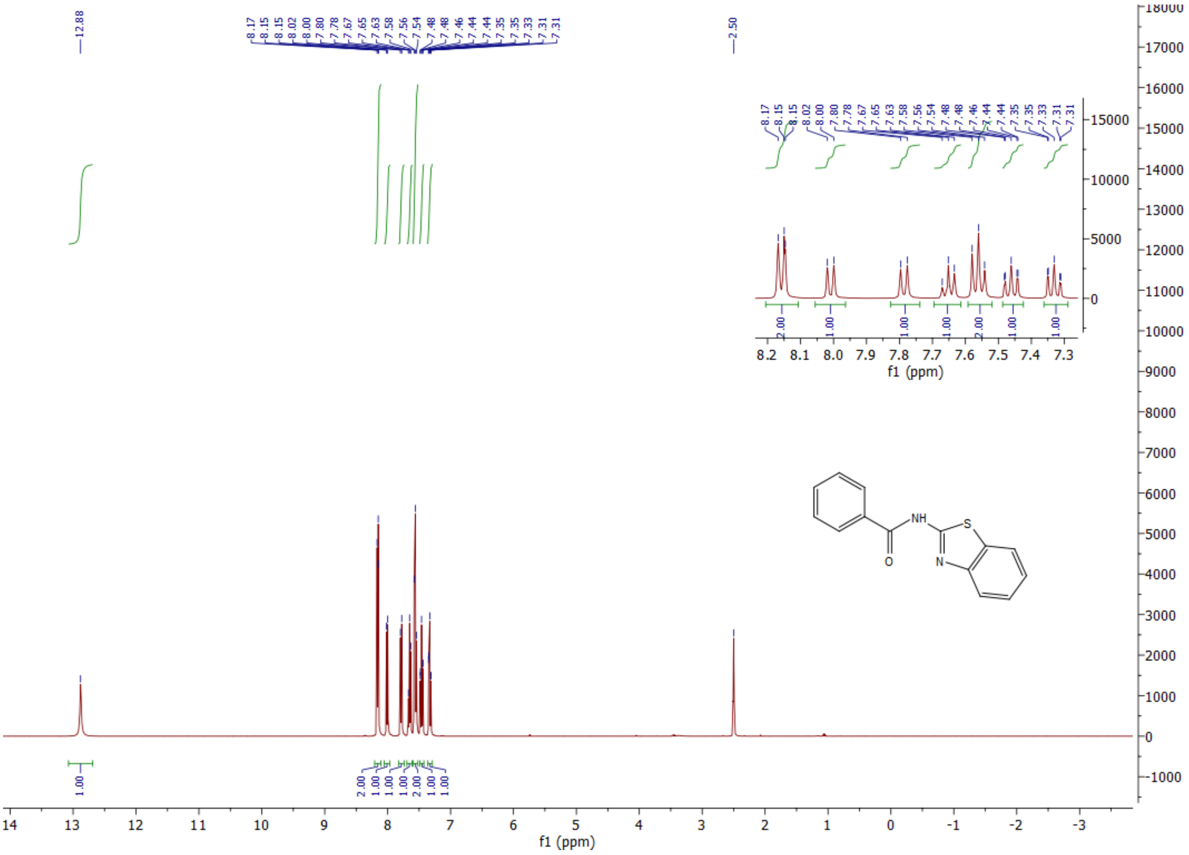

Figure S32. ^{1}H NMR (500 MHz, $CDCl_3$) spectrum of compound **13**.

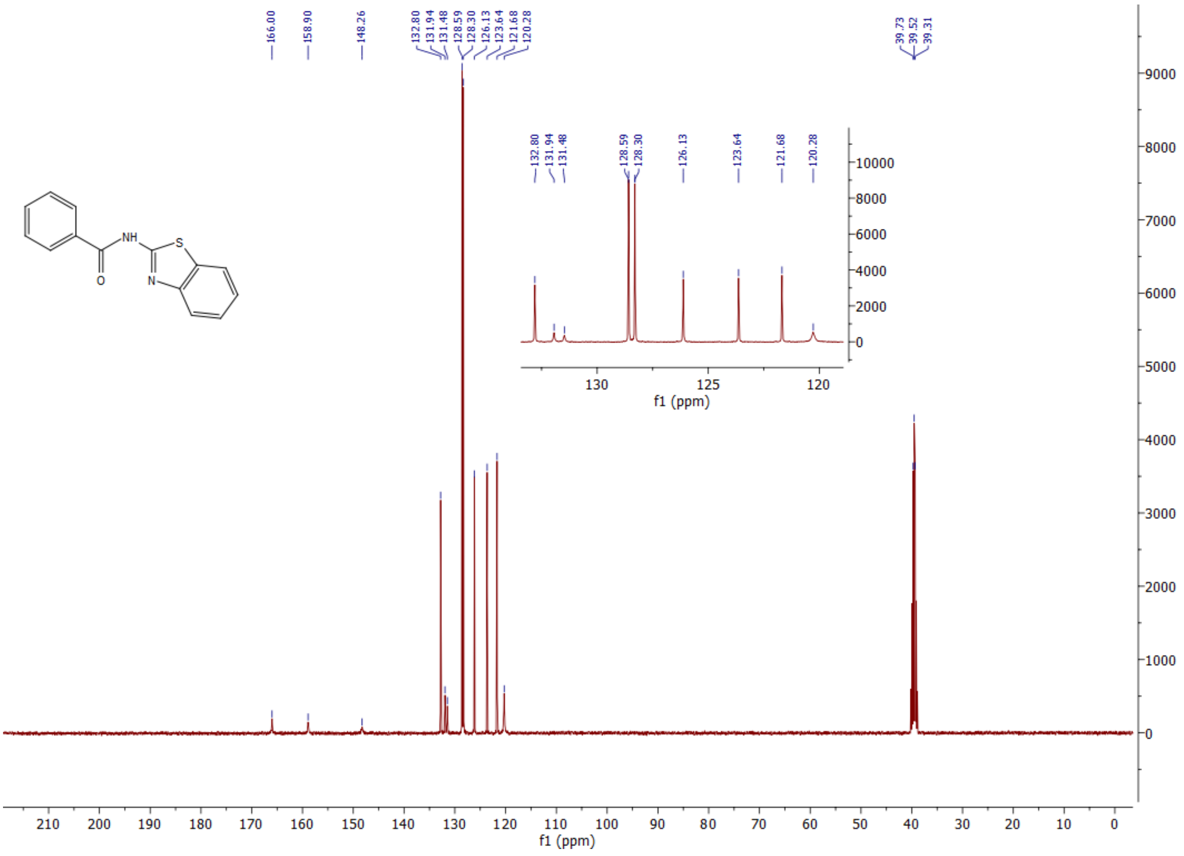

Figure S33. ^{13}C{H} NMR (125 MHz, $CDCl_3$) spectrum of compound **13**.

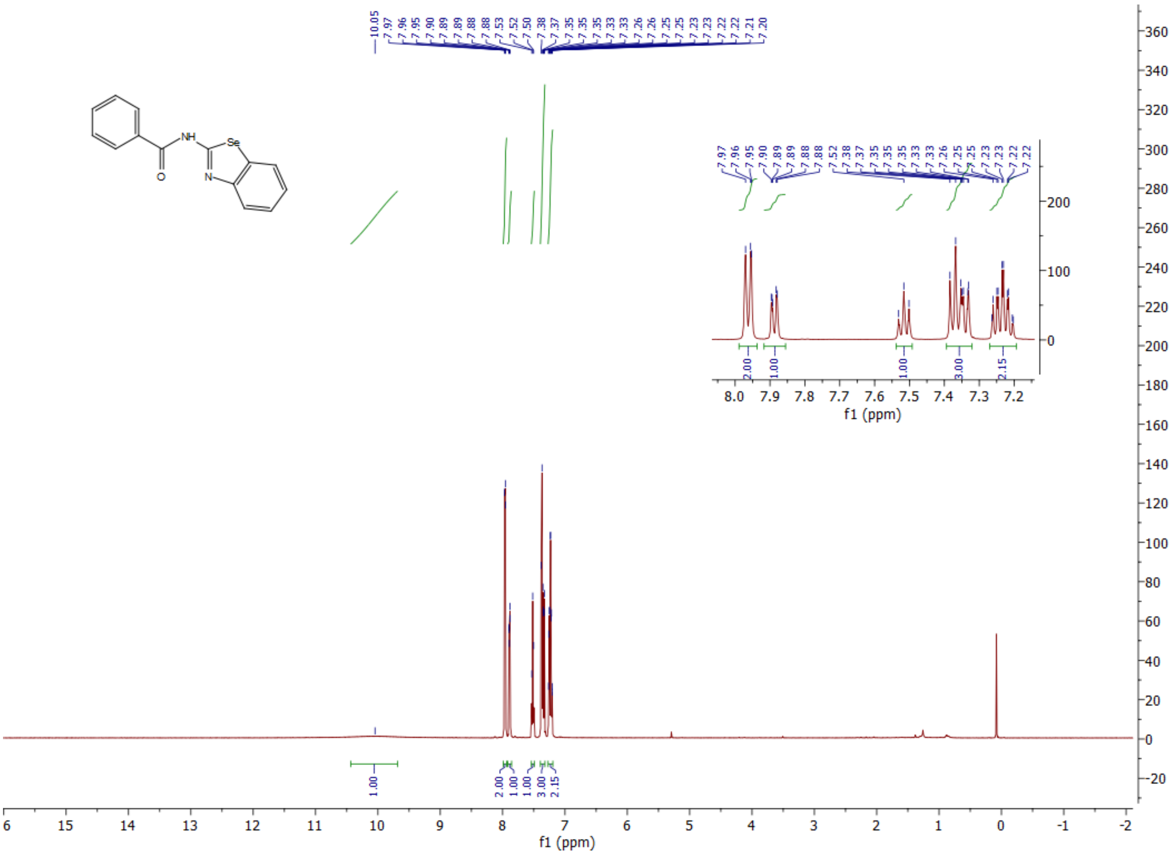

Figure S34. ^{1}H NMR (500 MHz, $CDCl_3$) spectrum of compound **16**.

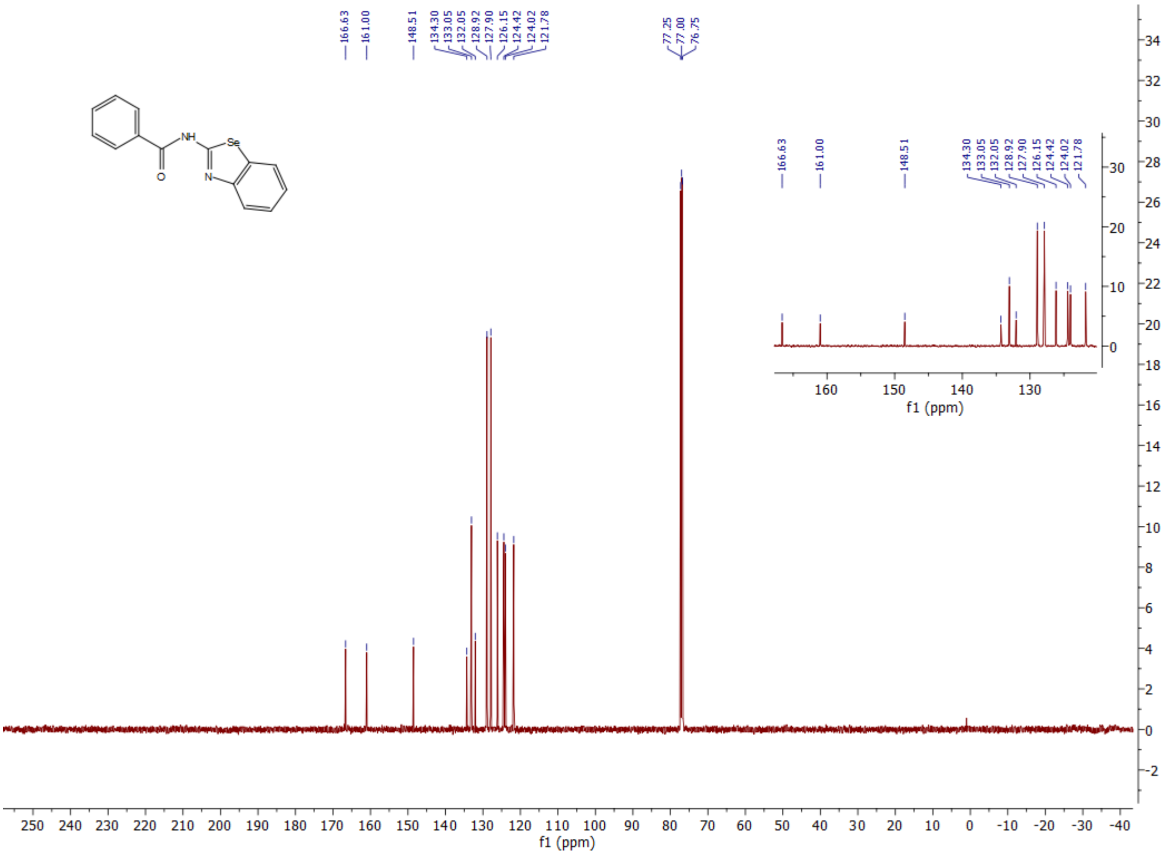

Figure S35. $^{13}C\{H\}$ NMR (125 MHz, $CDCl_3$) spectrum of compound **16**.

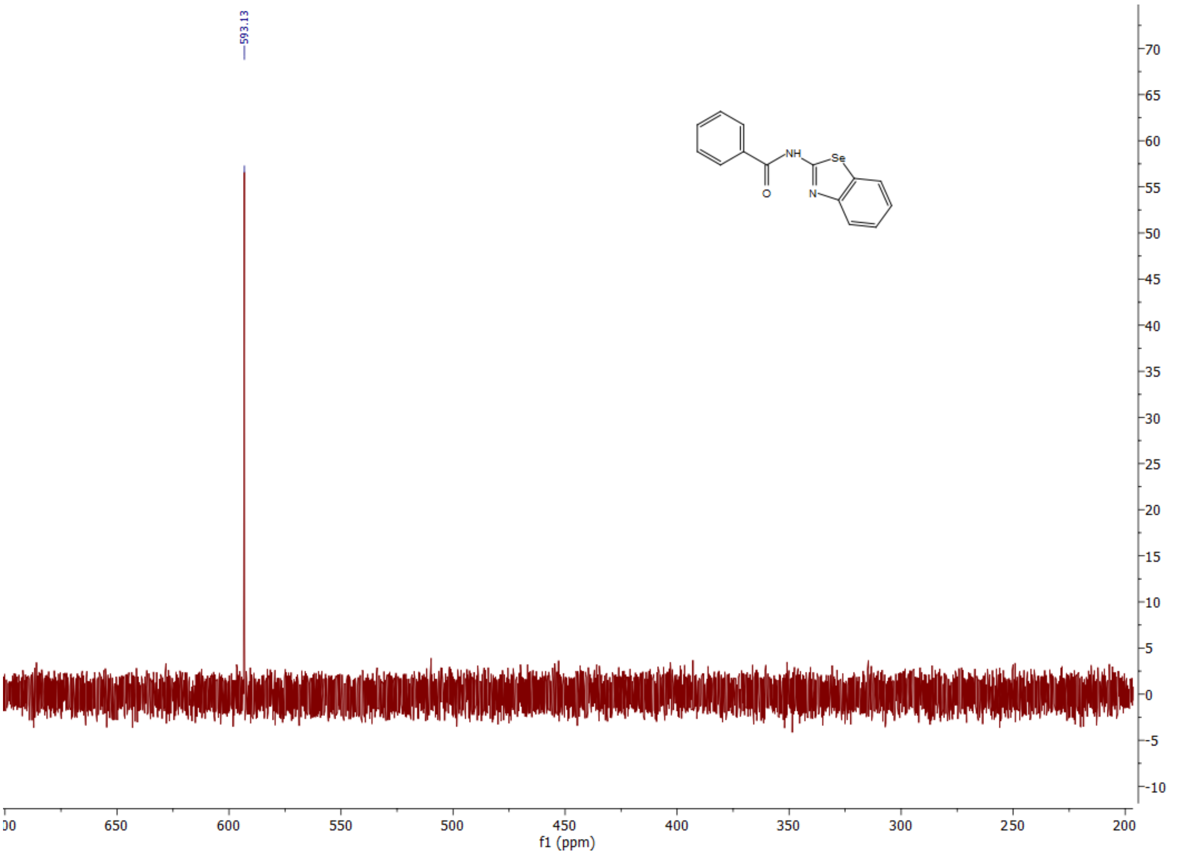

Figure S36. ^{77}Se NMR (95 MHz, $CDCl_3$) spectrum of compound **16**.

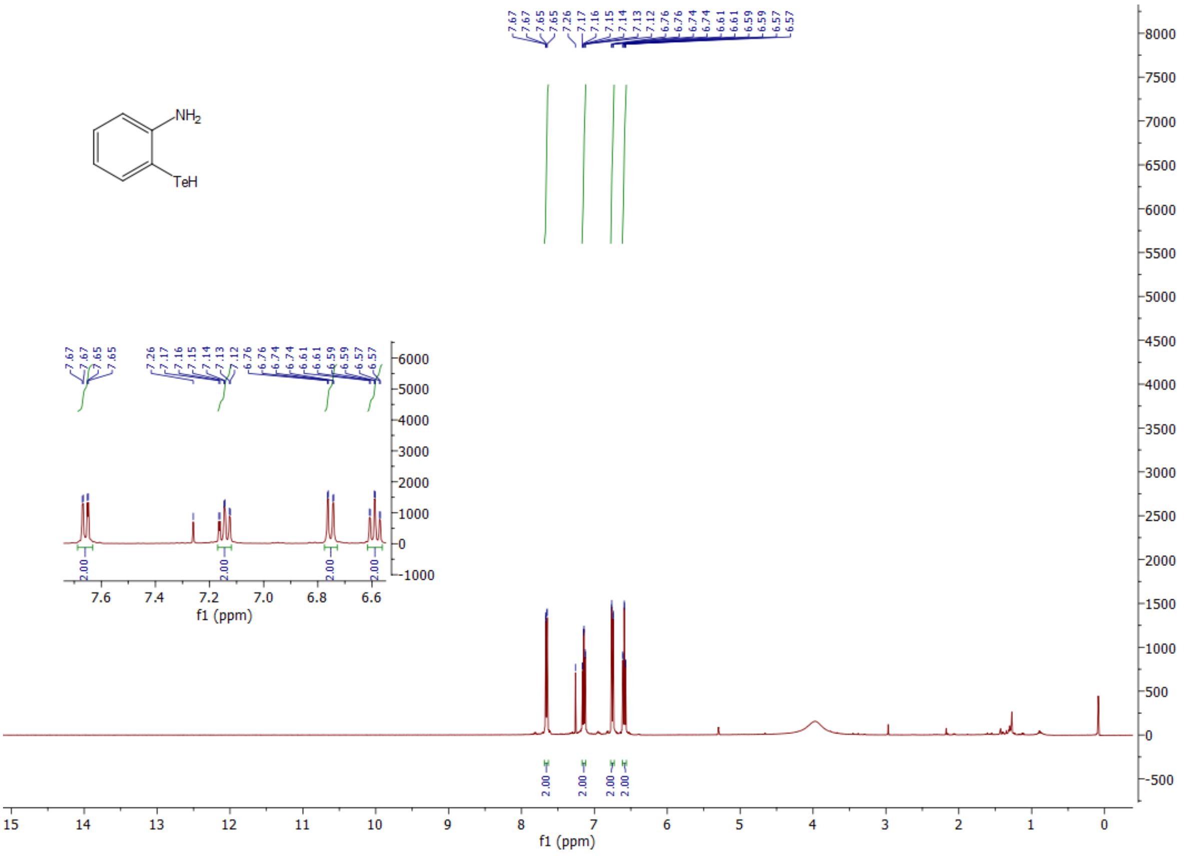

Figure S37. ^{1}H NMR (400 MHz, $CDCl_3$) spectrum of compound **17**.

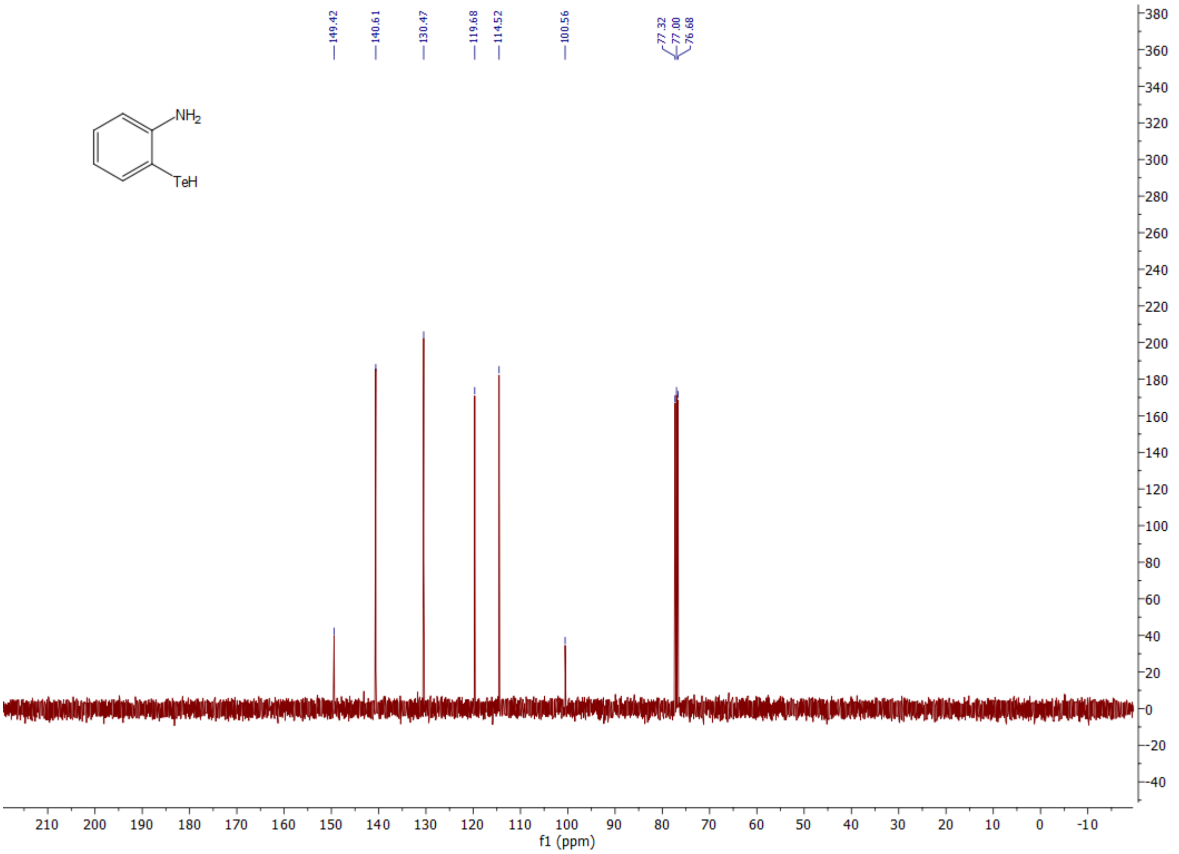

Figure S38. $^{13}C\{H\}$ NMR (100 MHz, $CDCl_3$) spectrum of compound **17**.

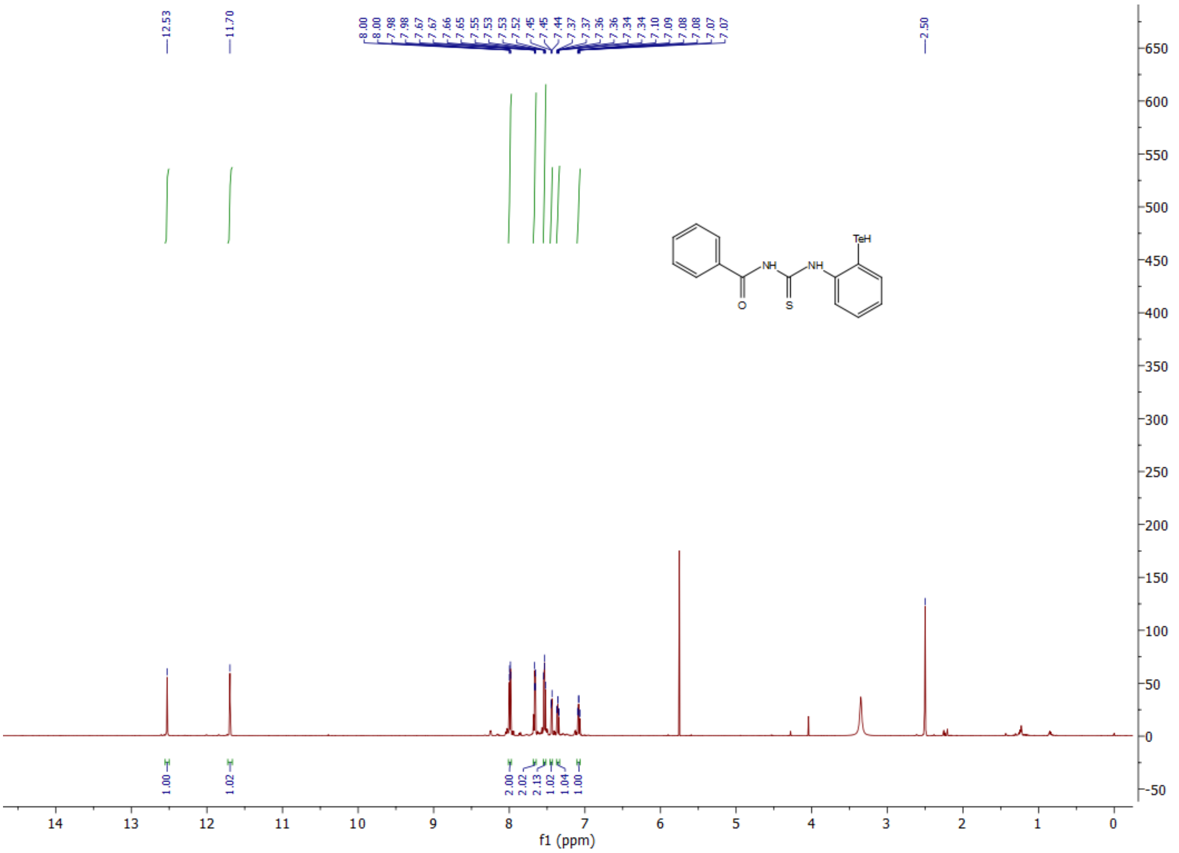

Figure S39. ^{1}H NMR (600 MHz, DMSO-d_6) spectrum of compound **18**.

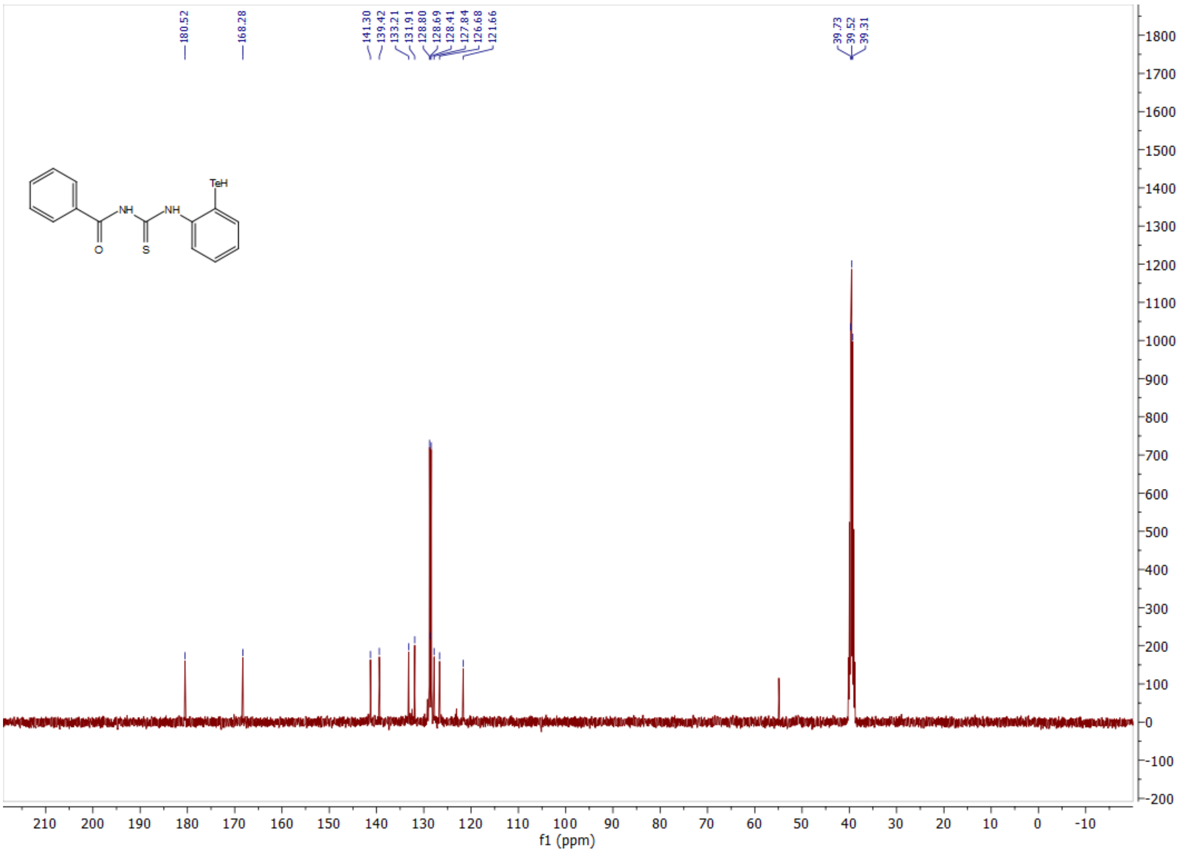

Figure S40. $^{13}C\{H\}$ NMR (100 MHz, DMSO-d_6) spectrum of compound **18**.

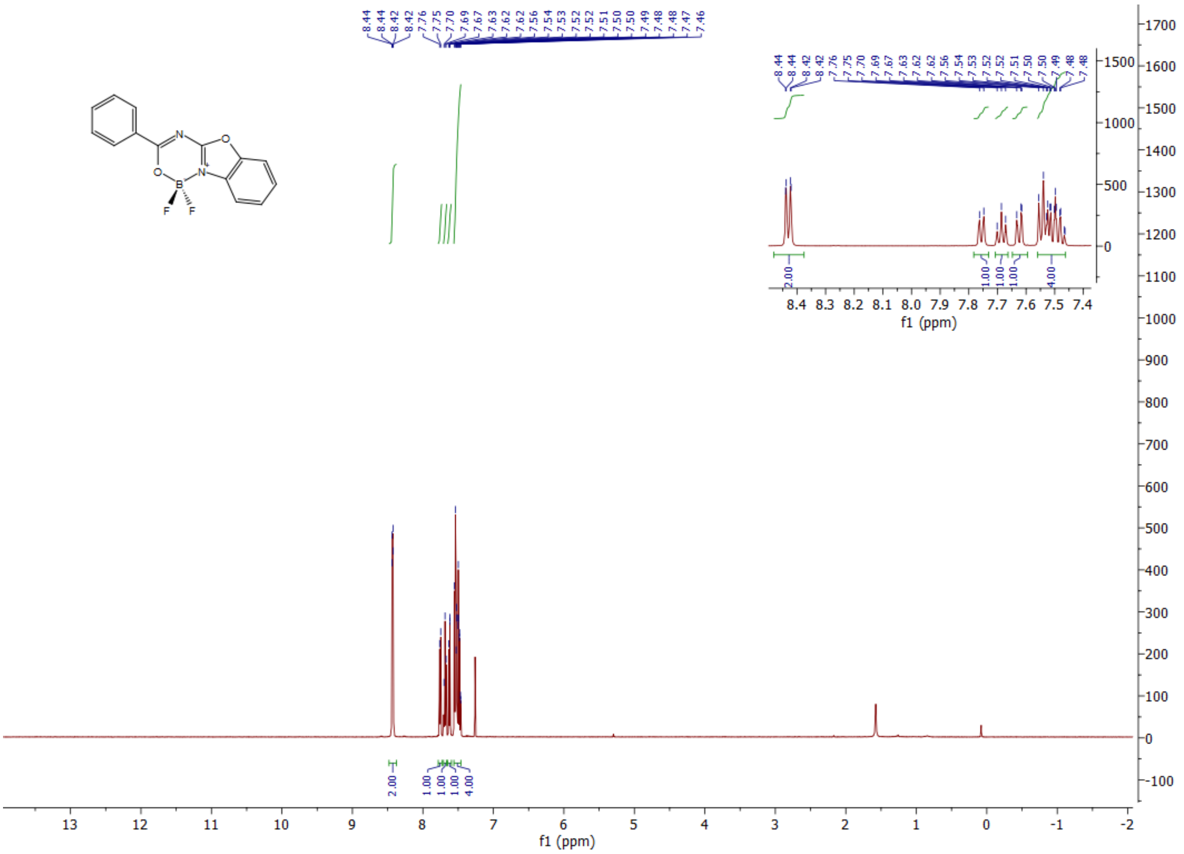

Figure S41. ^{1}H NMR (500 MHz, $CDCl_3$) spectrum of compound **1**.

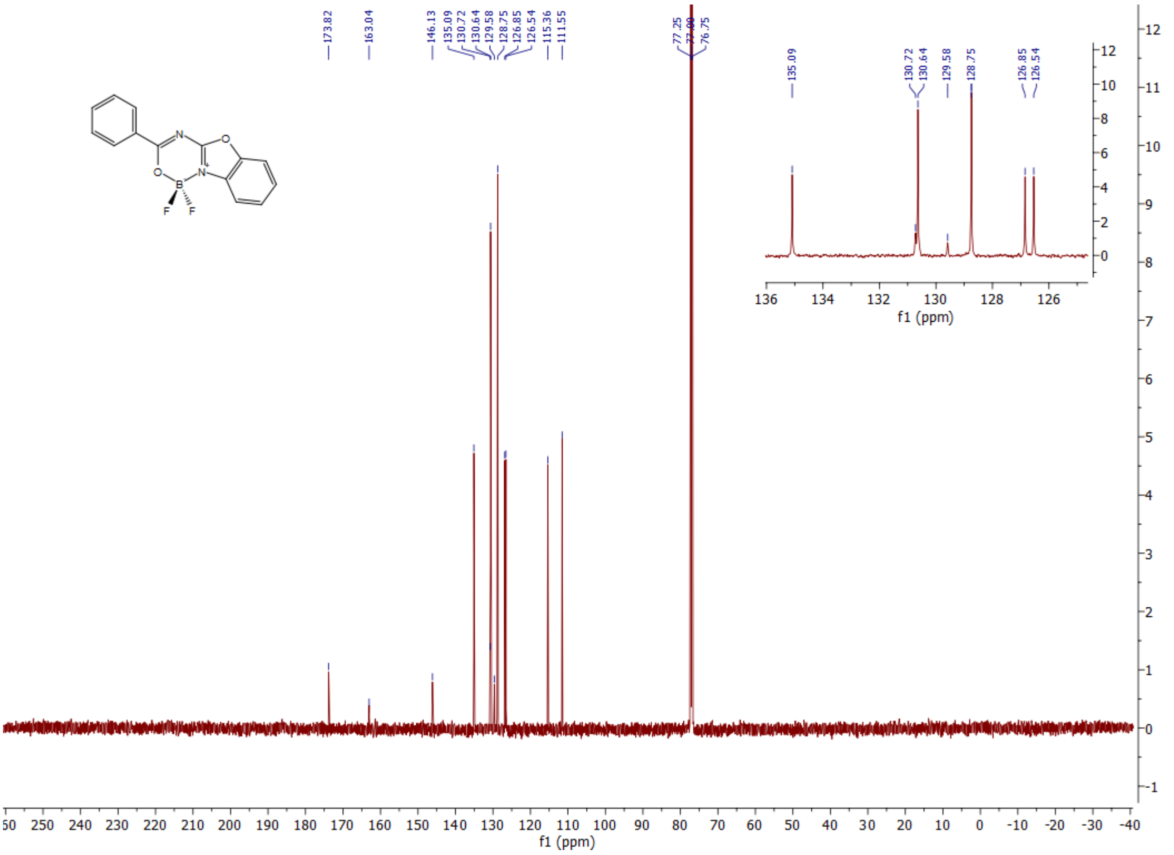

Figure S42. ^{13}C{H} NMR (125 MHz, $CDCl_3$) spectrum of compound **1**.

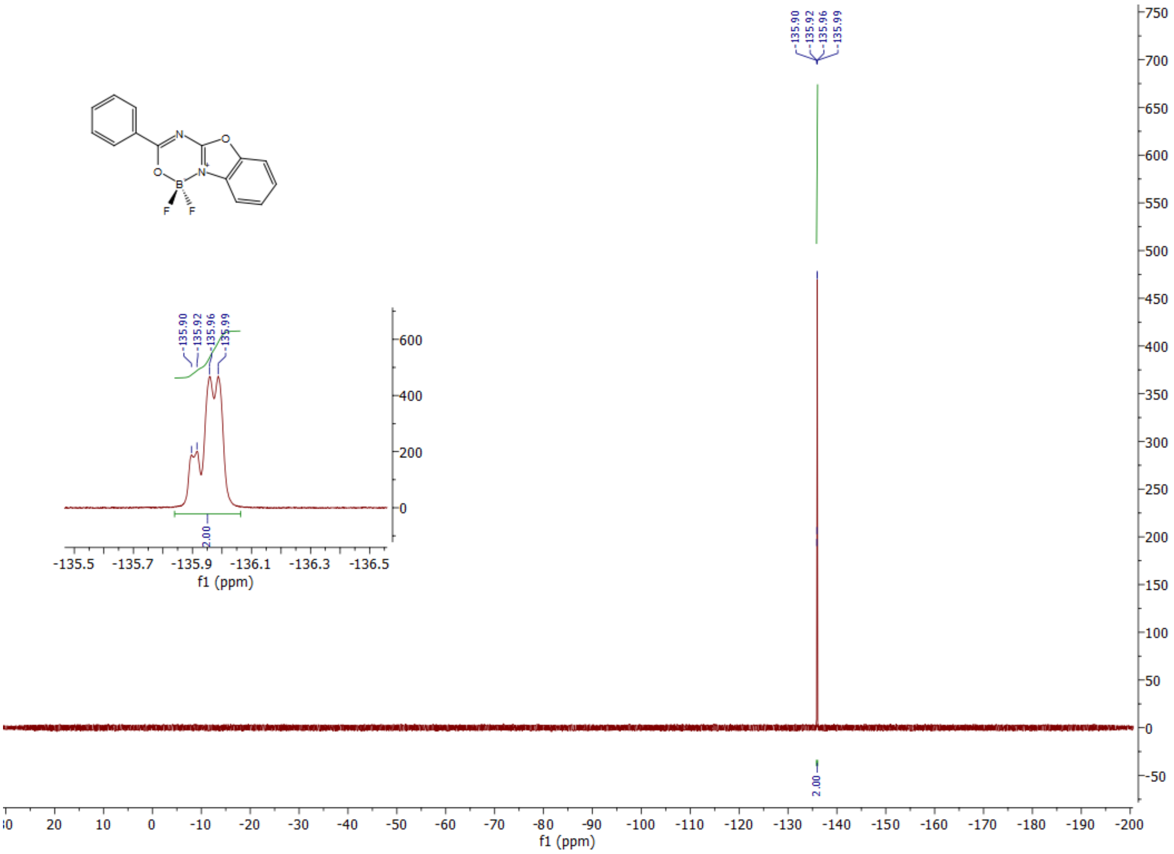

Figure S43. ^{19}F NMR (470 MHz, $CDCl_3$) spectrum of compound **1**.

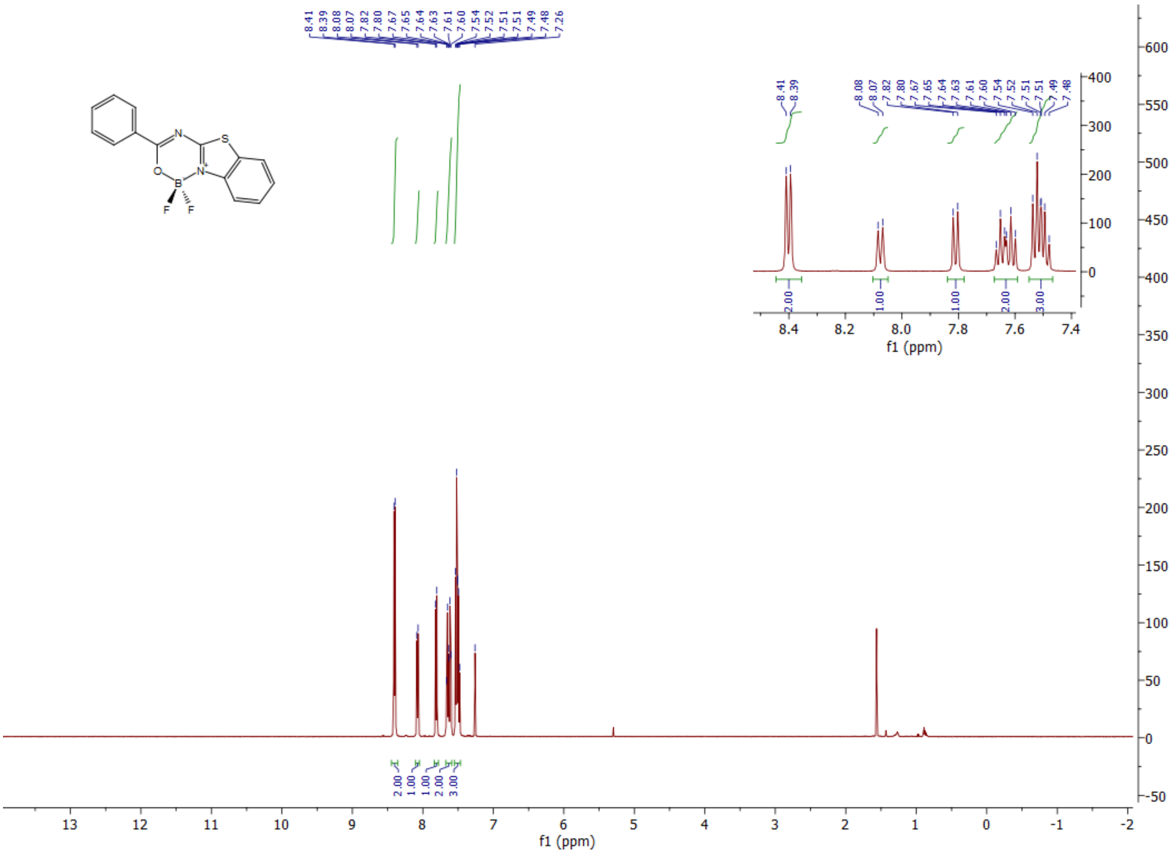

Figure S44. ^{1}H NMR (500 MHz, $CDCl_3$) spectrum of compound **2**.

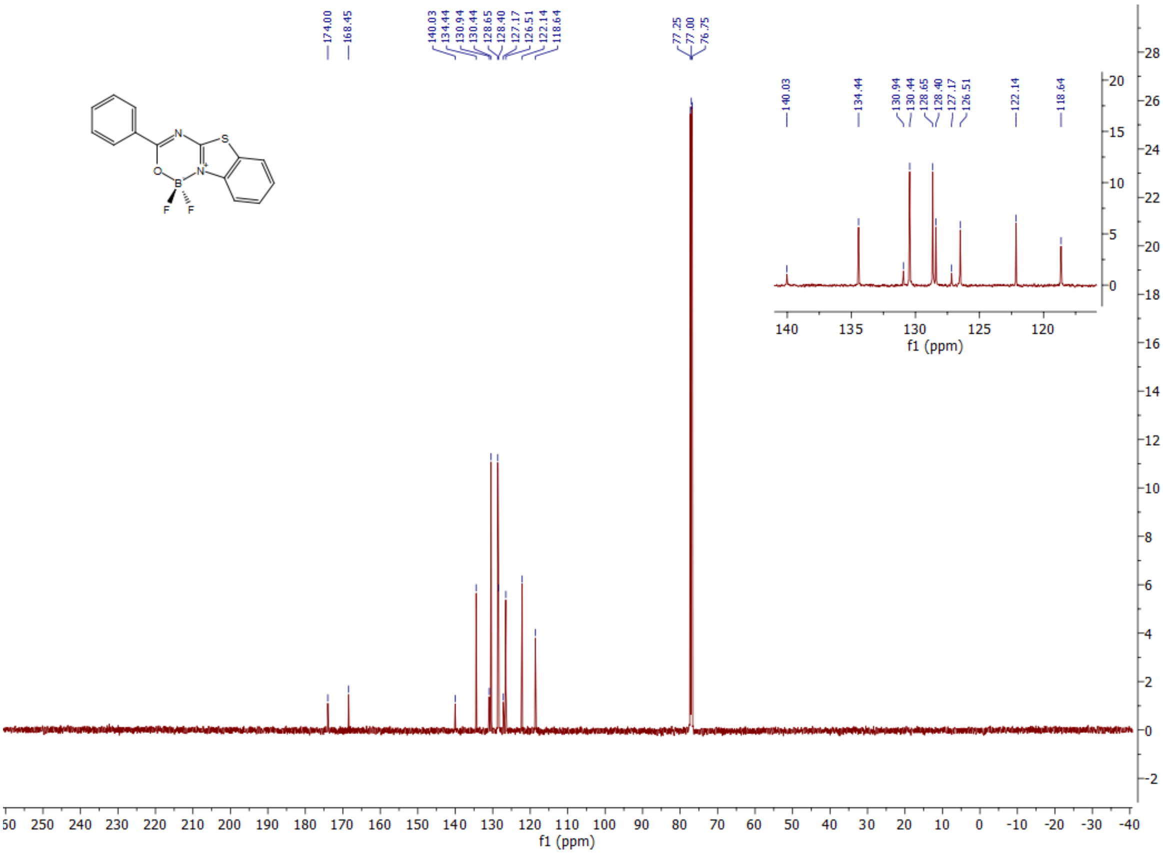

Figure S45. $^{13}C\{H\}$ NMR (125 MHz, $CDCl_3$) spectrum of compound **2**.

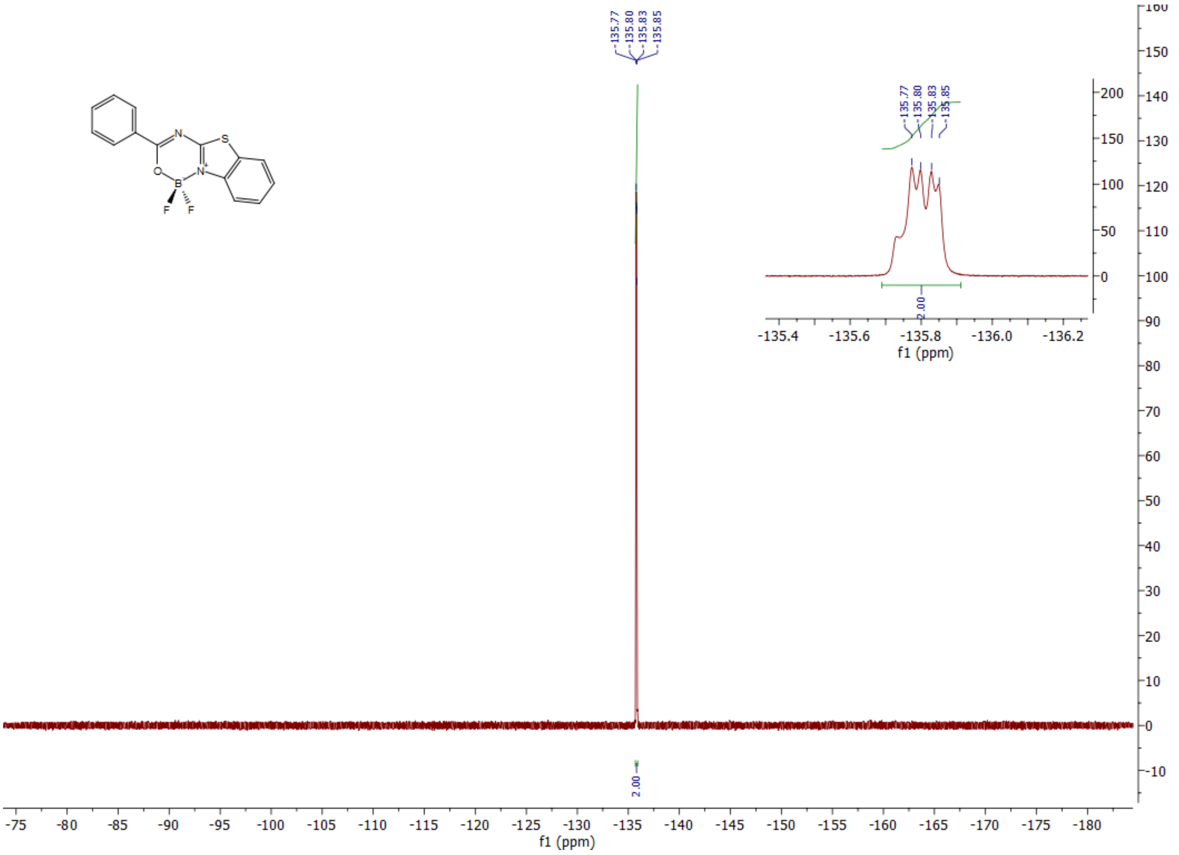

Figure S46. ^{19}F NMR (470 MHz, $CDCl_3$) spectrum of compound **2**.

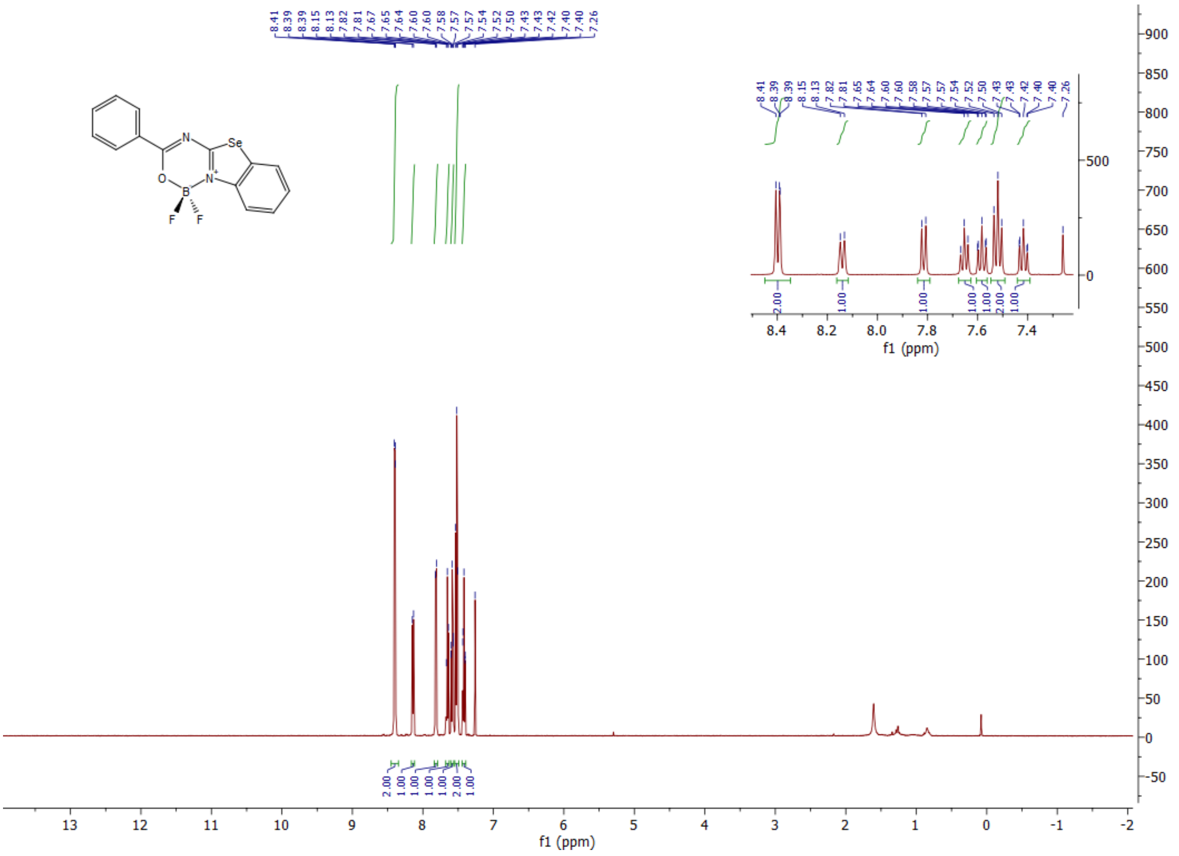

Figure S47. ^{1}H NMR (500 MHz, $CDCl_3$) spectrum of compound **3**.

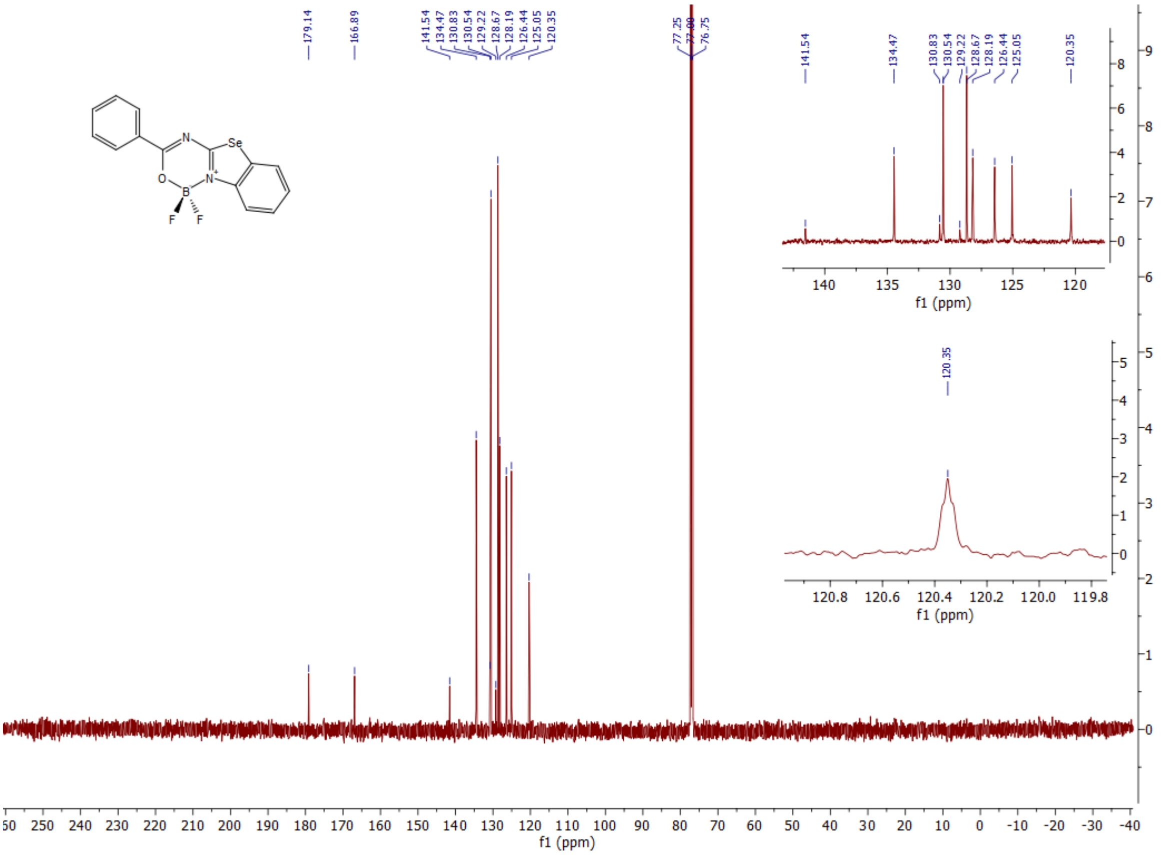

Figure S48. ^{13}C{H} NMR (125 MHz, CDCl$_3$) spectrum of compound **3**.

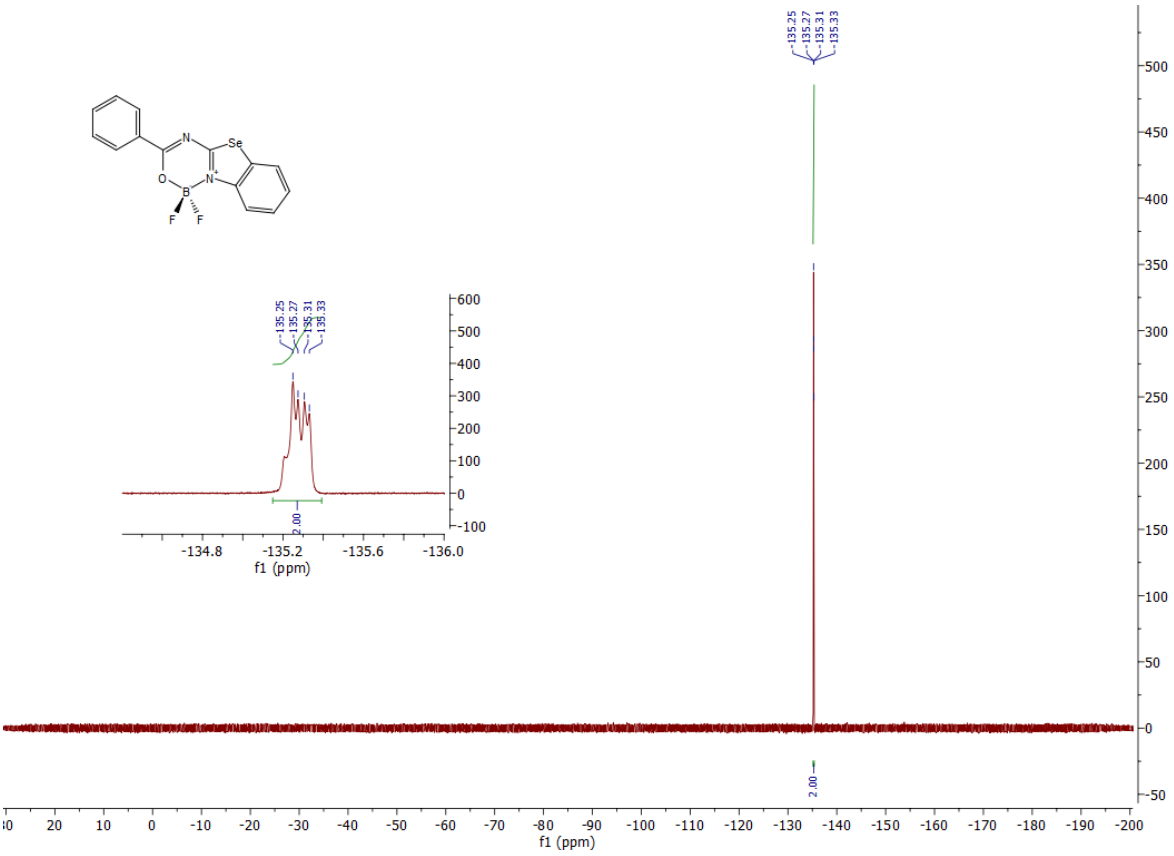

Figure S49. ^{19}F NMR (470 MHz, CDCl$_3$) spectrum of compound **3**.

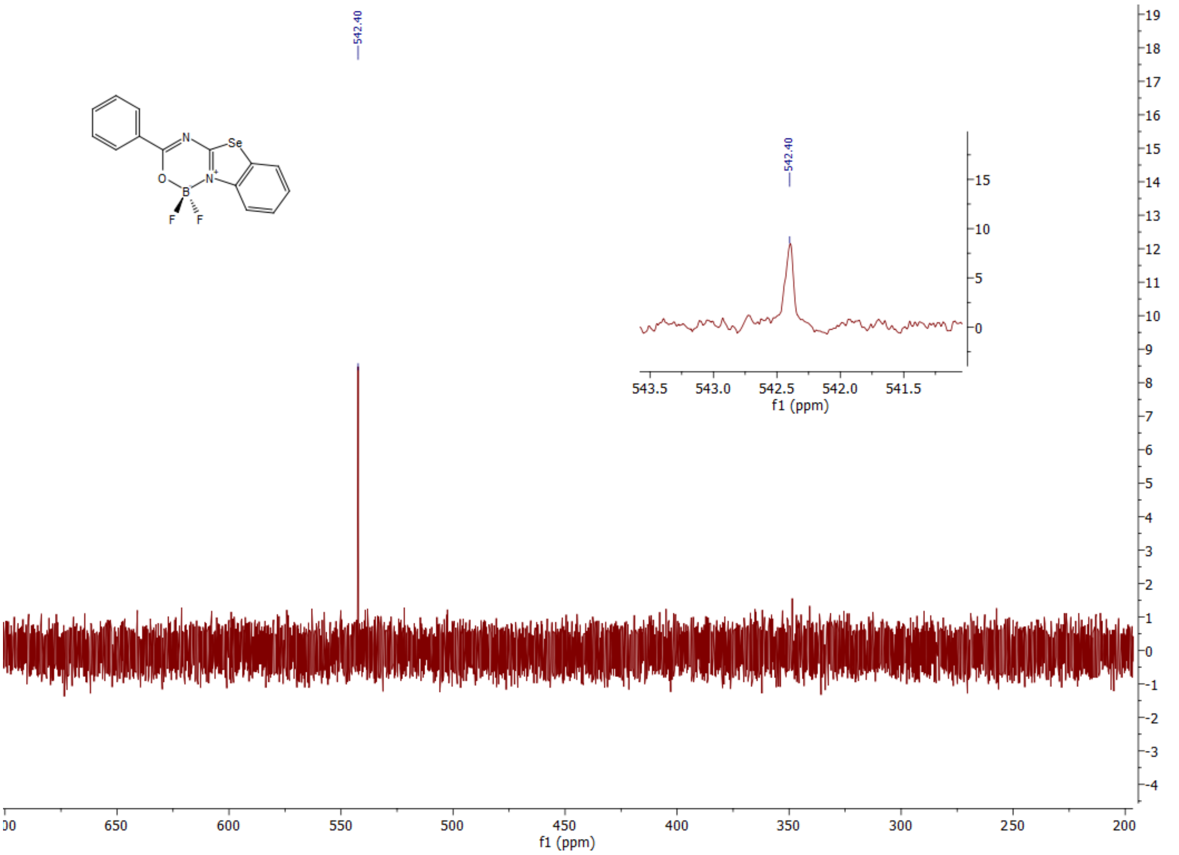

Figure S50. ^{77}Se NMR (95 MHz, $CDCl_3$) spectrum of compound **3**.

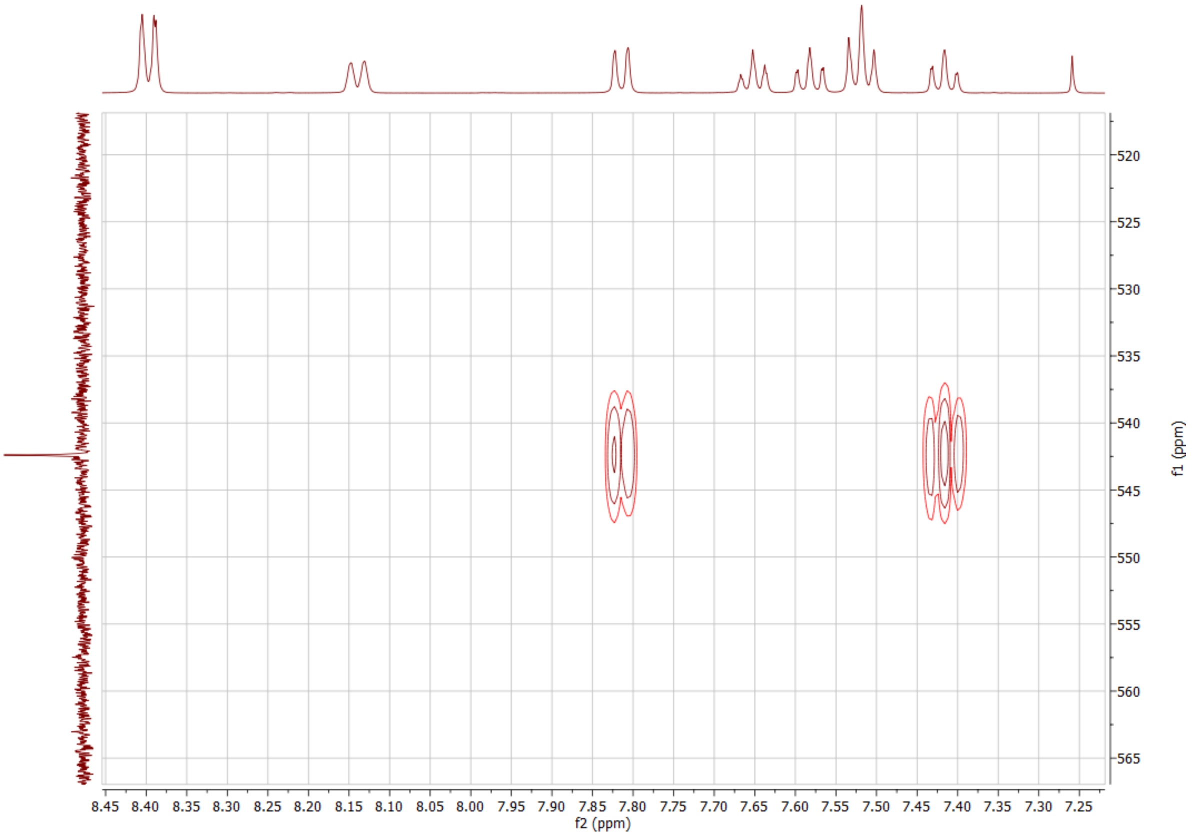

Figure S51. ^{1}H–^{77}Se HMBC NMR (500 MHz, $CDCl_3$) spectrum of **3**.

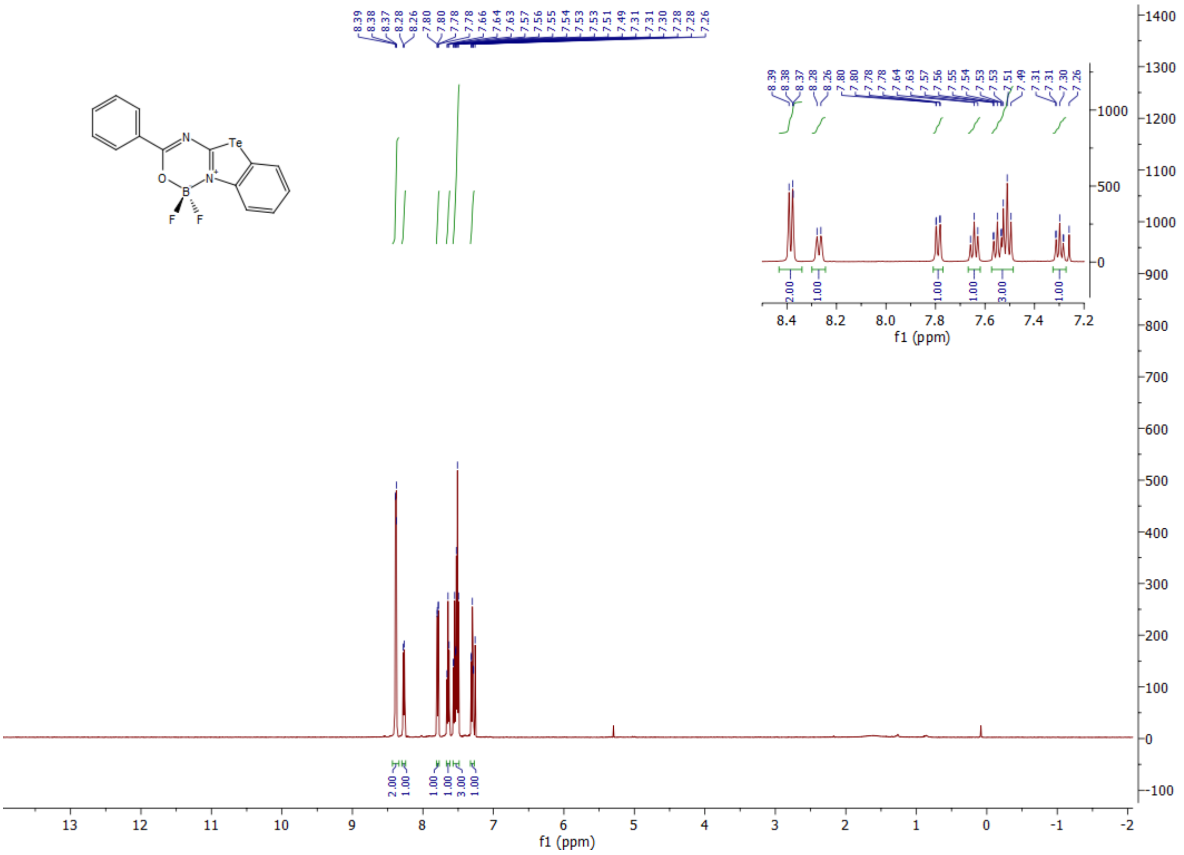

Figure S52. ^{1}H NMR (500 MHz, $CDCl_3$) spectrum of compound **4**.

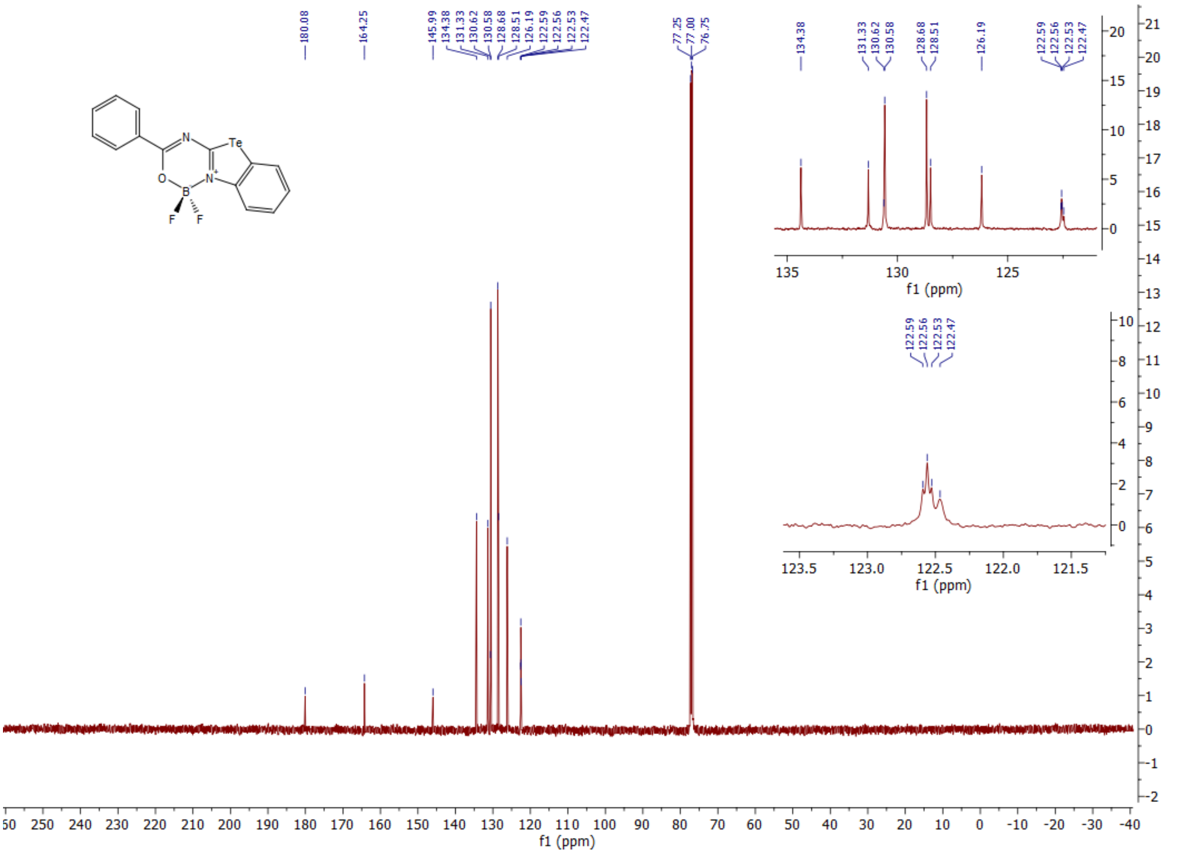

Figure S53. ^{13}C{H} NMR (125 MHz, $CDCl_3$) spectrum of compound **4**.

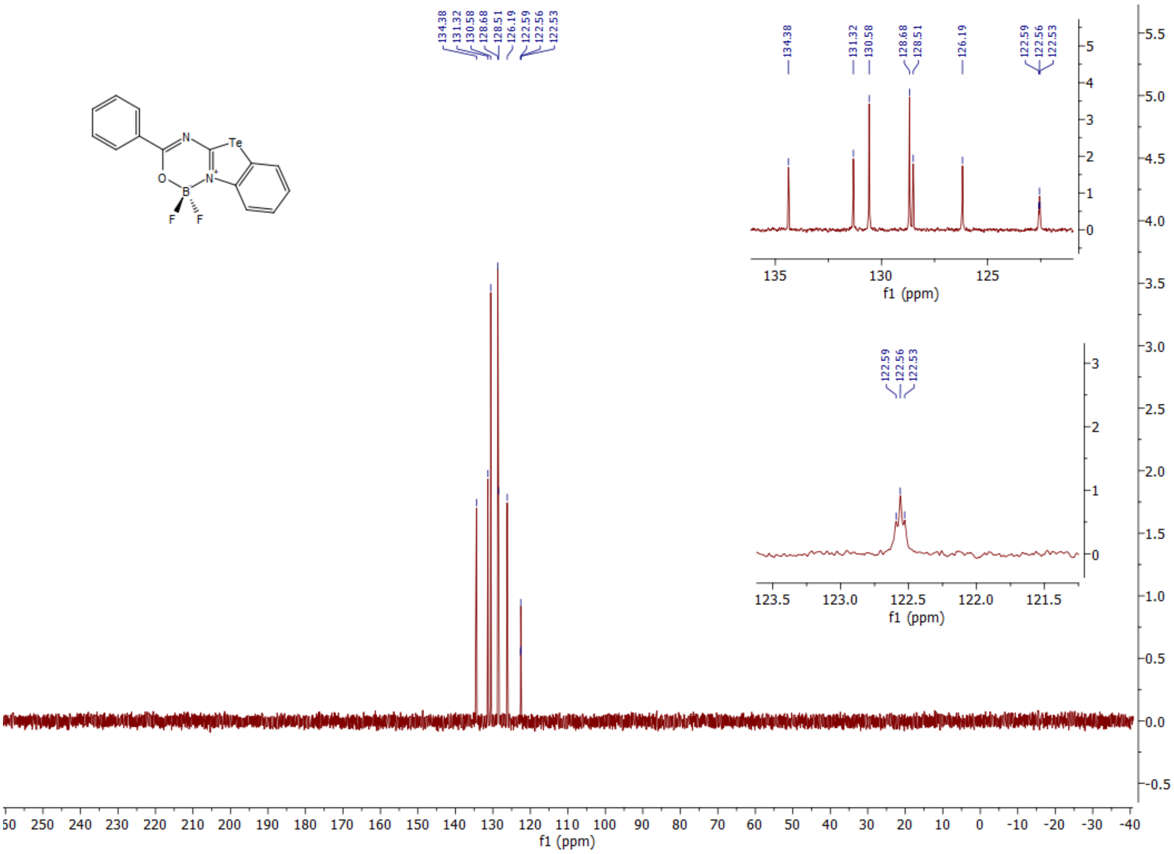

Figure S54. Detp135 NMR (125 MHz, $CDCl_3$) spectrum of compound **4**.

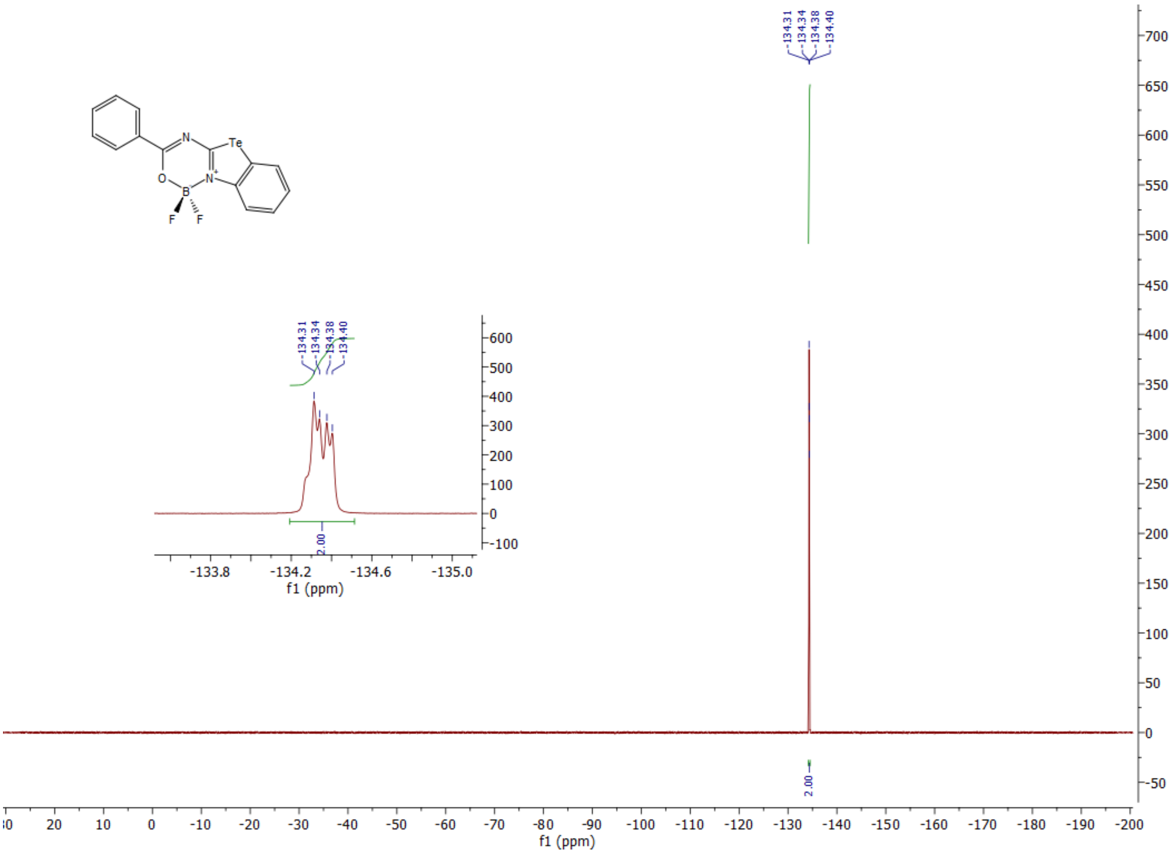

Figure S55. ^{19}F NMR (470 MHz, $CDCl_3$) spectrum of compound **4**.

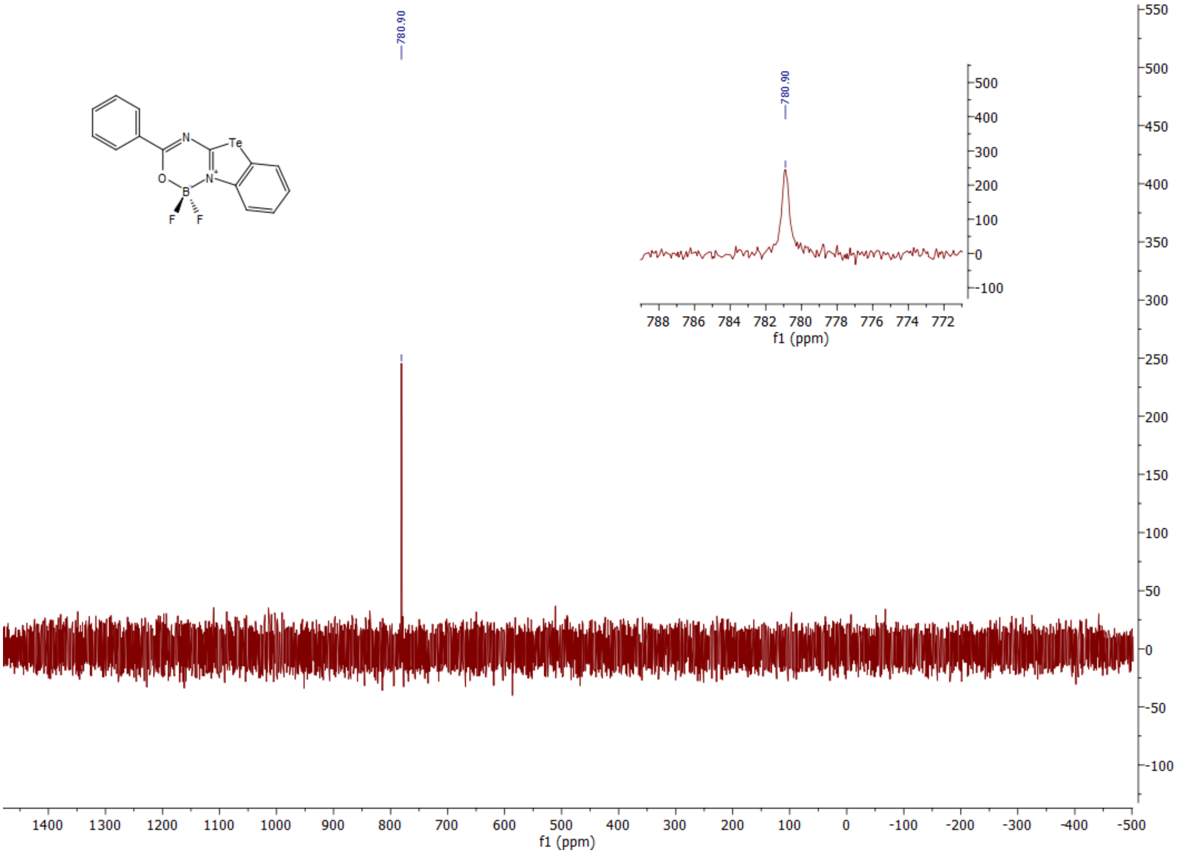

Figure S56. ^{125}Te NMR (158 MHz, $CDCl_3$) spectrum of compound **4**.

8. References

[1] Frisch, M. J.; Trucks, G. W.; Schlegel, H. B.; Scuseria, G. E.; Robb, M. A.; Cheeseman, J. R.; Scalmani, G.; Barone, V.; Petersson, G. A.; Nakatsuji, H.; Li, X.; Caricato, M.; Marenich, A. V.; Bloino, J.; Janesko, B. G.; Gomperts, R.; Mennucci, B.; Hratchian, H. P.; Ortiz, J. V.; Izmaylov, A. F.; Sonnenberg, J. L.; Williams-Young, D.; Ding, F.; Lipparini, F.; Egidi, F.; Goings, J.; Peng, B.; Petrone, A.; Henderson, T.; Ranasinghe, D.; Zakrzewski, V. G.; Gao, J.; Rega, N.; Zheng, G.; Liang, W.; Hada, M.; Ehara, M.; Toyota, K.; Fukuda, R.; Hasegawa, J.; Ishida, M.; Nakajima, T.; Honda, Y.; Kitao, O.; Nakai, H.; Vreven, T.; Throssell, K.; Montgomery, Jr., J. A.; Peralta, J. E.; Ogliaro, F.; Bearpark, M. J.; Heyd, J. J.; Brothers, E. N.; Kudin, K. N.; Staroverov, V. N.; Keith, T. A.; Kobayashi, R.; Normand, J.; Raghavachari, K.; Rendell, A. P.; Burant, J. C.; Iyengar, S. S.; Tomasi, J.; Cossi, M.; Millam, J. M.; Klene, M.; Adamo, C.; Cammi, R.; Ochterski, J. W.; Martin, R. L.; Morokuma, K.; Farkas, O.; Foresman J. B., Fox, D. J. *Gaussian 16, Gaussian, Inc., Wallingford CT*, **2016**.
